# Benchmarking CuₙO (*n* = 1, 2) complexes via ab initio methods: structural, electronic, and thermodynamic insights with biochemical relevance

**DOI:** 10.1007/s00894-025-06538-x

**Published:** 2025-12-03

**Authors:** Raúl Flores, Luis Soriano-Agueda, Marco Franco-Pérez, Rodolfo Gómez-Balderas

**Affiliations:** 1 Laboratorio de Fisicoquímica Analítica, Facultad de Estudios Superiores Cuautitlán, Unidad de Investigación Multidisciplinaria, Universidad Nacional Autónoma de México, C.P. 54700, Cuautitlán Izcalli, Estado de México Mexico; 2 Departamento de Física y Química Teórica, Facultad de Química, Universidad Nacional Autónoma de México, Cd. Universitaria, 04510 Ciudad de Mexico, Mexico

**Keywords:** Cu(II)/indomethacin, Cu(II)/acetate, Thermodynamic cycle, Theoretical protocol benchmarking, Micro-solvation

## Abstract

**Context:**

Copper is an essential trace element that plays a central role in redox chemistry and electron transfer processes in biological systems. To gain a deeper understanding of the electronic behavior of copper species, we carried out a comparative evaluation of the Cu_2_ and CuO molecules, focusing on key properties such as ionization energy, electron affinity, vibrational frequencies, bond lengths, and dissociation energies. Cu_2_ serves as a model for dinuclear copper sites present in metalloproteins like tyrosinase and hemocyanin, while CuO captures the essential features of copper-oxygen bonding relevant to copper-dependent oxidases and oxygen-activating enzymes. By systematically benchmarking density functional approximations (DFAs) against high-level CCSD(T) reference calculations or experimental data, we identify the methodologies that best reproduce the electronic and structural properties of these prototypical copper systems. The functional PBE, in particular, demonstrates the most consistent performance across both species. Insights obtained from Cu_2_ and CuO serve as a foundation for understanding more complex copper coordination environments. In this context, we extend our analysis to the Cu(II)/indomethacin complex, illustrating how the lessons learned from the fundamental systems can be applied to biologically relevant copper-ligand interactions. Overall, this study provides a systematic assessment of the accuracy of different DFAs for describing copper-containing species, establishing a solid framework for future investigations of bioinorganic copper chemistry and copper-based drug candidates.

**Methods:**

This study employed density functional theory (DFT) alongside thermodynamic cycles to assess the stability of Cu(Indo)₂ and Cu₂(Indo)₄ complexes in ethanol solution. To select suitable computational methods, a benchmark was conducted using Cu(II)/acetate complexes as reference systems. A total of fifteen DFT functionals—BPW91, PBE, B97D, revTPSS, M06-L, M11-L, B3LYP, BHandHLYP, PBE0, ωB97XD, APDF, M06, M06-2X, M06-HF, and TPSSh—were tested in combination with four basis sets: Def2-SVP, Def2-TZVP, 6–31 + G(d,p), and 6–311 + G(d,p). The most reliable functional-basis set combinations were then applied to the copper-indomethacin complexes. In addition, electronic and structural properties of Cu₂ and CuO—such as ionization potentials, electron affinities, vibrational frequencies, equilibrium bond lengths, and spin or magnetic coupling constants—were calculated. Computational results were validated through comparison with available experimental data.

**Supplementary Information:**

The online version contains supplementary material available at 10.1007/s00894-025-06538-x.

## Introduction

Copper is an essential transition metal that plays diverse and critical roles in biological systems [1–7]. It ranks as the third most abundant transition element in living organisms and participates in a wide array of biochemical processes due to its redox versatility and coordination chemistry [1]. As both a micronutrient and a component of externally administered compounds, copper contributes to fundamental physiological functions and therapeutic applications [8]. In biological systems, Cu(II) centers are key participants in electron transport chains, such as in cytochrome c oxidase, where Cu(II) facilitates the four-electron reduction of dioxygen (O₂) to water [9, 10]. Cu(II) also serves as the catalytic center in enzymes including superoxide dismutase (SOD), which mitigates oxidative stress by dismutating superoxide radicals and tyrosinase, which is involved in melanin biosynthesis and phenol oxidation [11, 12]. Copper is presented as a dinuclear center in those and other enzymes, allowing them to promote their characteristic complex redox reactions [13–15].

Emerging evidence highlights copper’s involvement in various human diseases. For instance, copper-based complexes have exhibited significant therapeutic potential, functioning as antimicrobial, antiviral, anti-inflammatory, anticancer agents, enzyme inhibitors, and chemical nucleases [16–20]. Notably, complexes formed between Cu(II) and non-steroidal anti-inflammatory drugs (NSAIDs) have shown enhanced anti-inflammatory and antiulcerogenic activity [21], along with reduced gastrointestinal toxicity compared to the parent drugs. For instance, the Cu(II)/indomethacin (Indo) complex has been reported to induce fewer gastrointestinal lesions than free Indo and potentially reduce nephrotoxicity [21–23]. Compounds such as copper-phenanthroline and copper-thiosemicarbazones have demonstrated the ability to induce apoptosis, generate reactive oxygen species (ROS), and interact with DNA and proteins, often exhibiting cytotoxicity on par with or exceeding that of cisplatin [24–27]. Conversely, elevated copper levels have been observed in certain tumors, and therapeutic strategies involving copper chelators (e.g., tetrathiomolybdate) and copper ionophores (e.g., disulfiram) have shown potential by selectively disrupting copper homeostasis in cancer cells, leading to oxidative stress and apoptosis [28–30].

In particular, molecular systems containing the CuₙO motif (*n* = 1, 2) are of considerable interest due to the well-documented high affinity of copper ions for oxygen-based ligands [31–35]. This strong interaction underpins a wide range of biological and catalytic processes, as copper plays a central role in the activation, transport, and reduction of molecular oxygen [35–37]. For example, Cu–O₂ bonding is a key feature in type III copper proteins such as hemocyanin, tyrosinase, and catechol oxidase [38, 39]. Given the importance of the Cu_n_ and CuₙO species in biology and medicine, accurate computational modeling of Cu(II) species is crucial for understanding their reactivity, spectroscopic behavior, and complexation thermodynamics. To this end, theoretical protocols must be capable of reliably predicting key properties such as geometrical parameters, ionization energies, electron affinities, vibrational frequencies, magnetic properties, and Gibbs free energies of complexation both in the gas phase and in solution.

Motivated by the biological relevance of those species, the first part of our study focuses on characterizing the electronic properties of CuO species and the Cu₂ dimer. A total of fifteen top-of-the-art density functional approximations (DFAs) were evaluated against experimental values and/or coupled-cluster CCSD(T) calculations. The properties investigated include (i) ionization energy (IE), (ii) electron affinity (EA), (iii) harmonic vibrational frequency (*ωₑ*), (iv) equilibrium bond distance (*rₑ*), (v) bond dissociation energy (*Dₑ*), (vi) spin expectation value $<{S}_{\mathrm{BS}}^{2}>$, and (vii) magnetic exchange coupling constant (J). In the second phase of the study, we benchmarked a preselected set of DFAs using the dimerization of Cu(II)/acetate and Cu₂/acetate complexes as model systems. These served to assess the DFAs’ accuracy in predicting the Gibbs free energy of complexation in both the gas phase and solution, using thermodynamic cycles, a widely used strategy to estimate the stability of metal–ligand complexes, given its high accuracy and computational efficiency. Finally, as an application case, we extended the best-performing DFAs to evaluate the dimerization energy of the Cu(II)/indomethacin (Indo) complex in ethanol, providing an estimate of the Gibbs free energy of complexation in a biologically relevant solvent environment.

## Models and procedures

### Electronic properties

To evaluate the capability of a range of theoretical protocols to compute molecular properties of the interest of this study, geometry optimizations for ${\mathrm{Cu}}^{\mathrm{q}}$, ${\mathrm{Cu}}_{2}^{\mathrm{q}}$, and ${\mathrm{CuO}}^{\mathrm{q}}$ (*q* = 0, + 1, –1) species were carried out with the following DFAs, at four different level of sophistications: (1) GGA: BPW91 [40, 41], PBE [42], and B97D [43]; (2) meta-GGA: revTPSS [44], M06-L [45], and M11-L[46]; (3) hybrid DFAs: B3LYP [47], BHandHLYP [48], APFD [49], ωB97XD [50], and PBE0 [51]; and (4) hybrid meta-GGA: M06 [52], M06-2X [52], M06-HF [53, 54], and TPSSh [55]. At this stage, four basis sets were tested: the Ahlrichs Def2-SVP and Def2-TZVP [56, 57] and Pople 6–31 + G(d,p) and 6–311 + G(d,p) basis [58–62]. These basis sets have shown remarkable performance in the computation of several molecular properties of copper containing species when combined with a wide range of DFAs, for instance, those considered in this study [63–65]. We ensure that our optimized geometries corresponded to a minimum on the potential energy surface (PES) through the corresponding analysis of vibrational frequencies.

The ionization energy (IE) and electron affinity (EA) of the ${\mathrm{Cu}}_{2}$ and $\mathrm{CuO}$ were obtained accordingly to the following definitions:

1 $$\begin{array}{c}IE=E\left(\mathrm{N}-1\right)-E\left(\mathrm{N}\right),\end{array}$$

2 $$\begin{array}{c}EA=E\left(\mathrm{N}\right)-E\left(\mathrm{N}+1\right),\end{array}$$

respectively, where E(N) is the energy of the complex in the reference state with N electrons; E(N − 1) and E(N + 1) are the energies of the complex with one less or one more electron, respectively. These calculations were performed in both adiabatic and vertical fashions. For the adiabatic case, geometry optimizations were conducted for charged species using the range of level of theories specified above. The outcomes of these calculations were used to extract vibrational constants ${\omega }_{e}$, equilibrium distances ${r}_{e}$, and dissociation energies ${D}_{e}$ of the ${\mathrm{Cu}}_{2}^{\mathrm{q}}$ and ${\mathrm{CuO}}^{\mathrm{q}}$ (*q* = 0, + 1, –1) species.

The expected value of the $<{S}_{\mathrm{BS}}^{2}>$ operator and the coupling constant $J$ were computed for ${\mathrm{Cu}}_{2}{\mathrm{AcO}}_{4}$ using the broken symmetry approach (BS-DFT) proposed by Ginsberg et al. [66–69]. Due to the limited performance of some functionals in reproducing the previously analyzed properties, these magnetic calculations were carried out using a reduced set of thirteen DFAs (out of the original fifteen).

### Thermodynamic cycles

One of the most effective and computationally efficient strategies for calculating reaction Gibbs free energies in solution is the use of thermodynamic cycles (TCs) [70–74]. These methods strike an excellent balance between computational cost and agreement with experimental data and their use in computing complexation Gibbs free energies in solution phase involving transition metallic ions, particularly those belonging to the *3d* series, is well documented [70, 71, 75]. Reactions in solution involve an enormous number of degrees of freedom, making a fully explicit quantum mechanical treatment of all solute–solvent interactions impractical. Accurate modeling of the solvation environment for each participating species is therefore a central challenge. TCs typically employ a hybrid solvation strategy that combines continuum and explicit solvent models. The continuum model treats the solvent as a polarizable medium characterized by its dielectric constant, capturing the bulk electrostatic contributions to the solvation free energy. This approximation is particularly effective for modeling long-range solvation effects and works well for neutral solutes, especially when integrated within a thermodynamic cycle framework or when a chemical process occurs under the presence of a universal solvent [76]. To account for short-range, specific interactions that the continuum model cannot capture—such as hydrogen bonding or directional electrostatics—a micro-solvation step is introduced, which is crucial when dealing with charged species [75]. Here, a small number of explicit solvent molecules are strategically placed around each solute to reflect key solute–solvent and solvent–solvent interactions at the molecular level. Remarkably, TC-based strategies are suitable for maximizing error cancellation and have recently been used to accurately describe the formation thermodynamics of some Cu(II) and Zn(II) complexes in water and ethanol solutions, respectively [70, 71].

**Fig. 1:**
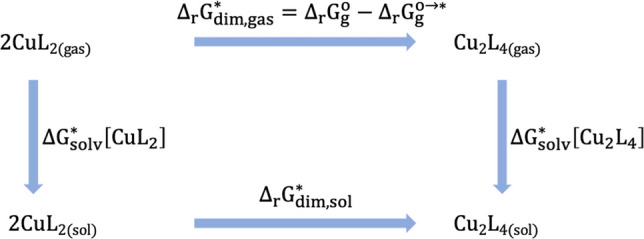
Thermodynamic cycle to estimate the dimerization free energies in solution phase, the ligand L is either acetate or anionic indomethacin

In this context, thermodynamic cycles (TCs) offer a particularly suitable and convenient framework for computing the Gibbs free energy associated with dimerization processes involving neutral monomers. By avoiding the inclusion of charged species, which can introduce significant errors in solvation energy estimates, this approach enhances the reliability of the calculated values in solution phase. Figure 1 depicts the TC employed in this study to determine the Gibbs free energy of the dimerization reactions under investigation, where it is assumed that the complexation process precedes the dimerization of the metallic center. Since the overall neutral charge is maintained throughout the dimerization process, short-range specific interactions are expected to remain nearly constant, thereby justifying the omission of explicit solvent molecules in the analysis.

Under these circumstances, the computation of the dimerization Gibbs free energy in solution phase using the TC in Fig. 1 requires corrections related to the free energy variation due to the change in the solute standard state, from the standard state of ideal gas (1 atm) to the standard state of solution (1 M) both at 298.15 K, ${\Delta \mathrm{G}}^{0\to *}$ [77, 78]:

3 $$\begin{array}{c}{\Delta G}^{0\to *}=-{T\Delta S}^{0\to *}=RTln\left(\frac{{V}_{0}}{{V}^{*}}\right)=RTln\left(24.46\right) .\end{array}$$

Introducing this correction to the gas phase contribution to the solution phase Gibbs free energy of dimerization provides

4 $$\begin{array}{c}{\Delta }_{r}{G}_{\mathrm{dim},\mathrm{gas}}^{*}={\Delta }_{r}{G}_{\mathrm{gas}}^{0}+{\Delta }_{r}nRT\mathrm{ln}\left(24.46\right)\end{array}$$

where ${\Delta }_{r}{G}_{\mathrm{gas}}^{0}$ represents the uncorrected standard Gibbs free energy of dimerization in the gas phase, which can be directly obtained from thermochemical data provided by standard electronic structure calculations. The term ${\Delta }_{r}n$ denotes the change in the number of moles (amount of substance) between products and reactants (− 1) in the balanced chemical equation. This way, the Gibbs free energy of dimerization in solution phase, ${\Delta }_{r}{G}_{\mathrm{dim},\mathrm{sol}}^{*}$, is computed as

5 $$\begin{array}{c}{\Delta }_{r}{G}_{\mathrm{dim},\mathrm{sol}}^{*} \left({\mathrm{CuL}}_{2}\right)={\Delta }_{r}{G}_{\mathrm{dim},\mathrm{gas}}^{*}\left({\mathrm{CuL}}_{2}\right)+{\Delta G}_{\mathrm{solv}}^{*}\left({\mathrm{Cu}}_{2}{\mathrm{L}}_{4}\right)-{2\Delta G}_{\mathrm{solv}}^{* }\left({\mathrm{CuL}}_{2}\right)\end{array}$$

where ${\Delta G}_{\mathrm{solv}}^{*}\left(X\right)$ is the Gibbs free energy of solvation of the species *X* immersed in a particular solvent, which can be conveniently estimated from a continuum approach, since this strategy does not contemplate charged species.

It is important to note that ${\Delta }_{r}{G}_{\mathrm{dim},\mathrm{sol}}^{*}$ in Eq. (5) comprises two distinct contributions: one accounting for the dimerization process in the gas phase and another incorporating the transfer of both monomers and the resulting dimer from the gas phase to solution, represented by their respective solvation Gibbs free energies. As previously mentioned, within the framework of a thermodynamic cycle, solvation free energies can be reliably estimated using continuum solvation models. Among these, the SMD model is particularly effective due to its proven accuracy for neutral solutes. The gas-phase component, ${\Delta }_{r}{G}_{\mathrm{dim},\mathrm{gas}}^{*}\left({\mathrm{CuL}}_{2}\right)$ is highly sensitive to the level of theory employed—as well as to approximations such as the ideal gas behavior and the harmonic oscillator model—and therefore requires careful benchmarking. In this context, our study provides a comprehensive and reliable benchmarking framework to aid in selecting an appropriate DFA-based method for analyzing the dimerization of specific Cu(L) complexes. To this end, we computed the electronic energy component of ${\Delta }_{r}{G}_{\mathrm{dim},\mathrm{gas}}^{*}\left({\mathrm{CuL}}_{2}\right)$ for the model system ${\mathrm{CuL}}_{2}$ (with L = acetate) using a high-level local coupled-cluster approach, DLPNO-CCSD(T)[79–82]/aug-cc-pVTZ [83, 84] (denoted aug-cc-pVTZ/C). Based on the performance ranking of the functionals from this benchmark, we then applied the top-performing DFAs to investigate the dimerization of a Cu(II)-indomethacin complex in ethanolic solution—a system for which no experimental thermodynamic data is currently available.

All our DFT calculations were carried out using the Gaussian09 computational package [85]. DLPNO-CCSD(T) calculations were performed in Orca v.5.0 [86, 87].

## Results and discussion

### Equilibrium distances and vibrational constants

Accurate molecular geometries are a fundamental prerequisite for any reliable electronic structure-based chemical analysis. In computational studies, the quality of optimized structures critically determines the accuracy of predicted thermodynamic, spectroscopic, and electronic properties. While extensive benchmarking efforts exist for organic and organometallic systems, this work focuses specifically on the structural characterization of Cu–Cu and Cu–O bonding motifs in ${\mathrm{Cu}}_{2}^{\mathrm{q}}$ and ${\mathrm{CuO}}^{\mathrm{q}}$ species. These bonds are of particular relevance due to their occurrence in redox-active copper complexes, metalloenzymes, and potential drug candidates. The principal objective of this study is to assess the performance of various density functionals in describing these interactions, thereby identifying the most appropriate exchange–correlation functional for Cu–Cu and Cu-ligand environments. This is especially relevant in the context of CuₙL-type dimers, where L represents a bioactive or pharmacologically relevant ligand scaffold, which is binding to the metallic center by Cu–O bond(s). The goal is to identify the most reliable density functional approximations (DFAs) for accurately modeling Cu–Cu and Cu-ligand interactions in CuₙL-type dimers, where L represents a ligand of pharmacological interest. By benchmarking various functionals against these coordination environments, users can rationally select DFA candidates that are best suited to the electronic and structural characteristics of the ligand L and its coordination to copper centers.

**Fig. 2:**
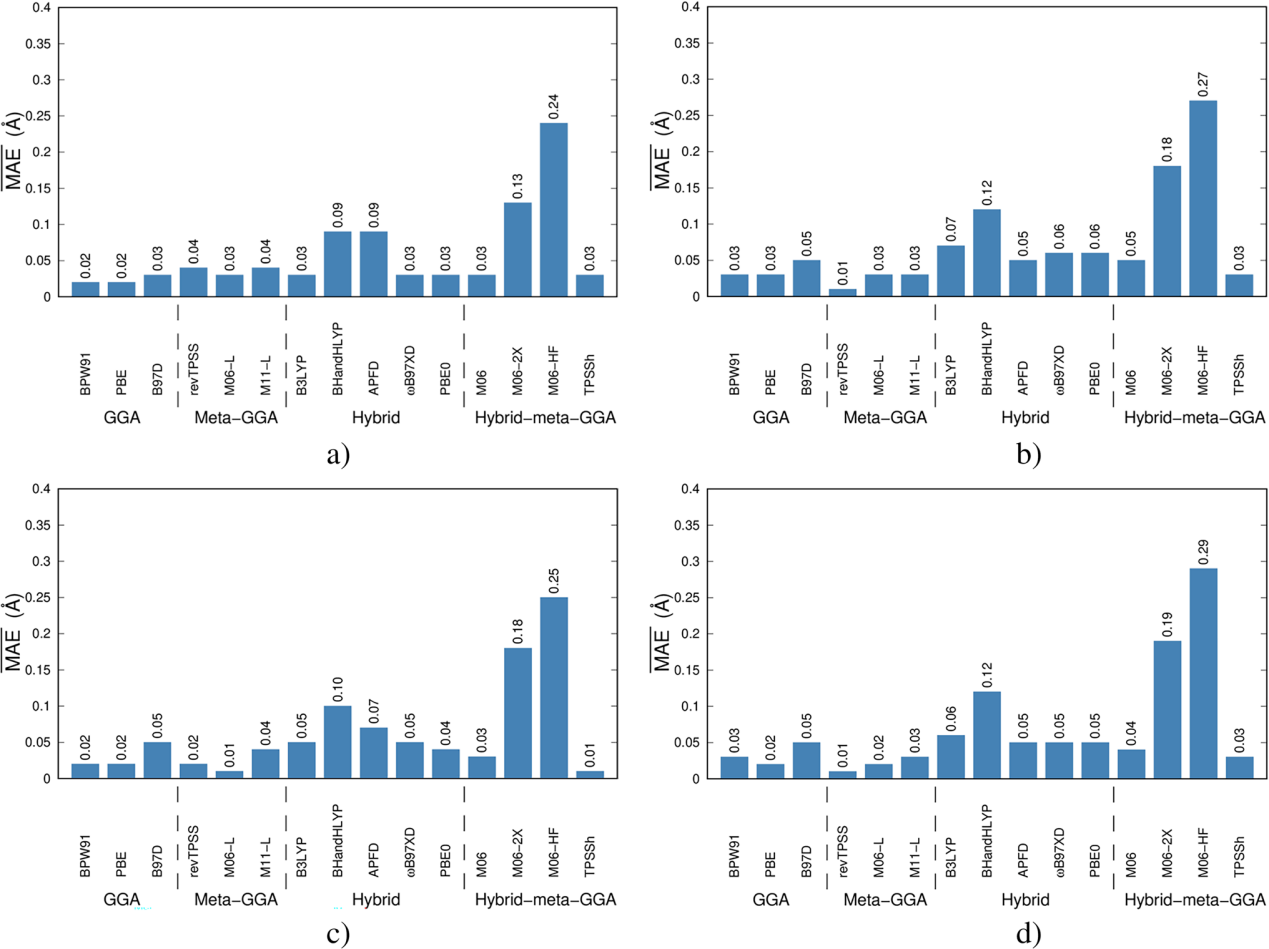
$\overline{\mathrm{MAE}}$ s for the calculated bond distances (Å) using the set of fifteen DFAs here considering for benchmarking purposes. **a**) Def2-SVP, **b**) Def2-TZVP, **c**) 6–31 + G(d,p), and **d**) 6–311 + G(d,p)

In Fig. 2, we show the $\overline{\mathrm{MAE} }$ s corresponding to the equilibrium distances of the ${\mathrm{Cu}}_{2}^{\mathrm{q}}$ and ${\mathrm{CuO}}^{\mathrm{q}}$ species. In general, the most important deviations were observed for the M06-2X and M06-HF DFAs. Conversely, GGAs and meta-GGAs exhibit values close to experimental records. This pattern is reproduced if the analysis is performed for each of the compounds under consideration (see Figures SI1-SI6). The accuracy of equilibrium geometry predictions from DFT functionals depends on how they balance exchange, correlation, and dispersion interactions. Let us break down the key differences among these Minnesota functionals: (i) M06-2X has 54% exact Hartree–Fock (HF) exchange (%X_HF_), and M06-HF has a full 100% HF exchange. This high fraction X_HF_ over-localizes electron density, making bond lengths too short and angles distorted. HF exchange lacks electron correlation, which is crucial for equilibrium structures. (ii) Dispersion is not well-captured. While M06-2X and M06-HF include some empirical dispersion-like effects, they do not handle medium- to long-range correlation as well as needed for good geometries. On the other hand, GGAs and meta-GGAs use pure DFT exchange, which is more delocalized and smoother in energy variation around equilibrium, and this leads to more realistic bond lengths.

In the next stage of this study, we focused on benchmarking a theoretical protocol for the prediction of vibrational constants. These constants, when used alongside optimized geometrical parameters, serve as powerful computational observables that can be directly compared with experimental spectroscopic data. This approach enables the accurate identification of characteristic vibrational modes associated with Cu–O and Cu–Cu bonding interactions. Furthermore, it allows for the detection of subtle spectral shifts linked to changes in oxidation state, ligand coordination, or metal–ligand bond strengths, providing critical insight into the electronic and structural dynamics of copper complexes. In addition, by integrating vibrational data with structural benchmarks, our methodology not only validates the presence of specific bonding motifs but also enhances the interpretation of experimental IR and Raman spectra. Furthermore, vibrational constants derived from a reliable theoretical protocol can serve as a foundational step toward the development of accurate force fields for molecular dynamics (MD) simulations. These force fields can then be applied to model Cu₂ and CuO-containing species across various oxidation and spin states, capturing the subtle changes in metal–metal and metal–ligand interactions that occur under different redox conditions.

**Fig. 3:**
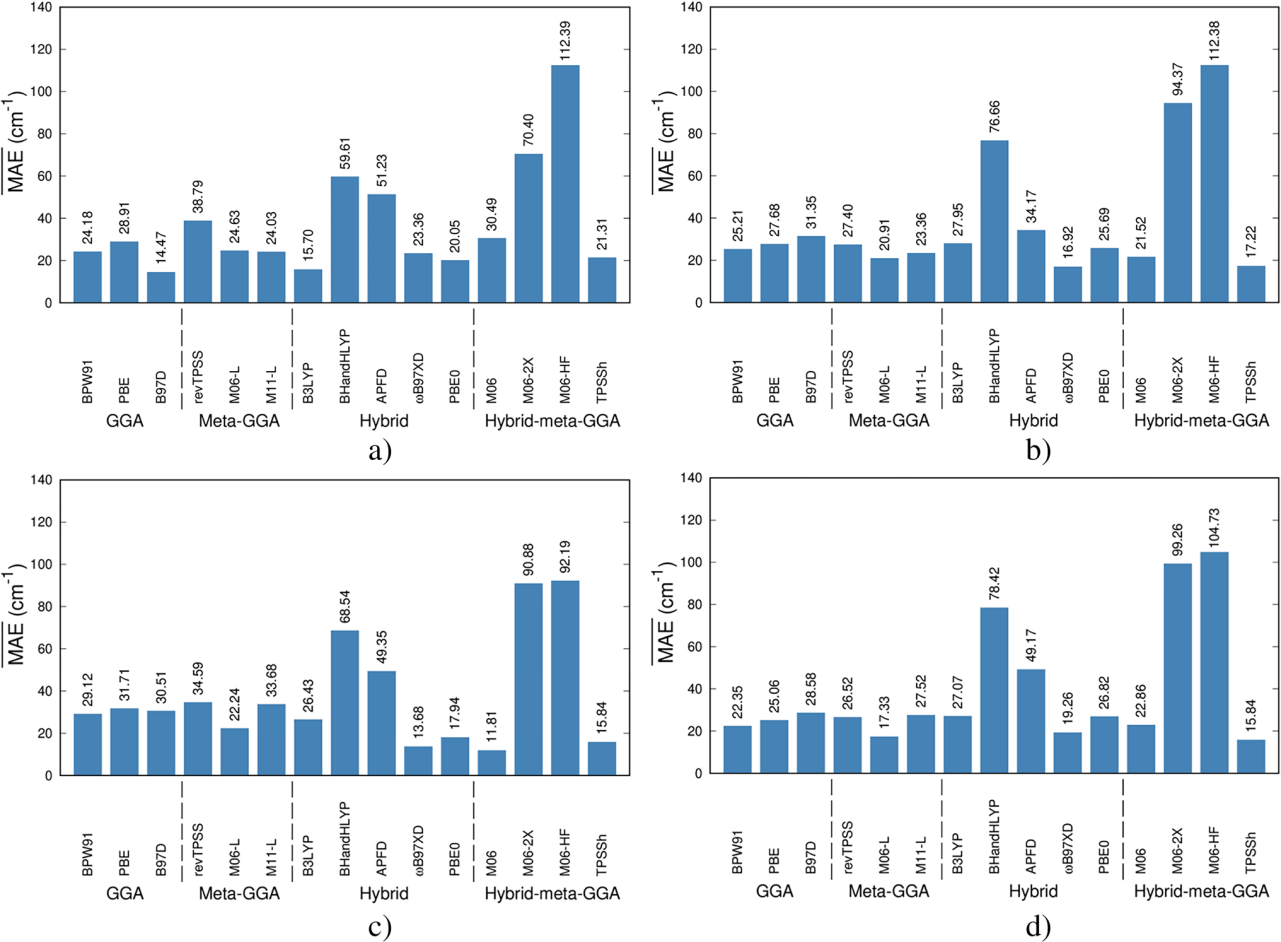
$\overline{\mathrm{MAE} }$ s for the vibrational constants (cm^−1^) using the set of fifteen DFAs here considering for benchmarking purposes. **a**) Def2-SVP, **b**) Def2-TZVP, **c**) 6–31 + G(d,P), and **d**) 6–311 + G(d,p)

In Fig. 3, we show the $\overline{\mathrm{MAE} }$ s obtained after the computation of the vibrational constants, *ω*_*e*_, corresponding to the ${\mathrm{Cu}}_{2}^{\mathrm{q}}$ and ${\mathrm{CuO}}^{\mathrm{q}}$ species with *q* = 0, + 1, –1. Individual MAEs (of each of these compounds) can be consulted in Figures SI1-SI6 of the supplementary material. Comparing the ${\omega }_{e}$ calculated with DFAs/Ahlrich’s basis sets, the $\overline{\mathrm{MAE}}\text{s }$ of BPW91, B97D, B3LYP, BHandHLYP, PBE0, and M06-2X increase for Def2-TZVP with respect to Def2-SVP, while M06-HF provides practically the same result with both basis sets. For the rest of the DFAs, the results with Def2-TZVP are more accurate than those obtained with Def2-SVP. Evidently, the $\overline{\mathrm{MAE} }$ s of BHandHLYP, M06-2X, and M06-HF allow us to conclude that high amounts of %X_HF_ affect the precision in the prediction of ${\omega }_{e}$. For DFAs/Pople’s basis sets, B3LYP, BHandHLYP, wB97XD, PBE0, M06, M06-2X, and M06-HF increase the $\overline{\mathrm{MAE}}\text{s }$ with the amount of valence orbitals. M06-2X (54% *X*_HF_) and M06-HF (100% *X*_HF_) often predict vibrational constants poorly because they overestimate bond stiffness, leading to systematically overestimated vibrational frequencies (especially stretching modes). HF exchange increases the curvature of the PES, causing larger force constants and too-high vibrational frequencies. At moderate HF exchange, 20–30% (B3LYP, M06), this improves accuracy (see Fig. 3).

Analyzing each chemical species (see Figures SI7-SI12), we can conclude that for ${\mathrm{Cu}}_{2}$, the M06, APFD, ωB97XD, revTPSS, and TPSSh/Def2-TZVP approaches are adequate for the calculation of ${\omega }_{e}$. If Pople’s basis sets are considered, the DFAs BPW91, PBE, revTPSS, M06-L, APFD, ωB97XD, M06, and TPSSh must be rather selected. In the case of ${\mathrm{Cu}}_{2}^{+}$, the DFAs APFD, M06, and TPSSh together with Def2-TZVP and Pople’s basis provided in general reliable results. For ${\mathrm{Cu}}_{2}^{+}$, $\mathrm{CuO}$, ${\mathrm{CuO}}^{+}$, and ${\mathrm{CuO}}^{-}$ compounds, the DFAs with higher *X*_HF_ were the less accurate.

In general, GGA and meta-GGA functionals offer reliable predictions for structural parameters and vibrational constants. Hybrid functionals like PBE0, B3LYP, and TPSSh (hybrid meta-GGA) also serve as strong alternatives for investigating these electronic properties. However, based on reported data, using functionals with a high percentage of *X*_HF_ is not recommended.

### Ionization energies and electron affinities of Cu_2_ and CuO

Ionization energies (IE) and electron affinities (EA) are fundamental quantities for assessing the ability of chemical species to participate in charge-transfer processes, acting as electron donors or acceptors. Even more, vertical IEs and EAs are key ingredients for the computation of chemical reactivity descriptors derived from conceptual density functional theory (CDFT) [88, 89]. These include global descriptors such as the chemical potential, chemical hardness, and the electrophilicity index [90–93], as well as their position-dependent counterparts—local chemical potential, local hardness, local electrophilicity, and other related quantities [94–98]. Collectively, these descriptors provide valuable insight into the reactivity patterns and site-specific behavior of chemical species using simple yet powerful theoretical tools [90, 91, 99]. Therefore, the development of a computational protocol capable of accurately predicting vertical IEs and EAs is essential for understanding and rationalizing the electronic reactivity behavior of Cu(II)-based complexes, including the CDFT framework.

Additionally, while adiabatic IEs and EAs are not directly used to compute reactivity descriptors, they are crucial for analyzing the intrinsic chemical properties of such complexes and they can be rather confronted to experimental measurements for processes involving lower time scale, for instance, biological processes involving redox and/or catalytic chemistry.

Moreover, accurate IEs and EAs are often required in high-accuracy methods like symmetry-adapted perturbation theory (SAPT) [100, 101], where they play a vital role in correcting the asymptotic behavior of selected density functionals—an important step for accurately describing non-covalent interactions. In this regard, having a reliable theoretical protocol that can provide precise IEs and EAs becomes especially important when studying Cu(II) complexes involved in biological environments, where weak interactions are key determinants of structure, stability, and function.

**Fig. 4:**
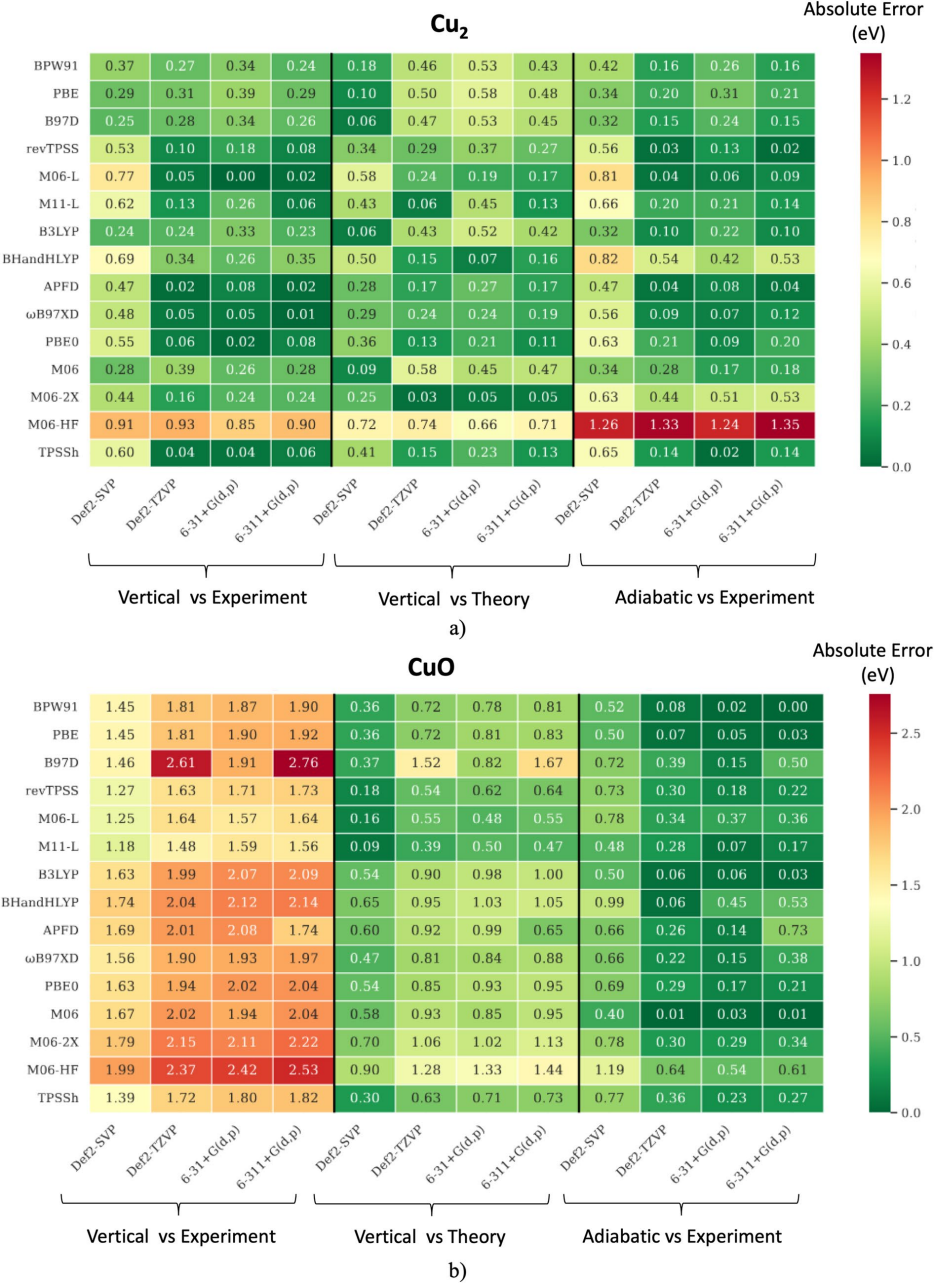
Absolute errors (in eV) in the computed vertical (V) and adiabatic (A) ionization energies (IEs) for ${\mathrm{Cu}}_{2}$ (**a**) and $\mathrm{CuO}$ (**b**), relative to both experimental and theoretical reference values. Experimental reference IEs are 7.899 ± 0.00776 eV [102] for Cu₂ and 9.41 ± 0.0177 eV for CuO [103]. Theoretical reference IEs (7.71 eV for Cu₂ and 10.50 eV for CuO) were obtained at the CCSD(T)/aug-cc-pVTZ level in a vertical fashion. Errors are reported as vertical IE vs. experiment, vertical IE vs. theory, and adiabatic IE vs. experiment

Figure 4 presents the absolute errors in ionization energy predictions for Cu₂ and CuO using various levels of theory. We begin by comparing theoretical adiabatic IE values—calculated with the Def2-TZVP basis set—to experimental results. For Cu₂, the density functional approximations (DFAs) revTPSS, M06-L, APFD, ωB97XD, and PBE0 yield predictions closest to the experimental IE (7.899 eV), as seen in Fig. 4. Notably, revTPSS, M06-L, and APFD incorporate thermochemical properties of Cu(II) species in their design. The percentage of exact Hartree–Fock exchange in hybrid DFAs plays a crucial role in IEs accuracy. Among the hybrids, APFD (23% *X*_HF_) and B3LYP (20% *X*_HF_) produce lower mean absolute errors (MAEs), while PBE0 and BHandHLYP (both 25% *X*_HF_) show significantly higher deviations. These results suggest that higher %*X*_HF_ is not beneficial for modeling Cu₂, a trend confirmed by meta-hybrid GGA functionals. Specifically, M06-HF (100% *X*_HF_) exhibits a large MAE of 0.93 eV. In the case of CuO, generalized gradient approximations (GGAs) generally yield MAEs below 2.0 eV, with the exception of B97D. Meta-GGAs further reduce the error. As with Cu₂, hybrid and meta-hybrid DFAs perform better with lower %*X*_HF_. TPSSh deviates significantly from this pattern and stands out as an outlier. Next, we compare DFA/Def2-TZVP results with high-level CCSD(T)/aug-cc-pVTZ calculations (second series of values in Fig. 4). For Cu₂, M06-HF performs the worst (MAE = 0.74 eV), whereas M06-2X and M11-L show excellent agreement with CCSD(T), both achieving MAEs below 0.06 eV. In contrast, for CuO, GGAs, hybrids, and hybrid-meta-GGAs deviate more from CCSD(T), with MAEs above 0.63 eV. Meta-GGAs—particularly M11-L (MAE = 0.39 eV)—provide results closer to the CCSD(T) benchmark. Finally, comparing adiabatic DFA predictions with experimental values (third series of values in Fig. 4), revTPSS, M06-L, and APFD again demonstrate superior accuracy for Cu₂, all achieving MAEs below 0.05 eV. M06-2X (100% *X*_HF_), by contrast, shows a large deviation of 1.33 eV. For CuO, the GGAs BPW91 and PBE deliver the most accurate predictions, with MAEs of 0.02 eV and 0.05 eV, respectively. In contrast, M06-HF proves inadequate, with an MAE of 0.54 eV. As expected, the choice of basis set plays a critical role in accurately predicting ionization energies. In general, larger basis sets are necessary to properly account for the electronic relaxation associated with electron detachment from the molecular potential. For both chemical species and across all three comparisons—vertical IE vs. experiment, vertical IE vs. theory, and adiabatic IE vs. experiment—we observed that increasing the basis set size from Def2-SVP to Def2-TZVP significantly reduced the mean absolute errors (MAEs). In contrast, the Pople basis sets (6–31 + G(d,p) and 6–311 + G(d,p)) showed a different behavior. For Cu₂, expanding the basis set by adding more orbitals did not lead to a notable improvement in accuracy across any of the three comparison cases. Overall, the most reliable results were obtained using the Def2-TZVP and 6–31 + G(d,p) basis sets, as evidenced by the lower MAEs reported in Fig. 4.

**Fig. 5:**
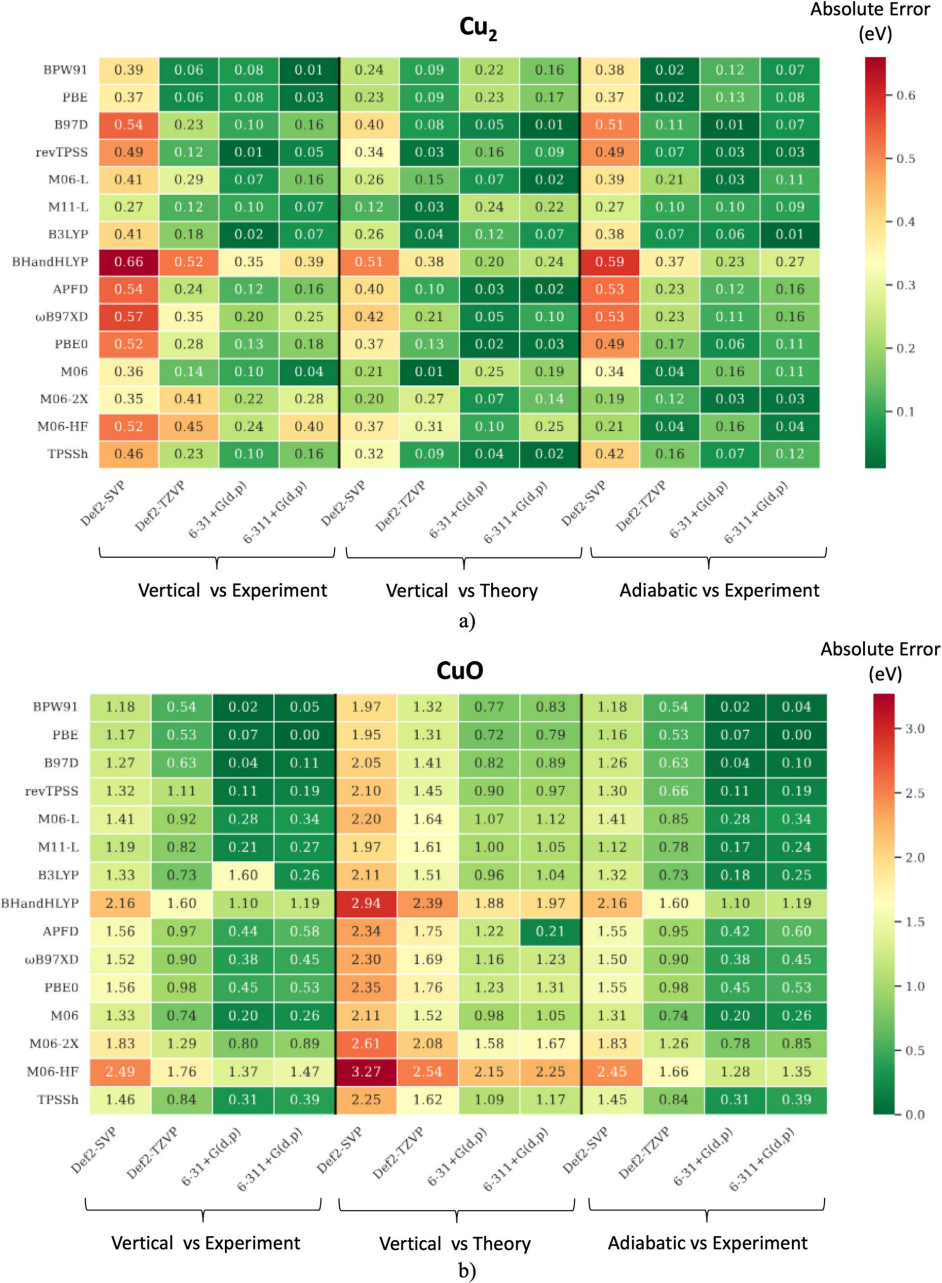
Absolute errors (in eV) in the computed vertical (V) and adiabatic (A) electron affinities (EAs) for ${\mathrm{Cu}}_{2}$ (**a**) and $\mathrm{CuO}$ (**b**), relative to both experimental and theoretical reference values. Experimental reference EAs are 0.836 ± 0.006 eV [104] for Cu₂ and 1.777 ± 0.006 eV for CuO [105]. Theoretical reference EAs (0.69 eV for Cu₂ and 2.56 eV for CuO) were obtained at the CCSD(T)/aug-cc-pVTZ level in a vertical fashion. Errors are reported as vertical EA vs. experiment unclosed, vertical EA vs. theory, and adiabatic EA vs. experiment

In Fig. 5, we reported the theoretical and experimental electron affinities of Cu₂ and CuO. The following analysis focuses on comparing experimental values with adiabatic theoretical predictions using the Def2-TZVP basis set (first series of values in Fig. 5). For Cu₂, the calculated adiabatic EAs with Def2-TZVP show that the GGAs BPW91 and PBE provide the most accurate predictions, both with MAEs = 0.06 eV, closely matching the experimental value of 0.836 eV. Among hybrid functionals, B3LYP (25% *X*_HF_) delivers the lowest MAE (0.18 eV), while the largest deviation is observed with BHandHLYP (50% *X*_HF_), yielding an MAE of 0.52 eV. In the case of hybrid-meta-GGA functionals, a clear trend emerges: increasing the percentage of exact exchange results in greater deviation with respect to experiment. TPSSh being the exception, however, the latter does not belong to the family of functionals proposed by Truhlar. For CuO, GGA functionals again outperform others DFAs. BPW91 and PBE yield the smallest deviations with respect to experiment, with MAEs of 0.54 eV and 0.53 eV, respectively. In contrast, BHandHLYP (1.60 eV) and M06-HF (1.76 eV) show the poorest performance, reaffirming the negative impact of high exact exchange on EA predictions. When comparing DFA/Def2-TZVP results with CCSD(T)/aug-cc-pVTZ values (second series of values in Fig. 5) for Cu₂, strong agreement is observed across the full range of Perdew’s “Jacob’s ladder” of functionals. Specifically, all three GGAs yield MAEs below 0.1 eV. Among meta-GGAs, revTPSS and M11-L both perform exceptionally well, each with an MAE of 0.03 eV. B3LYP again stands out among hybrid functionals (MAE = 0.04 eV), while M06, a hybrid-meta-GGA, achieves the best overall agreement with CCSD(T), showing an MAE of just 0.01 eV. Comparing DFA/Def2-TZVP results directly with experimental data for Cu₂, BPW91 and PBE offer near-perfect agreement (MAE = 0.02 eV). These are closely followed by M06 (0.04 eV), B3LYP (0.07 eV), and revTPSS (0.07 eV). For CuO, GGA functionals again dominate in accuracy, while BHandHLYP and M06-HF yield the least reliable predictions. As with ionization energies, high fractions of exact exchange (%*X*_HF_) consistently lead to larger deviations from both experimental and CCSD(T) values for both Cu₂ and CuO.

Increasing the size of the Ahlrich basis sets has a positive impact on the quality of the results for both species. On the other hand, increasing the number of orbitals considered in the Pople basis sets does not have a significant impact on the quality of the results obtained for Cu_2_ and CuO species, especially if the B97D, M06-L, BHandHLYP, APFD, ωB97XD, PBE0, and TPSSh approaches are considered. Nevertheless, our results indicate that the 6–31 + G(d,p) basis set showed the best overall performance for both copper compounds.

The benchmarking of Cu₂ and CuO species highlights consistent trends across ionization energies (IEs), electron affinities (EAs), and vibrational constants (*ωₑ*). GGAs and meta-GGAs, such as PBE, BPW91, revTPSS, M06-L, and APFD, generally provide the most reliable predictions, achieving errors below 0.2 eV for IEs and EAs and yielding accurate vibrational constants. In contrast, functionals with high Hartree–Fock exchange (M06-2X, M06-HF, BHandHLYP) systematically fail: they overestimate bond stiffness, distort vibrational frequencies, and deviate significantly from both experimental and CCSD(T) reference data. Hybrids with moderate exchange (20–30%, e.g., B3LYP, TPSSh, M06) strike the best balance, reproducing structural and electronic properties with good consistency. Basis set choice also plays a key role: Def2-TZVP systematically improves accuracy, while larger Pople sets offer no clear advantage. Taken together, the results indicate that low-to-moderate exchange DFAs, particularly GGA, meta-GGA, and carefully selected hybrids, are the most suitable for describing the electronic structure and vibrational behavior of Cu-based dimers and oxides, while high-%*X*_HF_ functionals should be avoided due to their systematic bias.

### Dissociation energies of ${\mathrm{CuO}}^{\mathrm{q}}$ species

This study constitutes a foundational step toward the accurate theoretical characterization of copper-based dimerization and complexation processes. Specifically, we examine the bond dissociation behavior of ${\mathrm{Cu}}_{2}^{\mathrm{q}}$ and ${\mathrm{CuO}}^{\mathrm{q}}$ species across multiple charge states (*q* = 0, ± 1). The inclusion of both neutral and charged species serves two key purposes: first, to reflect the fact that Cu(I) and Cu(II) commonly exist in ionized forms under physiological and solution-phase conditions; and second, to extend the benchmarking framework to encompass redox-active processes, which are central to the chemistry of copper in both biological and catalytic contexts. The complete set of chemical equilibria considered in this analysis is presented in Eqs. (6)–(11).

6 $${\mathrm{Cu}}_{2} \to 2\text{ Cu}$$

7 $${\mathrm{Cu}}_{2}^{+} \to {\mathrm{Cu}}^{+}+\mathrm{Cu}$$

8 $${\mathrm{Cu}}_{2}^{-} \to {\mathrm{Cu}}^{-}+\mathrm{Cu}$$

9 $$\text{CuO }\to \mathrm{Cu}+\mathrm{O}$$

10 $${\mathrm{CuO}}^{+} \to {\mathrm{Cu}}^{+}+\mathrm{O}$$

11 $${\mathrm{CuO}}^{-} \to {\mathrm{Cu}}^{-}+\mathrm{O}$$

**Fig. 6:**
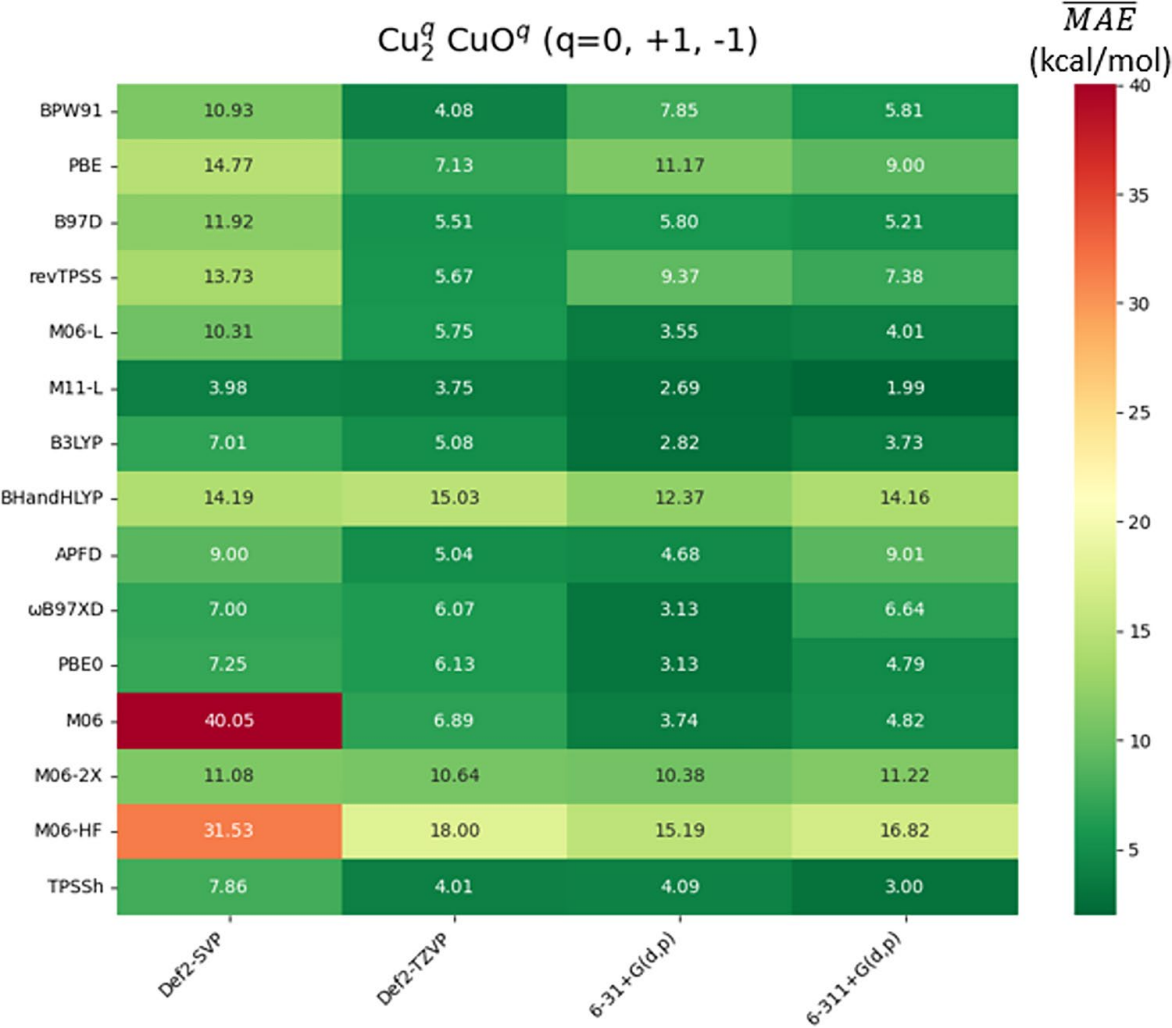
$\overline{\mathrm{MAE} }$ of De with respect to the experimental values of the ${\mathrm{Cu}}_{2}^{\mathrm{q}}$, ${\mathrm{CuO}}^{\mathrm{q}}$ (*q* = 0, + 1, −1) species. All data in kcal/mol

The complete set of recorded $\overline{\mathrm{MAE}}\text{s }$ are available in Tables SI1-SI6 of the supporting information. The global results are reported in Fig. 6. From the Ahlrich’s basis sets, Def2-TZVP exhibited the best performance, except when it is conjointly used with the BHandHLYP functional. For Pople’s basis sets, the $\overline{\mathrm{MAE}}\text{s }$ of M06-L, B3LYP, BHandHLYP, APFD, ωB97XD, PBE0, M06, M06-2X, and M06-HF increase going from 6–31 + G(d,p) to 6–311 + G(d,p). In most of the DFAs here tested, the deviations for ${\mathrm{CuO}}^{\mathrm{n}}$ (*n* = 0, + 1, − 1) are larger than those for ${\mathrm{Cu}}_{2}$ (see Tables SI4-SI6). For ${\mathrm{CuO}}^{\mathrm{q}}$ (*q* = 0, + 1, − 1), similar deviations were reported using theoretical methods constituted by post Hartree–Fock approaches; for instance, MR-ACPF/CBS deviates 7.58 kcal/mol from the experimental data, while CCSD(T)/CBS does by 5.81 kcal/mol [106]. The DFAs that present the highest $\overline{\mathrm{MAE} }$ are BHandHLYP, M06-2X, and M06-HF. As in the case of IE, EA, *ω*_*e*_, and *r*_*e*_, the greater %*X*_HF_, the larger deviations are found, consistent with previous studies neutral metal dimers [107]. The analysis of IE, EA, *ω*_*e*_, and *D*_*e*_ allows us to conclude that the DFAs M06-2X and M06-HF are not suitable choices for the study of Cu(II) systems, so we omit these DFAs in the remainder of our analysis.

### Exchange coupling of the Cu_2_AcO_4_ complex

Benchmarking exchange coupling constants (*J* values) in dinuclear copper complexes is particularly appropriate in a biologically relevant context, as many essential metalloenzymes and metalloproteins contain closely spaced Cu(II) centers that exhibit magnetic interactions. Examples include tyrosinase, catechol oxidase, and hemocyanin, where antiferromagnetic or ferromagnetic coupling between Cu ions plays a crucial role in oxygen activation, electron transfer, and substrate binding. Accurately modeling these exchange interactions is fundamental to understanding spin-state energetics, redox behavior, and structure–function relationships in copper-based biological systems.

The Cu₂(OAc)₄ complex was selected as a prototypical model of a magnetically coupled dicopper(II) system, primarily due to the presence of Cu–O bonds, which are central to the present study. In addition to its relevance as a minimal model for exchange-coupled Cu(II) centers—akin to those found in metalloenzymes such as tyrosinase and hemocyanin—the complex features acetate bridging ligands, whose structural motif is highly relevant in both pharmaceutical and biological contexts. Notably, the acetate group is a key functional component in widely used drugs such as indomethacin and aspirin, and its carboxylate moiety is frequently encountered in drug candidates and biological assemblies, where it plays critical roles in metal coordination, enzyme inhibition, and molecular recognition. Therefore, Cu₂(OAc)₄ provides a chemically and biologically meaningful scaffold for benchmarking density functionals in systems that combine magnetic exchange, metal–ligand interactions, and biorelevant ligands. We estimated the coupling constant (*J*) for *S*_1_ ≥ *S*_2_, accordingly to the following equation,

12 $$J=\frac{{E}_{\mathrm{BS}}-{E}_{\mathrm{HS}}}{2{S}_{1}{S}_{2}-{S}_{2}}$$

where *E*_BS_ and *E*_HS_ are the broken symmetry and high spin energies, respectively. When *S*_1_ = *S*_2_ = 1/2 as in our case, we can rewrite the equation as follows:

13 $$J={E}_{\mathrm{BS}}-{E}_{\mathrm{HS}}$$

Table 1 depicts the < *S*_BS_^2^ > and *J* values evaluated with the thirteen DFAs under consideration conjointly with the 6–31 + G(d,p) basis. We exclusively selected this basis set because according to the $\overline{\mathrm{MAE} }$ s estimated for the IEs, Eas, and Des; 6–31 + G(d,p) is a suitable alternative to describe electronic properties of Cu_2_ and CuO accurately and at moderate computational cost. Also, 6–31 + G(d,p) basis set has been shown to give reasonably accurate coupling constants in many systems, especially in Cu-based systems [108]. ωB97XD, PBE0, M11-L, and B3LYP show values closer to < *S*_BS_^2^ >, while the GGAs are farther away. Datta et al. reported < *S*_BS_^2^ > with BP86, obtaining values 0.93 cm^−1^ and 0.89 cm^−1^ with the 6-31G* and 6–311 + + G** basis sets, respectively [109]. It is clear that the GGAs do not show a good performance in the prediction of < *S*_BS_^2^ > of ${\mathrm{Cu}}_{2}{\mathrm{AcO}}_{4}$. On the other hand, the broken symmetry solution for BHandHLYP, APFD, M06, and TPSSh converges to the closed shell singlet state. The shortcoming of the BS approach has been reported by Neese, so it is not unusual for the BS solution to converge to the standard closed shell determinant. According to Neese, it is not easy to determine when this situation will occur, due to the strong non-linearity of the SCF equations [67].

**Table 1:** < *S*_BS_^2^ > and *J* of ${\mathrm{Cu}}_{2}{\mathrm{AcO}}_{4}$. Experimental value of *J*: 286 cm^−1^ [110]. All values in cm^−1^.

Type DFA	DFA	< *S*_BS_^2^ >	*J*
**GGA**	BPW91	0.86	606
	PBE	0.86	606
	B97D	0.83	645
**Meta-GGA**	revTPSS	0.90	547
	M06-L	0.94	437
	M11-L	0.98	287
**Hybrid**	B3LYP	0.98	276
	BHandHLYP	0	-
	APFD	0	-
	ωB97XD	0.99	205
	PBE0	0.99	231
**Hybrid meta-GGA**	M06	0	-
	TPSSh	0	-

It can be observed that the functional with the best performance was M11-L. According to the authors, this DFA notably improves multiple chemical properties, largely because it compensates for the self-interaction error (SIE). According to Truhlar, M11-L has a better SIE improvement than any other DFA, that statement is supported when we compare the J calculated with M11-L and ωB97XD, since the latter DFA was also designed to reduce SIE. The performance of B3LYP is surprisingly modest, considering that it was mainly parameterized for reproducing thermochemistry data of organic molecules. Nonetheless, the hybrid DFAs B3LYP and PBE0 suggest that including a high percentage of *X*_HF_ is not the best strategy for the computation of these quantities.

### Dimerization Gibbs free energy of reaction for Cu(II)/acetate and Cu(II)/Indo complexes

As the second component of this study, we assessed the performance of the selected density functional approximations (DFAs) in predicting chemical equilibria in the solution phase. Specifically, we modeled solvation effects using a polarizable continuum model (PCM) with ethanol as the dielectric medium, following the thermodynamic cycle illustrated in Fig. 1. Ethanol was chosen as the solvent due to its intermediate polarity and its widespread use in pharmaceutical research, where many drug-like compounds exhibit limited solubility in water due to their lipophilic nature. As such, experimental investigations involving these compounds are frequently conducted in solvents such as ethanol or dimethyl sulfoxide (DMSO).

As previously discussed, the copper-acetate complex serves as a prototypical model for biologically relevant systems, including several drug molecules and protein–ligand assemblies, where carboxylate-containing ligands interact with transition metal centers. In such complexes, coordination typically occurs through the carboxylate moiety, with both oxygen atoms binding to the Cu(II) ion [111]. This bidentate interaction helps stabilize the + 2 oxidation state, maintain charge neutrality, and promote the formation of the square-planar geometry commonly favored by Cu(II) due to its d⁹ electronic configuration and Jahn–Teller effects. The propensity of Cu(II)-acetate units to undergo dimerization arises from their partially accessible coordination sites. It is proposed that dimer formation occurs via interaction between the uncoordinated axial positions of two monomeric units, resulting in a bridging carboxylate network and the emergence of a paddlewheel-type structure.

As mentioned, the computation of solution-phase equilibrium constants via thermodynamic cycles requires the integration of two distinct types of calculations: (i) gas-phase thermochemical data, which provides the fundamental energetic contributions, and (ii) solvation free energies, which account for the influence of the solvent environment. In this study, we first focused on benchmarking the gas-phase component, as it constitutes the dominant contribution to the overall Gibbs free energy in solution. Once the reliability of gas-phase results was established, solvation effects were incorporated using the SMD implicit solvation model, a widely adopted and well-validated continuum approach that captures both electrostatic and non-electrostatic interactions with the solvent.

### Electronic energy contribution to the dimerization energy of Cu(II)/acetate species

The electronic structure of transition metal complexes may not be accurately described using monodeterminant theories, such as DFT. To evaluate the multireference nature of our systems, we computed the T1 test of the species involved in the *Cu(II)/acetate* dimerization equilibrium (see Fig. 1). For ${\mathrm{CuAcO}}_{2}$ and ${\mathrm{Cu}}_{2}{\mathrm{AcO}}_{4}$, the T1 test provided the 0.021 and 0.020 values, respectively, which are below the threshold established in the literature (about 0.05) [112].

**Fig. 7:**
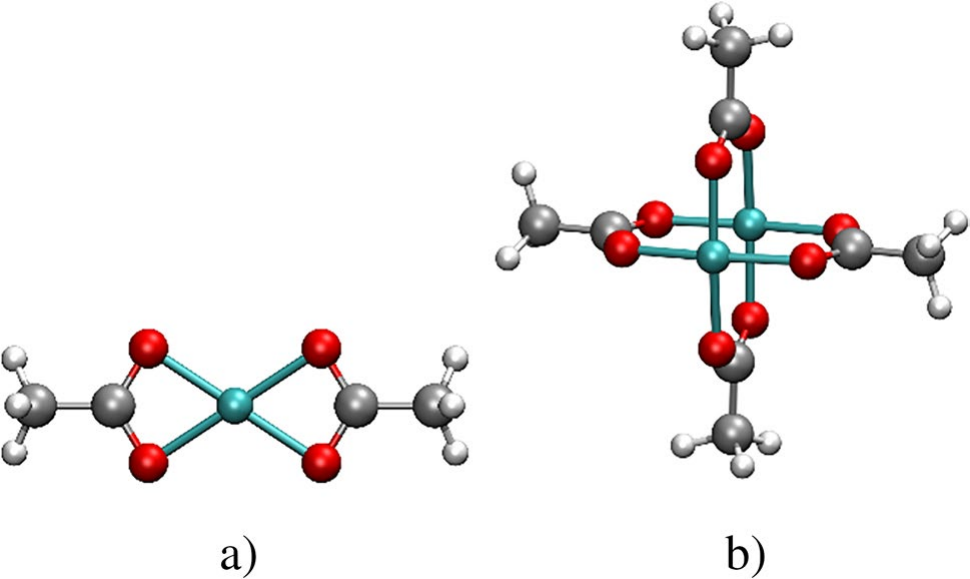
Molecular structures of **a**) ${\mathrm{CuAcO}}_{2}$ and **b**) ${\mathrm{Cu}}_{2}{\mathrm{AcO}}_{4}$ species. White spheres: hydrogen atoms; gray spheres: carbon atoms; red spheres: oxygen atoms and cyan spheres: copper atoms

The molecular geometries of the species of the *Cu(II)/acetate* complexes at both stoichiometries are shown in Fig. 7. In the mononuclear complex (${\mathrm{CuAcO}}_{2}$), the Cu(II) atom coordinates with acetate ligands in a bidentate square planar fashion (Fig. 7a). In dinuclear ${\mathrm{Cu}}_{2}{\mathrm{AcO}}_{4}$, the acetate ligands coordinate with Cu(II) in a paddle wheel-like pattern, where each metal center has a square pyramidal coordination mode (Fig. 7b).

As benchmarking study, we compared the electronic energy variation corresponding to the following chemical reaction,

14 $$2{\mathrm{CuAcO}}_{2}\to {\mathrm{Cu}}_{2}{\mathrm{AcO}}_{4}$$

obtained with the thirteen aforementioned DFAs, with the corresponding energy obtained through the DLPNO-CCSD(T)) approach. The DFAs B97D, M06-L, M11-L, ωB97XD, and M06 are in close agreement with our DLPNO-CCSD(T) reference when Pople’s basis sets are used, while the B3LYP/Def2-SVP and M06/Def2-TZVP protocols displayed the best performance for Alrich’s basis sets (Table 2). Our results indicate that *X*_HF_ may not play a determining role in the formation of the ${\mathrm{Cu}}_{2}{\mathrm{AcO}}_{4}$ species starting from its monomer. However, it is important to recall that the DLPNO-CCSD(T) method provides non-systematic deviations in reaction energies for coordination compounds. For example, for the group 12-metals (Zn, Cd, Hg), the errors are less than 2.0 kcal/mol, while for the Zr reactions, the deviations are greater than 14.4 kcal/mol [113].

**Table 2:** Electronic energy deviation from DLPNO-CCSD(T)/aug-cc-pVTZ(aug-cc-pVTZ/C). ${\Delta }_{r}{E}_{\mathrm{electronic}}^{\mathrm{DLPNO}-\mathrm{CCSD}(\mathrm{T})}-{\Delta }_{r}{E}_{\mathrm{electronic}}^{\mathrm{DFT}}$ for the dimerization reaction in Eq. (14). Results in kcal/mol

Type DFA	DFA	Def2-SVP	Def2-TZVP	6–31 + G(d,p)	6–311 + G(d,p)
**GGA**	BPW91	− 3.7	− 18.4	− 15.6	− 15.9
	PBE	3.2	− 12.2	− 9.6	− 9.9
	B97D	10.0	− 3.6	− 0.7	− 1.5
**Meta-GGA**	revTPSS	6.8	− 9.1	− 5.6	− 5.7
	M06-L	11.2	− 3.8	− 0.7	− 0.4
	M11-L	14.2	− 7.5	2.3	− 0.7
**Hybrid**	B3LYP	1.4	− 12.7	− 11.1	− 10.6
	APFD	21.3	5.5	8.9	9.4
	ωB97XD	11.3	− 3.5	− 0.5	0.2
	PBE0	5.2	− 10.1	− 7.1	− 6.3
**Hybrid meta-GGA**	M06	14.0	− 1.2	2.4	3.1
	TPSSh	3.9	− 11.3	− 8.2	− 8.0

### Dimerization Gibbs free energy of reaction in gas and solution phases of Cu(II)/acetate species

The theoretical results of the ${\Delta }_{r}{G}_{\mathrm{dim}}^{*}$ for the ${\mathrm{Cu}}_{2}{\mathrm{AcO}}_{4}$ species in gas and solution phases (ethanol) are reported in Table 3. The experimental ${\Delta }_{r}{G}_{\mathrm{dim},\mathrm{sol}}^{*}$ in ethanolic solution has been previously reported to be equal to − 5.59 kcal/mol [114]. For the Ahlrich’s and Pople’s basis sets, the solvent contribution to the dimerization energies of the Cu(II)/acetate complexes is less than 6 kcal/mol, except for M11-L and APFD. When using the BPW91/Def2-TZVP methodology, the dimerization process is not carried out in either of the two phases, contradicting the experimental information. According to the reference value, the poorest performance in both phases is obtained with the Def2-SVP basis function, a trend that was expected (see the MAD in Table SI7). The DFAs showing good performance were PBE, PBE0, TPSSh, and B3LYP in combination with both the Def2-TZVP, 6–31 + G(d,p), and 6–311 + G(d,p) basis sets.

**Table 3:** The ${\Delta }_{r}{G}_{\mathrm{dim},\mathrm{gas}}^{*}$ and ${\Delta }_{r}{G}_{\mathrm{dim},\mathrm{sol}}^{*}$ are reported in kcal/mol. Experimental value − 5.59 kcal/mol

Type DFA	DFA	Def2-SVP		Def2-TZVP		6–31 + G(d,p)		6–311 + G(d,p)	
		**Gas**	**Sol**	**Gas**	**Sol**	**Gas**	**Sol**	**Gas**	**Sol**
**GGA**	BPW91	− 15.7	− 14.7	1.5	1.9	− 2.5	− 2.3	− 3.2	− 2.4
	PBE	− 22.3	− 21.7	− 6.1	− 6.5	− 8.7	− 8.7	− 8.2	− 7.6
	B97D	− 29.6	− 26.3	− 14.3	− 15.0	− 17.0	− 16.2	− 17.7	− 14.8
**Meta-GGA**	revTPSS	− 24.4	− 20.6	− 8.4	− 6.0	− 12.9	− 10.9	− 12.4	− 10.9
	M06-L	− 31.7	− 29.7	− 16.5	− 18.0	− 18.2	− 17.7	− 18.8	− 18.3
	M11-L	− 30.8	− 21.1	− 7.9	− 1.8	− 14.3	− 4.4	− 15.6	− 7.9
**Hybrid**	B3LYP	− 19.1	− 18.7	− 6.0	− 5.2	− 7.5	− 6.6	− 7.6	− 4.9
	BHandHLYP	− 26.1	− 21.4	− 11.2	− 8.7	− 13.9	− 11.8	− 14.9	− 10.8
	APFD	− 36.6	− 28.3	− 22.3	− 15.7	− 25.9	− 17.3	− 26.5	− 18.4
	ωB97XD	− 30.8	− 27.2	− 14.6	− 15.2	− 18.4	− 18.1	− 20.8	− 16.7
	PBE0	− 24.0	− 21.8	− 9.7	− 9.5	− 11.7	− 10.8	− 13.3	− 11.8
**Hybrid meta-GGA**	M06	− 33.8	− 31.8	− 17.4	− 18.7	− 20.2	− 19.2	− 23.2	− 22.2
	TPSSh	− 22.0	− 20.1	− 6.8	− 6.6	− 9.8	− 6.8	− 9.8	− 7.4

### Copper indomethacin dimerization energy

Previously, we analyzed the ionization energies and electron affinities of the Cu₂ and CuO species. However, the inclusion of ligands (indomethacin) is essential, as they strongly influence the IE and EA of the metal center. Therefore, as part of a preliminary analysis, we have calculated these electronic properties. For this analysis, we selected the PBE, PBE0, B3LYP, and TPSSh functionals in combination with the 6–31 + G(d,p) basis set, since these methodologies showed the best performance in reproducing the dimerization energy of the Cu(II)/acetate model. In addition, we incorporated the M06L DFA because, according to reported data for CuO and Cu_2_, it provides an accurate description of the electronic properties of these benchmark species. Among the tested methods (Table 4), PBE exhibits the best overall performance, with an IE deviation of only 0.62 eV and an EA deviation of 0.12 eV, clearly outperforming the other functionals. The hybrid functional PBE0 also provides a balanced description, though with slightly larger errors for both properties (0.70 eV for IE and 0.20 eV for EA). B3LYP and TPSSh tend to underestimate the ionization energy more severely (errors of 1.02 and 1.55 eV, respectively), while their EA predictions remain moderately close to the reference. By contrast, M06L shows the largest discrepancies, underestimating the ionization energy by 1.83 eV and overestimating the electron affinity by 0.59 eV.

**Table 4:** Data at CCSD(T)/aug-cc-pVTZ (aug-cc-pVTZ/C) level: IE = 9.04 eV; EA = 1.33 eV. In parentheses the absolute errors with respect to CCSD(T)/aug-cc-pVTZ (aug-cc-pVTZ/C). For the values obtained with the DFAs, we used the basis set 6–31 + G(d,p). All values in eV

DFA	Ionization energy	Electron affinity
PBE	8.42 (0.62)	1.21 (0.12)
PBE0	8.34 (0.70)	1.53 (0.20)
B3LYP	8.02 (1.02)	1.84 (0.51)
TPSSh	7.49 (1.55)	1.72 (0.39)
M06L	7.21 (1.83)	1.92 (0.59)

**Fig. 8:**
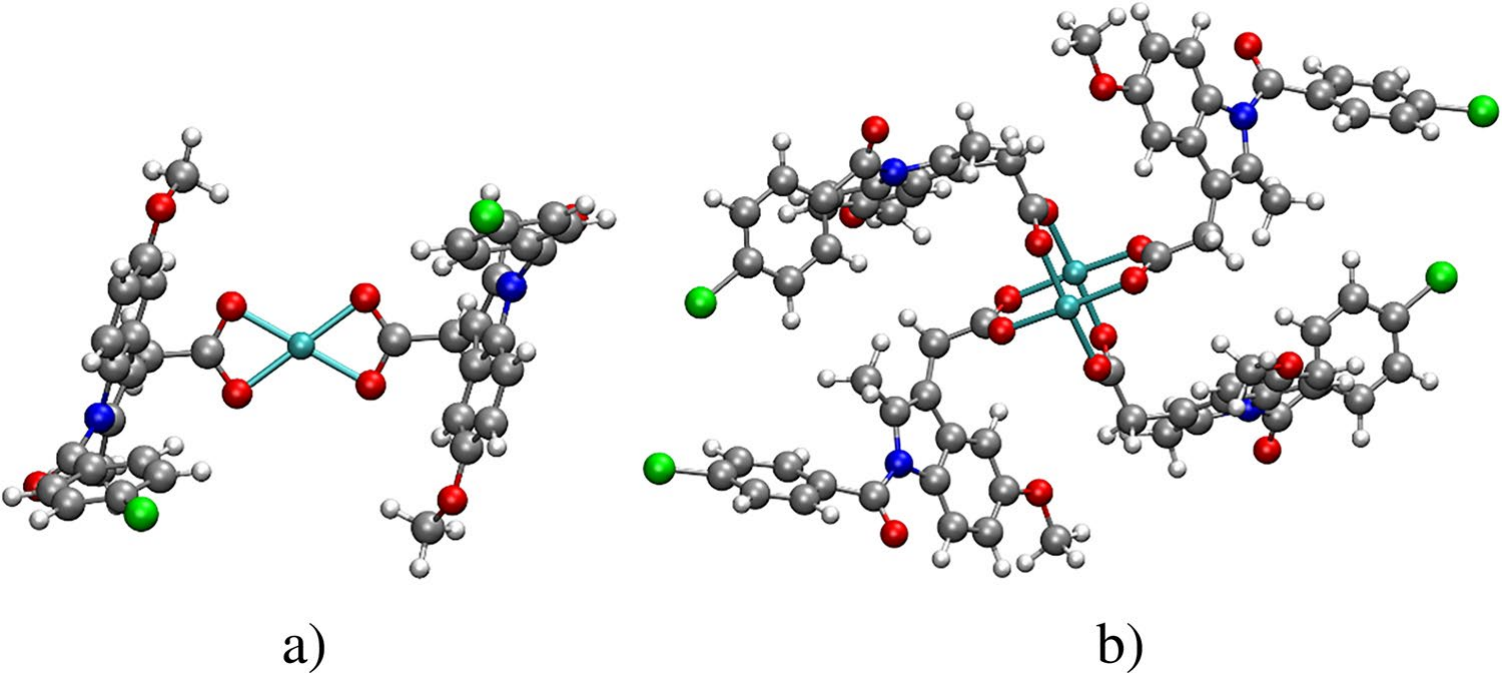
Molecular structures of **a**) ${\mathrm{CuIndo}}_{2}$ and **b**) ${\mathrm{Cu}}_{2}{\mathrm{Indo}}_{4}$. White spheres: hydrogen atoms; gray spheres: carbon atoms; blue spheres: nitrogen atoms; red spheres: oxygen atoms; green spheres: chlorine atoms and cyan spheres: copper atoms

The ${\Delta }_{r}{G}_{\mathrm{dim},\mathrm{sol}}^{*}$ quantity corresponding to the Cu(II)/Indo complexes was calculated using Eq. (5). The ${\mathrm{CuIndo}}_{2}$ mononuclear complex shows a bidentate coordination pattern (see Fig. 8a). It has been previously shown that the preferred orientation of the indomethacin ligands in the mononuclear species is the one shown in Fig. 8a. The input geometry of the ${\mathrm{Cu}}_{2}{\mathrm{Indo}}_{4}$ compound was extracted from the experimental X-ray structure [115] and subsequently optimized with the DFAs PBE, PBE0, TPSSh, and B3LYP, using the 6–31 + G(d,p) basis set.

Calculated dimerization energies of Cu(II)/Indo species in ethanol solution with the selected DFAs and basis set are reported in Table 5. There is no clear relationship between the %*X*_HF_ and ${\Delta }_{r}{G}_{\mathrm{dim},\mathrm{sol}}^{*}$. The data suggest that the process described in Eq. (5) is spontaneous, indicating that the dinuclear complex (${\mathrm{Cu}}_{2}{\mathrm{Indo}}_{4}$) is more stable than the mononuclear species (${\mathrm{CuIndo}}_{2}$).

**Table 5:** Cu(II)/Indo dimerization Gibbs reaction energy (kcal/mol) in ethanolic solution. Data obtained with 6–31 + G(d,p) basis set

**DFA**	${\Delta }_{r}{G}_{\mathrm{dim},\mathrm{sol}}^{*}$
PBE	− 5.42
PBE0	− 5.83
B3LYP	− 1.30
TPSSh	− 4.03

For this last species, values for ${\Delta }_{r}{G}_{\mathrm{dim},\mathrm{sol}}^{*}$ of − 36.42 kcal/mol have been proposed in the literature using theoretical protocols [116]. It is worth mentioning that they show a deviation of 17.25 kcal/mol with respect to the experimental data (− 19.17 kcal/mol). We believe that such a discrepancy is caused by the level of DFA used in that study, since the authors use BHandHLYP. According to the information presented in this work, this DFA is not the best option for analyzing chemistry of copper dimer complexes. According to PBE, B3LYP, and TPSSh, the ${\mathrm{Cu}}_{2}{\mathrm{Indo}}_{4}$ complex is less stable than its acetate analogue, while PBE0 predicts the same value for the ${\mathrm{Cu}}_{2}{\mathrm{Indo}}_{4}$ and ${{\mathrm{Cu}}_{2}\mathrm{AcO}}_{4}$ dinuclear species.

The application to the Cu(II)/indomethacin (Cu–Indo) complex was presented more briefly than the benchmarking on Cu_2_ and CuO, as the main focus of this work was to establish the accuracy of different DFAs in describing fundamental copper species. Nevertheless, the stability of Cu–Indo dimers has important implications that merit further consideration. (i) Coordination of indomethacin to Cu(II) can alter the drug’s physicochemical properties, potentially enhancing solubility or modifying lipophilicity. Such changes may influence bioavailability and distribution; (ii) copper coordination can affect the redox behavior of the complex, introducing new pathways for reactive oxygen species (ROS) formation or scavenging, both of which are relevant in inflammation and oxidative stress; (iii) dimer stability provides a measure of how strongly indomethacin ligates Cu(II) under physiological conditions, which could modulate the release or activation of the drug; (iv) stable Cu–Indo dimers may interact differently with biomolecular targets (e.g., cyclooxygenase enzymes), offering alternative mechanisms of pharmacological action; (v) understanding the energetic landscape of Cu–Indo dimers helps clarify whether such complexes are likely to persist in vivo or primarily act as transient intermediates. Finally, expanding the discussion of Cu–Indo stability connects the benchmarking of DFAs to real pharmacological scenarios, enhancing the biological relevance of the study and broadening its impact beyond purely computational benchmarking.

## Conclusions

Based on our theoretical analysis of ionization energies (IE), electron affinities (EA), vibrational frequencies (*ωₑ*), bond lengths (*rₑ*), and dissociation energies (*Dₑ*) for ${\mathrm{Cu}}^{\mathrm{q}}$, ${\mathrm{Cu}}_{2}^{\mathrm{q}}$, and ${\mathrm{CuO}}^{\mathrm{q}}$ (*q* = 0, + 1, –1) species, we find that expanding the valence space in Ahlrichs-type basis sets significantly improves computational accuracy. Interestingly, this improvement is not observed with Pople basis sets, where even the smaller versions yield results comparable to their larger counterparts, suggesting greater consistency for biological modeling using Pople’s framework.

In the biologically inspired dinuclear complex [Cu₂(AcO)₄], which serves as a structural mimic of Cu(II)-binding sites in metalloproteins, the singlet ground state was accurately described using the hybrid DFAs B3LYP, ωB97XD, and PBE0, along with the meta-GGA functional M11-L. The calculated < *S*^2^ > values were close to 1.00 cm^−1^, indicating correct spin-state characterization, essential for modeling redox-active Cu(II) centers in enzymes. Notably, only B3LYP and M11-L yielded exchange coupling constants (J) in excellent agreement with experimental data, with M11-L standing out due to its effective mitigation of self-interaction error (SIE), a common challenge in open-shell systems like those found in metalloproteins.

While most DFAs provided satisfactory agreement with experimental properties for the simpler Cu₂ and CuO molecules, their performance was less consistent when applied to the dimerization energetics of the Cu(II)/acetate system—a model for biologically relevant copper-carboxylate interactions. The computed dimerization energies in solution varied widely, from + 1.9 to − 22.2 kcal/mol (excluding results from the least accurate Def2-SVP basis set). Among the tested functionals, PBE, M11-L, B3LYP, and TPSSh showed the best agreement with experimental thermodynamic data, each deviating by less than 4 kcal/mol. Notably, Pople basis sets outperformed Ahlrichs-type basis sets in reproducing the dimerization energy, reinforcing their utility for modeling metal–ligand binding in bioinorganic contexts.

Building on these validated methods, we assessed the thermodynamic stability of the dinuclear [Cu₂(Indo)₄] complex, where indomethacin—a clinically relevant nonsteroidal anti-inflammatory drug (NSAID)—acts as a coordinating ligand. This system is of pharmacological interest due to the potential of metal-NSAID complexes as therapeutic agents. Gibbs energies of dimerization computed using the most reliable DFAs (PBE, PBE0, TPSSh, and B3LYP) ranged from − 5.42 to − 1.30 kcal/mol, indicating that [Cu₂(Indo)₄] is less stable than its acetate analog. Nonetheless, the data suggest that the indomethacin-based complex is thermodynamically favored in ethanolic solution, likely existing in equilibrium with monomeric Cu(Indo)₂ species.

In conclusion, this study highlights the critical role of using appropriate theoretical protocols—including DFA and basis set selection—when modeling metal–ligand interactions of biological and pharmacological relevance. Our findings demonstrate that computational thermodynamic approaches can provide meaningful insights into the coordination chemistry of transition metal ions with bioactive molecules. These results establish a robust framework for the rational design and prediction of metal-drug complexes, contributing to the advancement of bioinorganic chemistry and the growing interface between computational biology and medicinal chemistry.

## Supplementary Information

Below is the link to the electronic supplementary material.

Supplementary file1 (DOCX 5055 KB)

## Data Availability

No datasets were generated or analysed during the current study.
