# Supplementary material for: Benchmarking CuₙO (n = 1, 2) complexes via ab initio methods: structural, electronic, and thermodynamic insights with biochemical relevance
Source: J Mol Model. 2025 Dec 3;32(1):3. doi: 10.1007/s00894-025-06538-x (PMC12675678; doi:10.1007/s00894-025-06538-x)
Supplement: Supplementary file 1 — Supplementary file1 (DOCX 5055 KB) [file 894_2025_6538_MOESM1_ESM.docx]

**Supporting Information**

**Benchmarking CuₙO (n = 1, 2) Complexes via Ab Initio Methods: Structural, Electronic, and Thermodynamic Insights with Biochemical Relevance**

Raúl Flores ^1†^, Luis Soriano-Agueda ^2†^, Marco Franco-Pérez ^2^* and Rodolfo

Gómez-Balderas ^1^*

^1^ Laboratorio de Fisicoquímica Analítica, Unidad de Investigación Multidisciplinaria, Facultad de Estudios Superiores Cuautitlán, Universidad Nacional Autónoma de México, Cuautitlán Izcalli, C.P. 54700, Estado de México, México.

^2^ Departamento de Física y Química Teórica, Facultad de Química, Universidad Nacional Autónoma de México, Cd. Universitaria, 04510 Ciudad de México, México.

^†^ Equality contributions.

*E-mail: [rodolfo.gomez@unam.mx](mailto:rodolfo.gomez@unam.mx), [qimfranco@quimica.unam.mx](mailto:qimfranco@quimica.unam.mx).

Index

**Equilibrium distances 2**

Cu_2_2

Cu_2_^+^4

Cu_2_^-^6

CuO8

CuO^+^10

CuO^-^12

**Vibrational constants** **14**

Cu_2_14

Cu_2_^+^16

Cu_2_^-^18

CuO20

CuO^+^22

CuO^-^13

**Dissociation energies26**

**MADs for dimerization energies of Cu(II)/Indo29**

**References 29**

**Equilibrium distances MAEs**

a) Def2-SVP


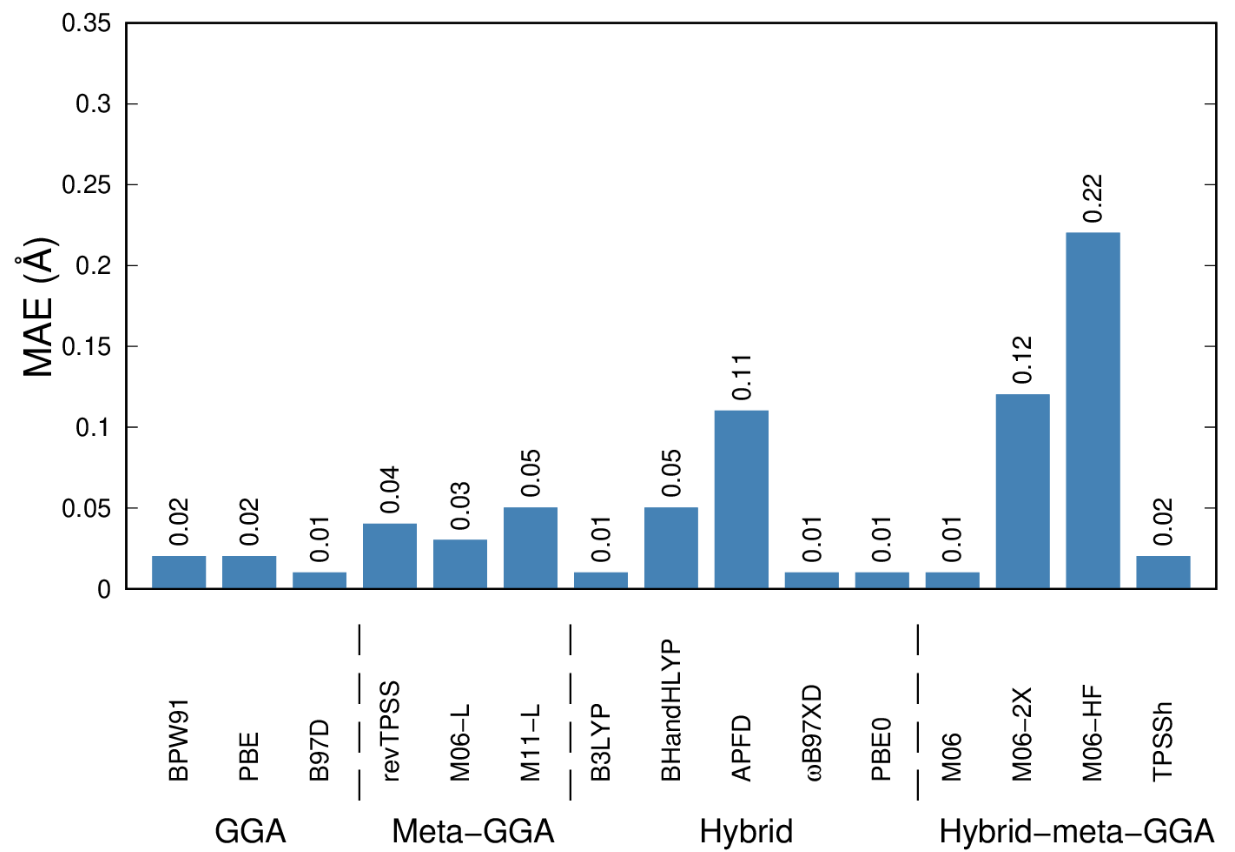


b) Def2-TZVP

**
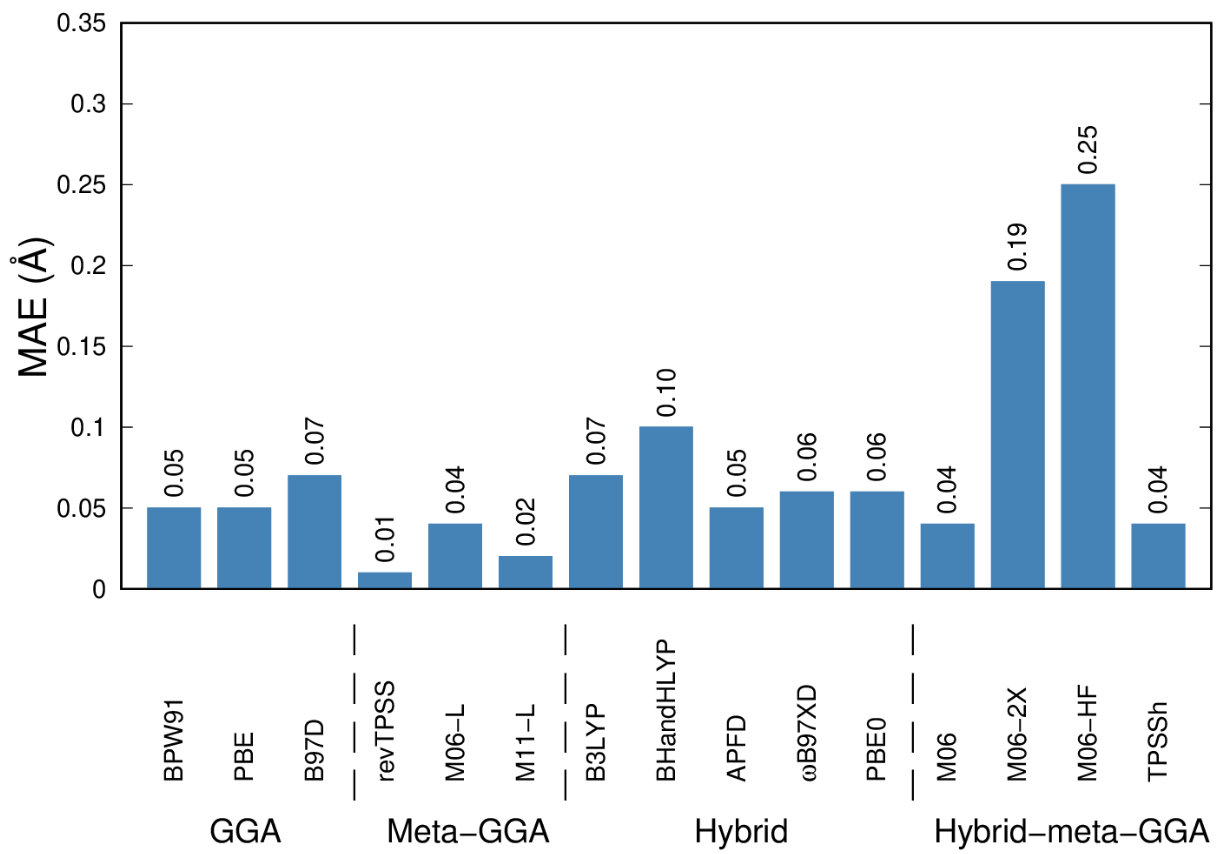
**

c) 6-31+G(d,p)


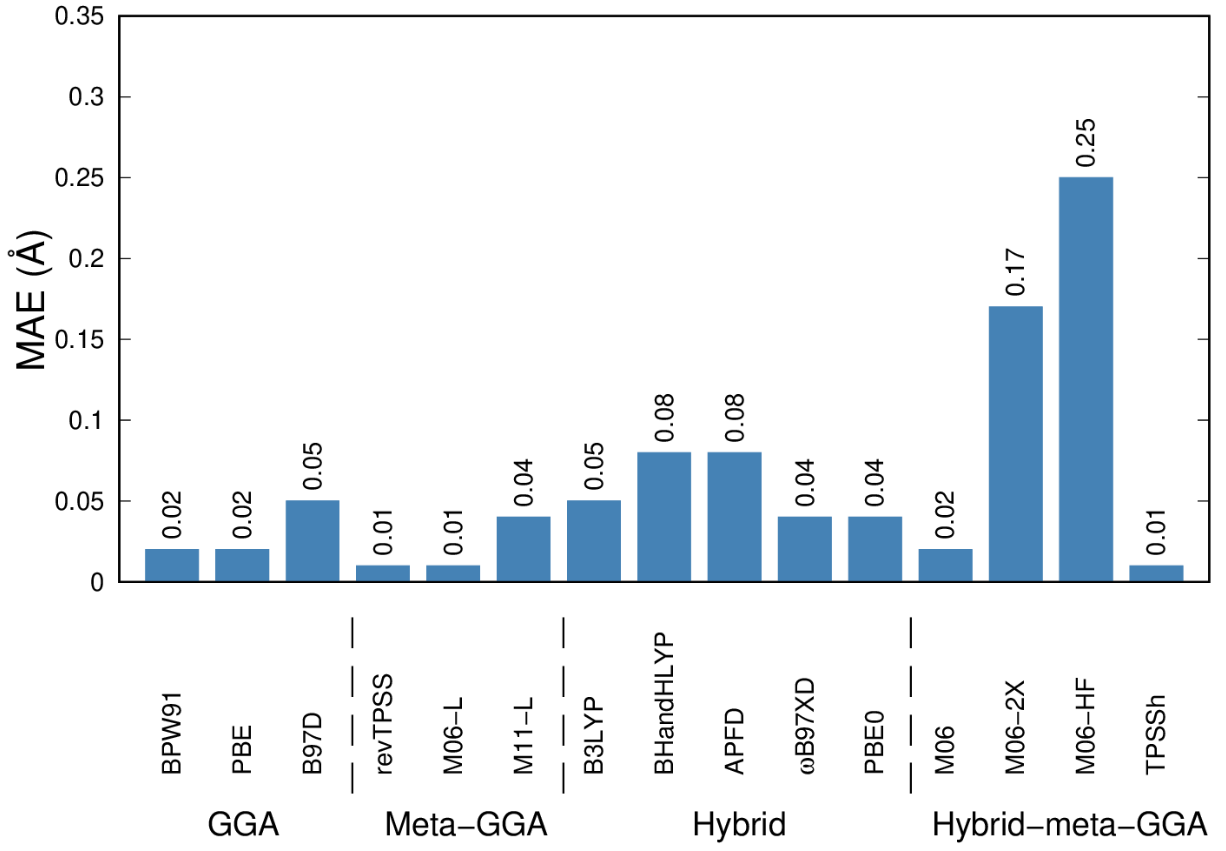


d) 6-311+G(d,p)


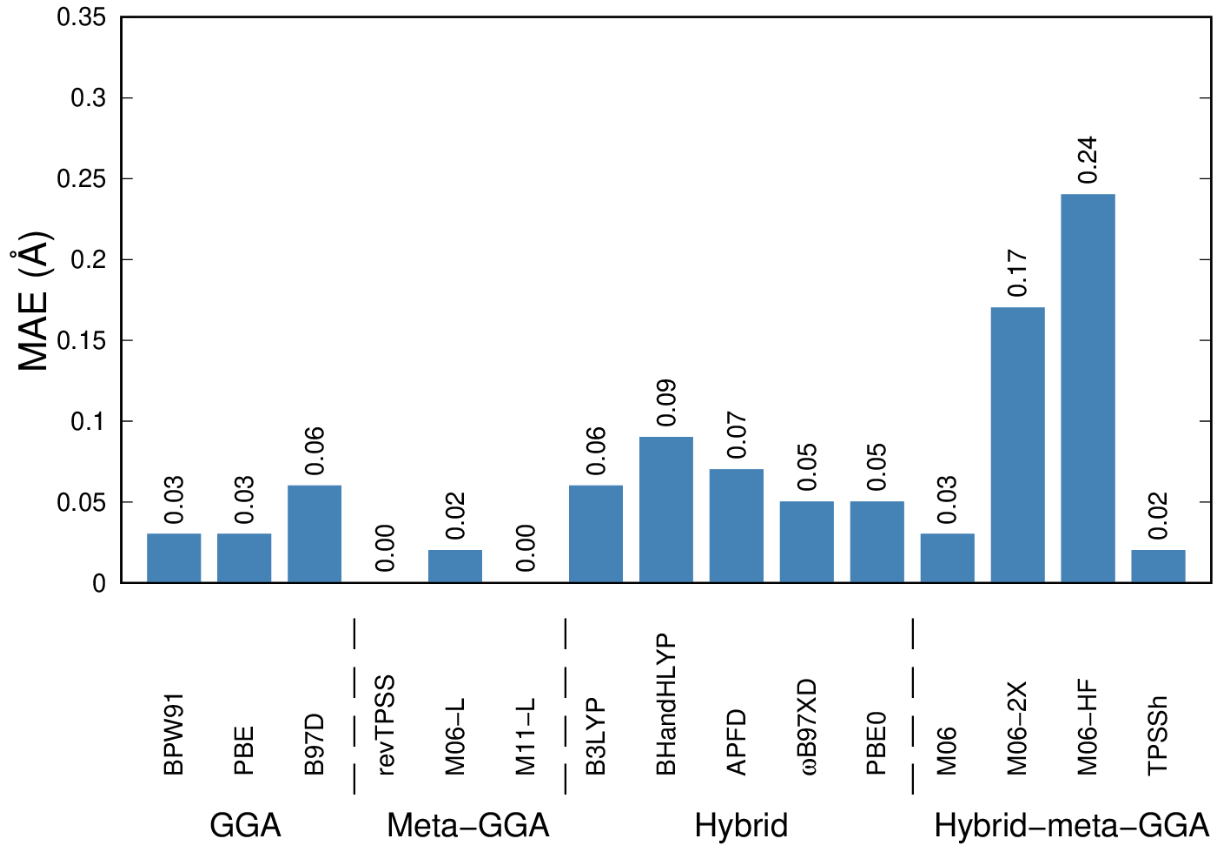


**Figure SI1**. MAEs for the equilibrium distances of ${Cu}_{2}$ calculated for each one of the DFAs. Experimental value: 2.2197 Å.[1] All values in Å.

a) Def2-SVP


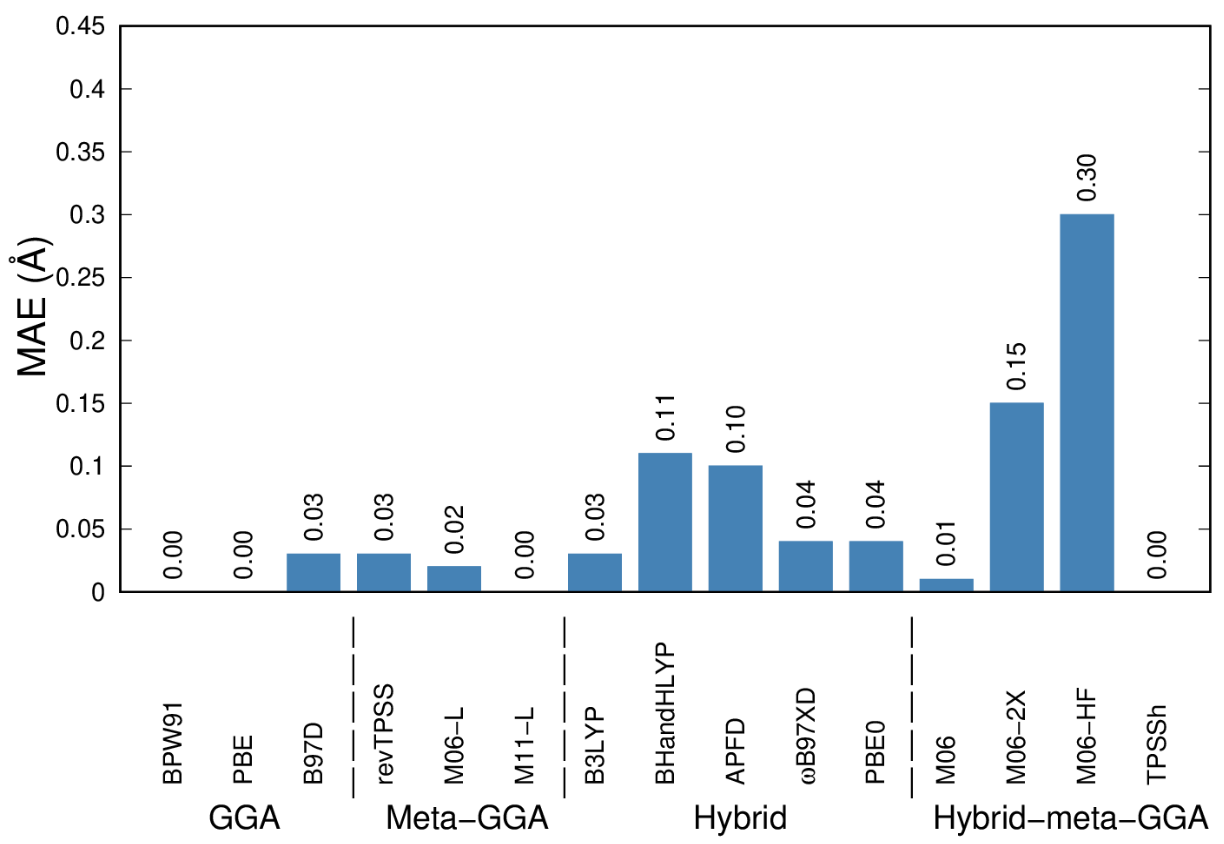


b) Def2-TZVP

**
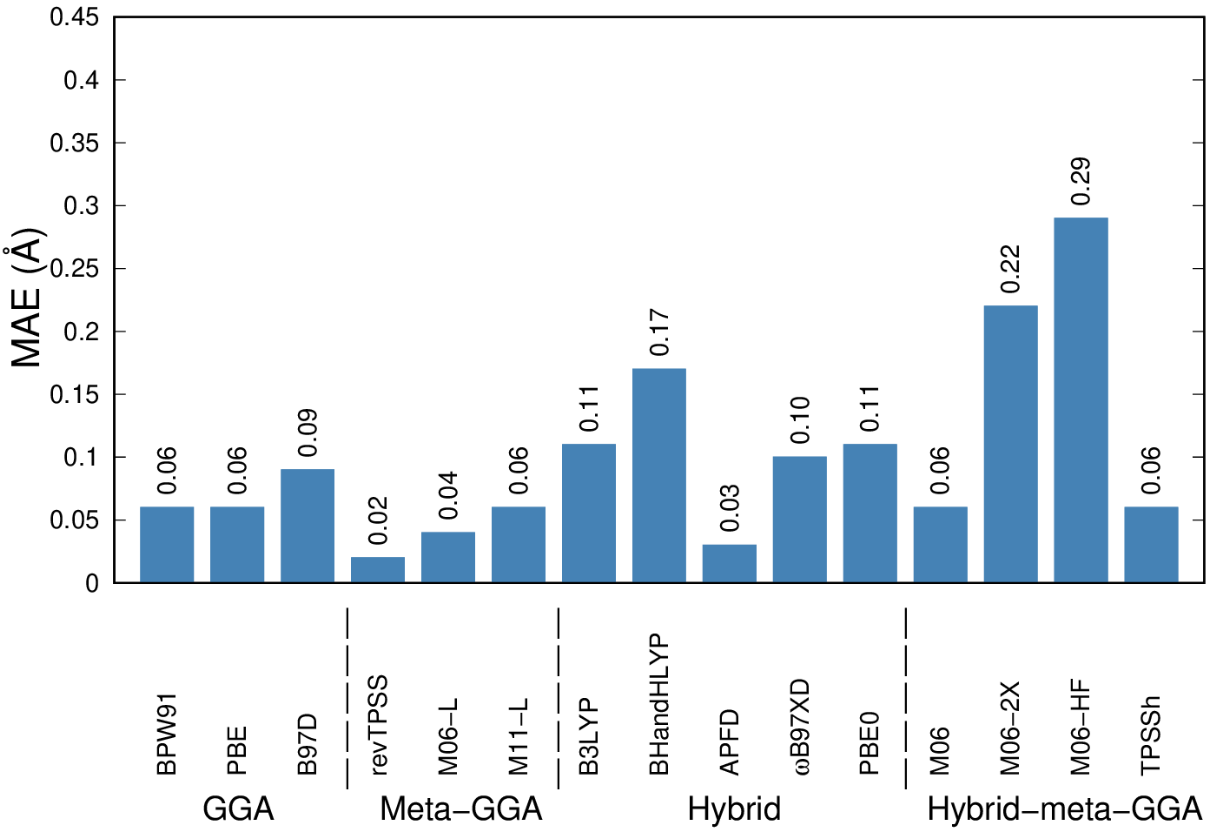
**

c) 6-31+G(d,p)


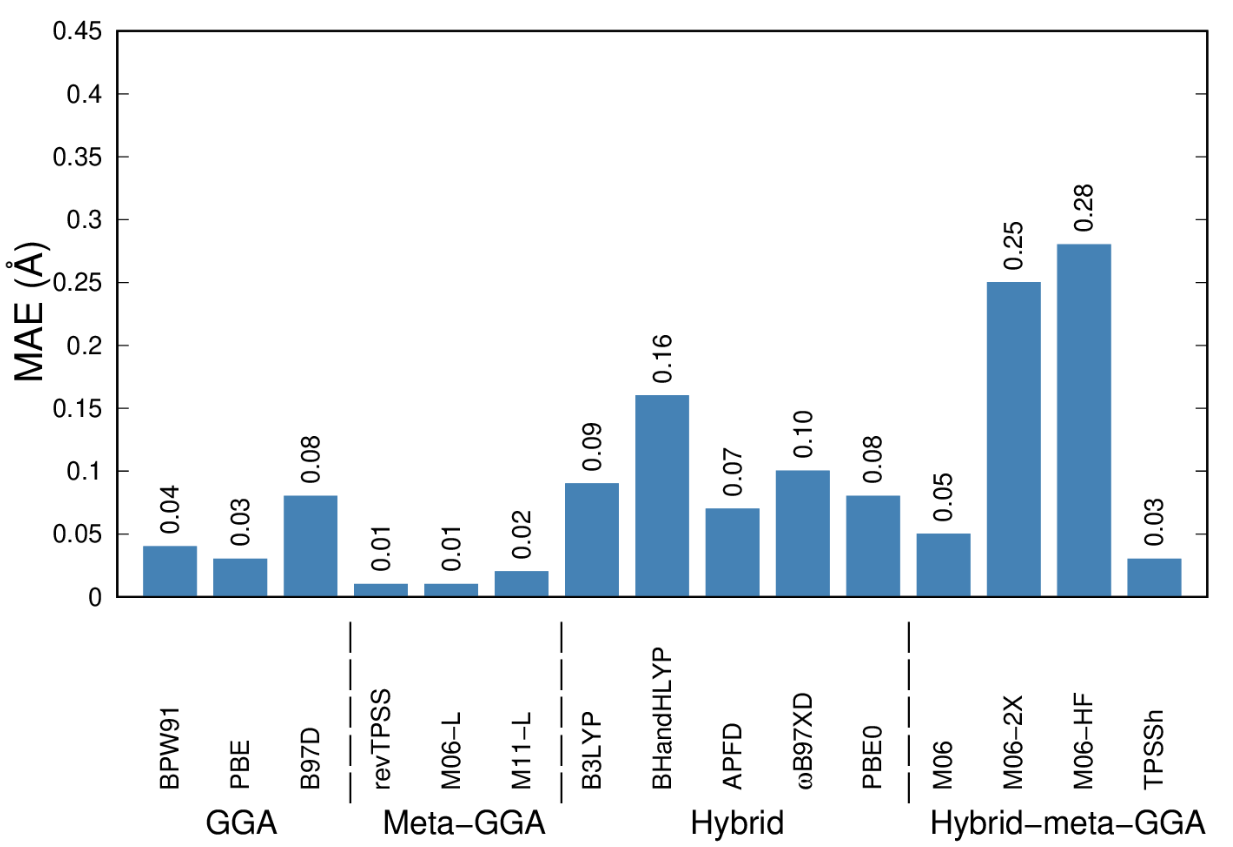


d) 6-311+G(d,p)


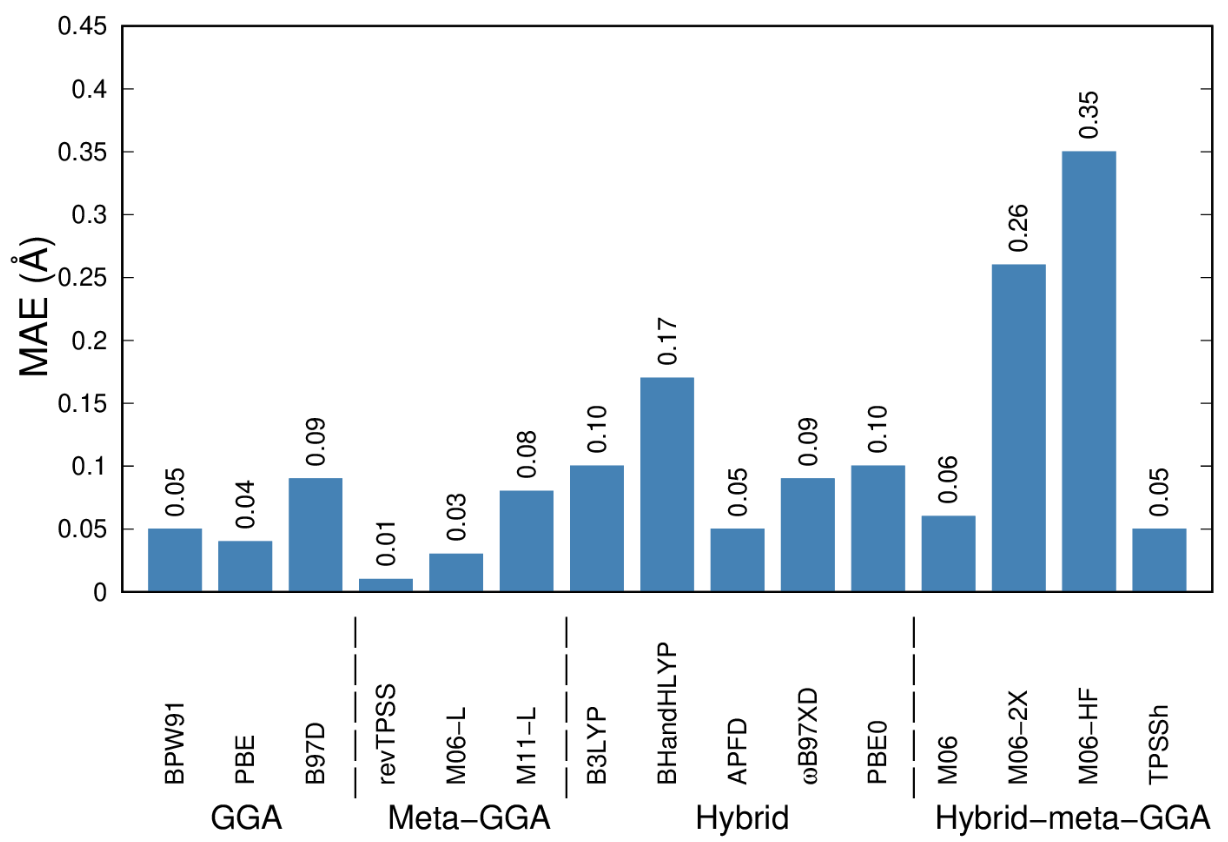


**Figure SI2**. MAEs for the equilibrium distances of ${Cu}_{2}^{+}$ calculated for each one of the DFAs. Experimental value: 2.35 Å.[2] All values in Å.

a) Def2-SVP


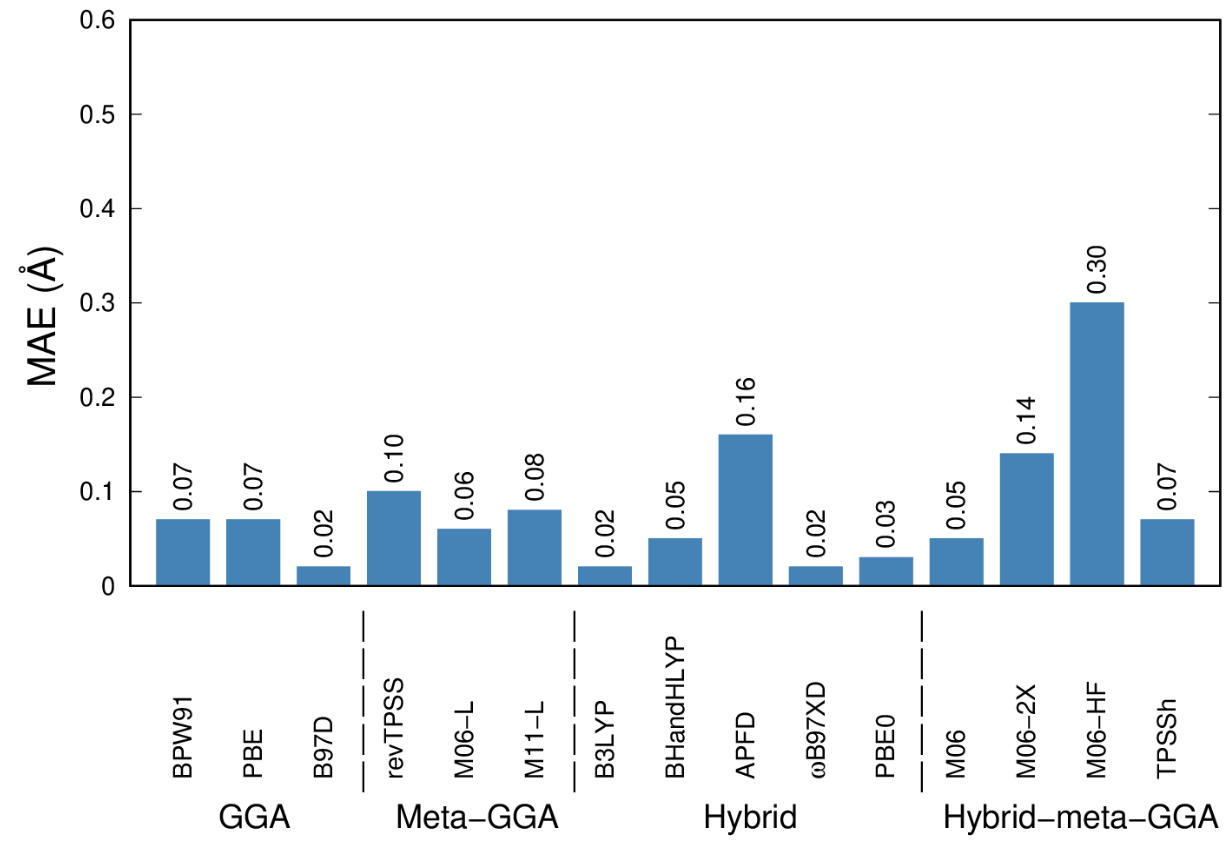


b) Def2-TZVP

**
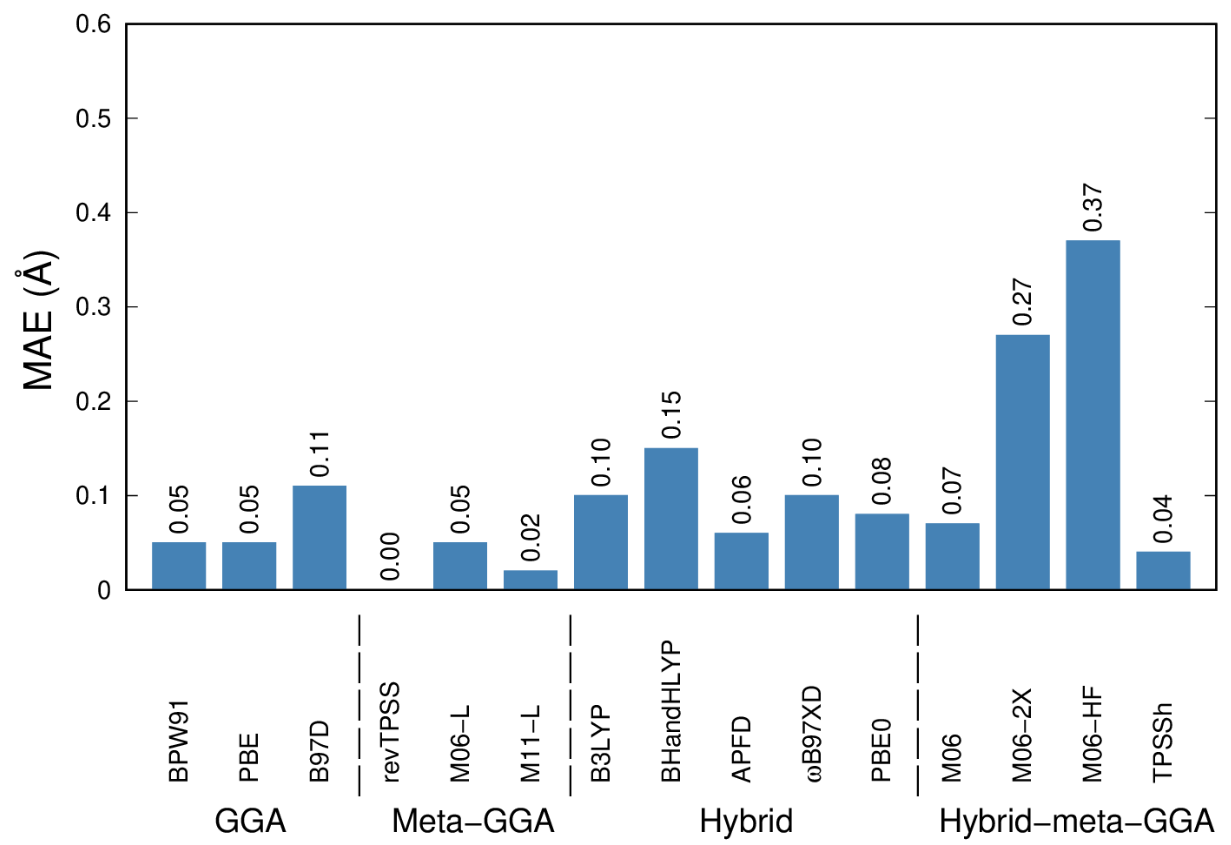
**

c) 6-31+G(d,p)


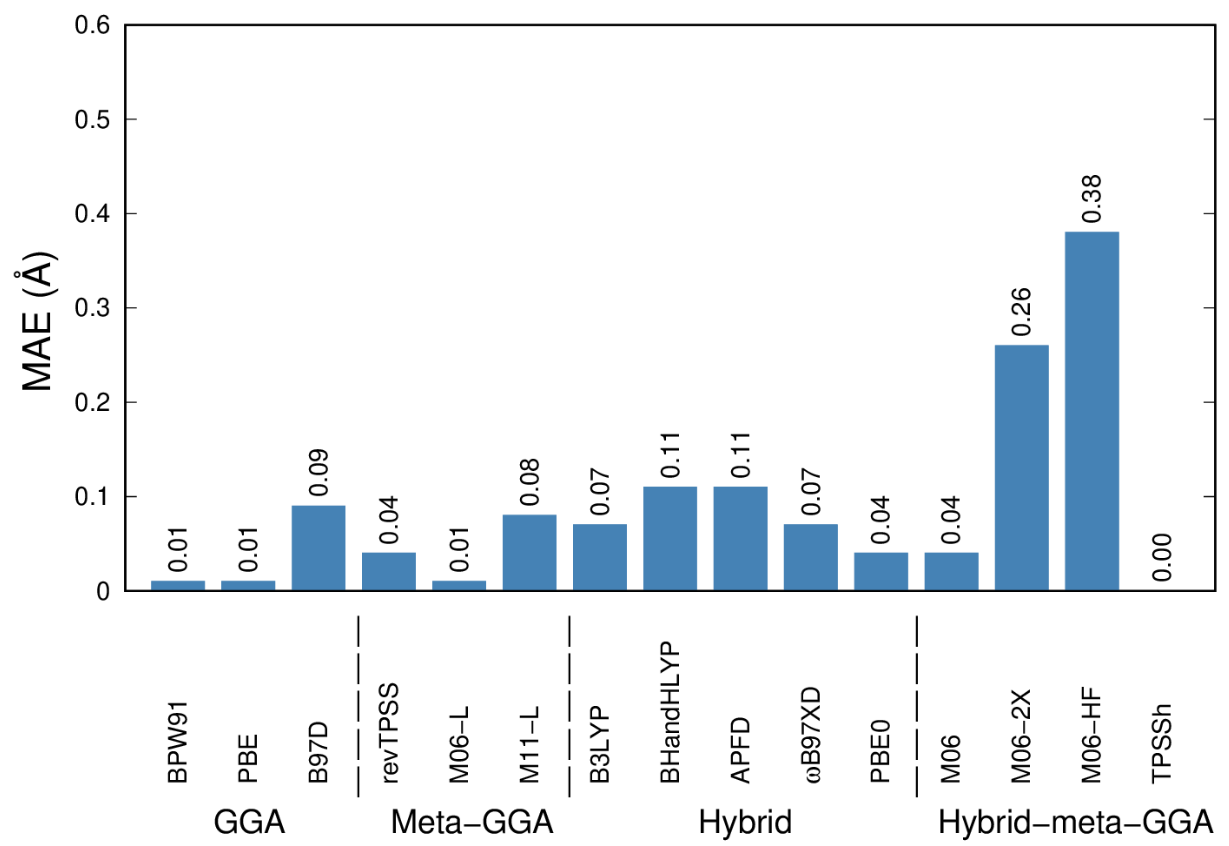


d) 6-311+G(d,p)


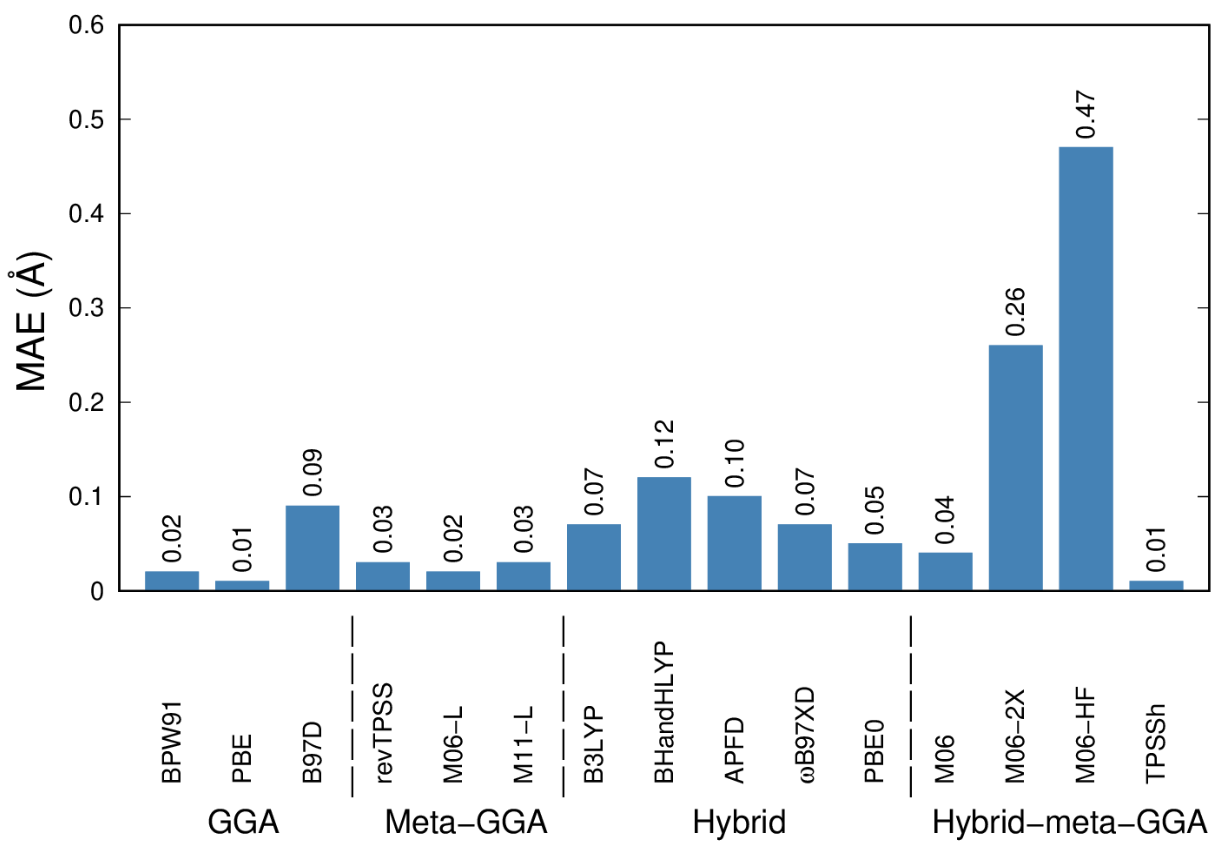


**Figure SI3**. MAEs for the equilibrium distances of ${Cu}_{2}^{-}$ calculated for each one of the DFAs. Experimental value: 2.343 Å.[1] All values in Å.

a) Def2-SVP


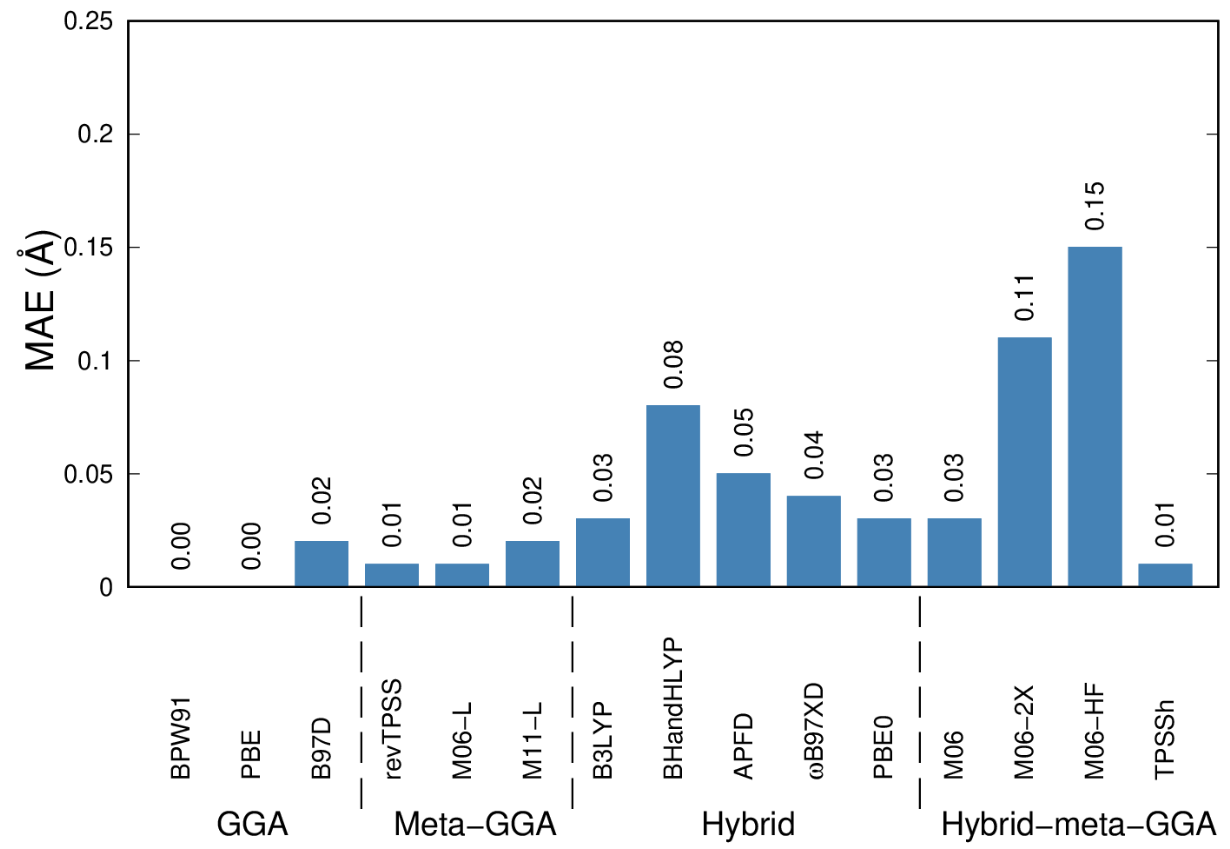


b) Def2-TZVP

**
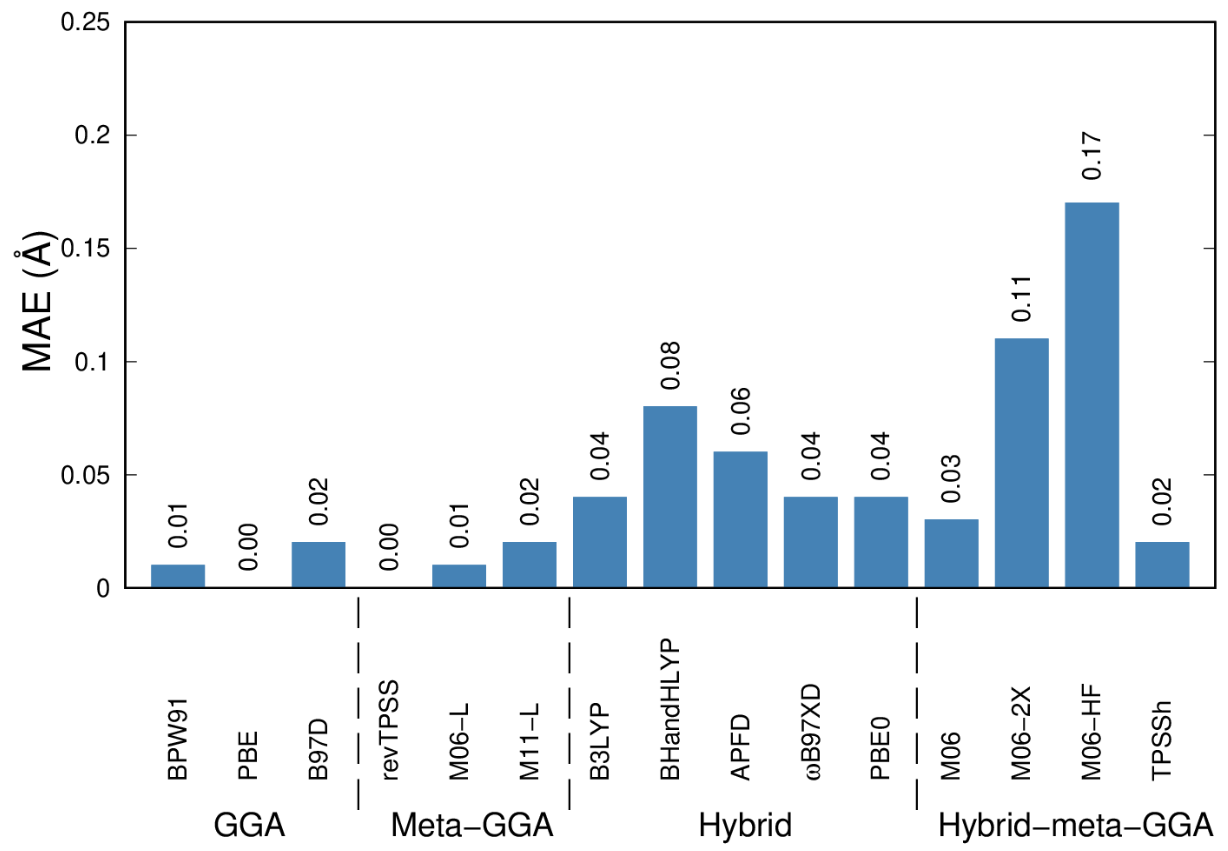
**

c) 6-31+G(d,p)


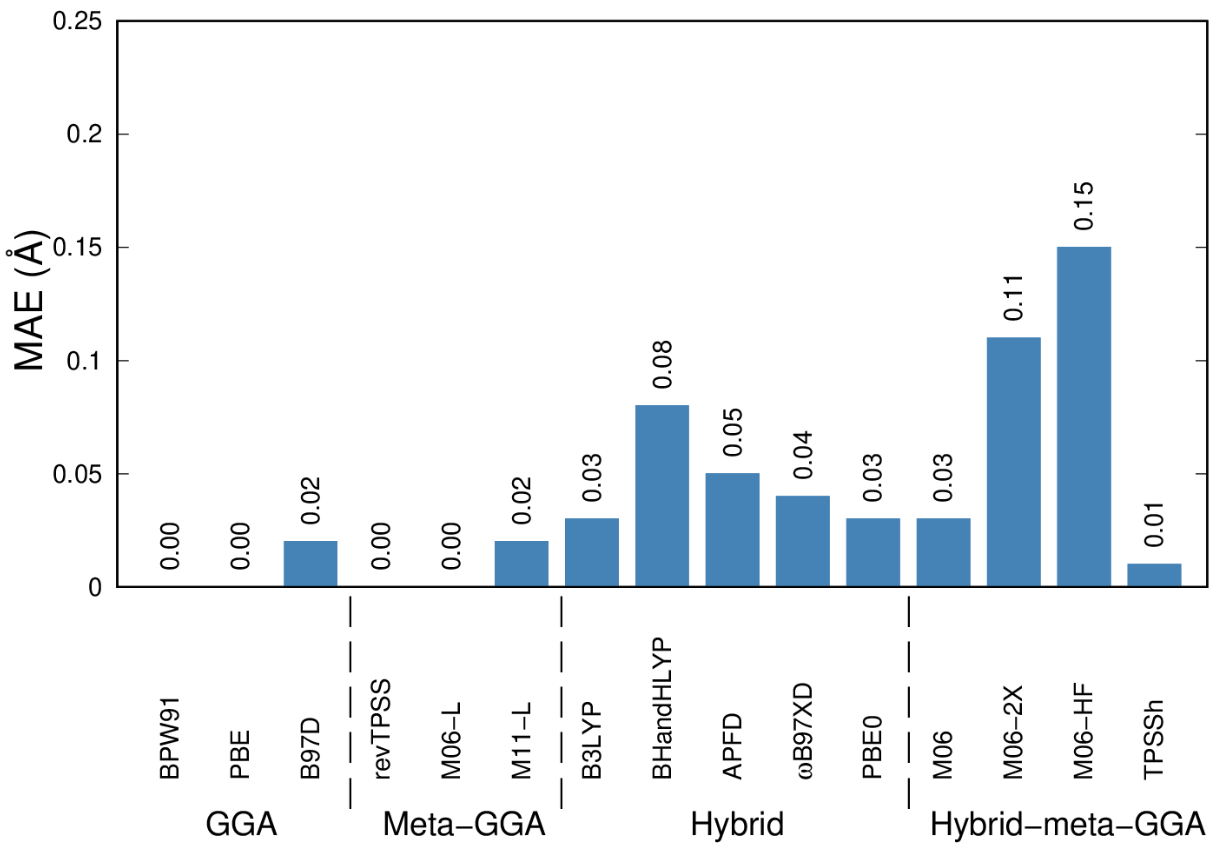


d) 6-311+G(d,p)


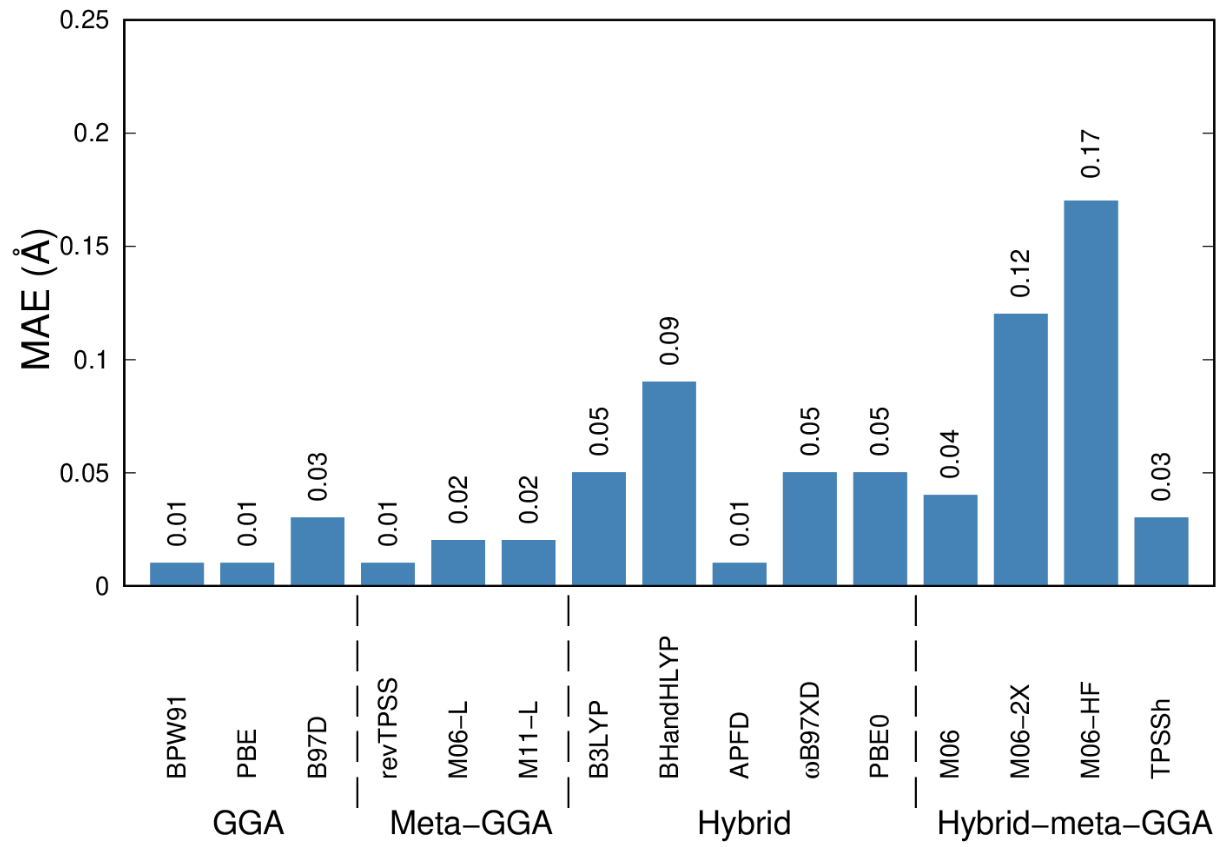


**Figure SI4**. MAEs for the equilibrium distances of $CuO$ calculated for each one of the DFAs. Experimental value: 1.724 Å.[3] All values in Å.

a) Def2-SVP


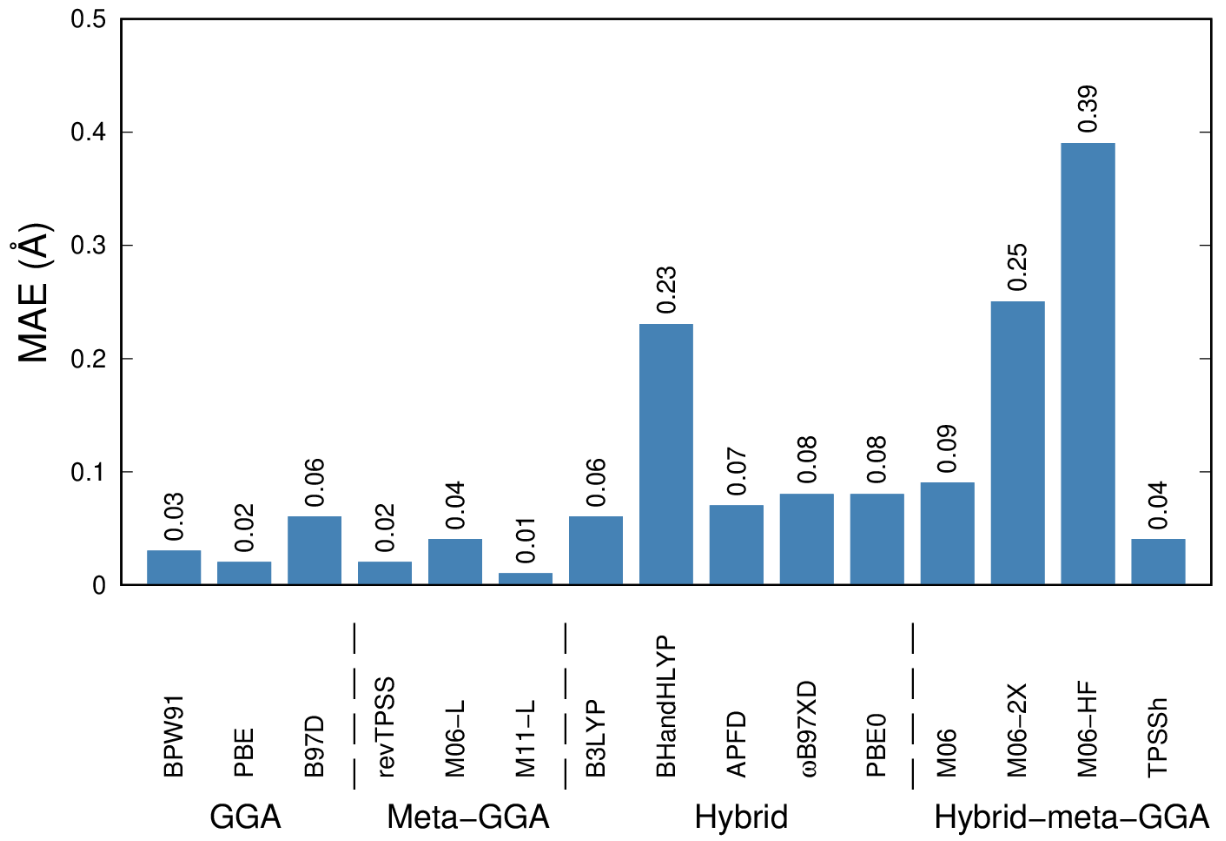


b) Def2-TZVP


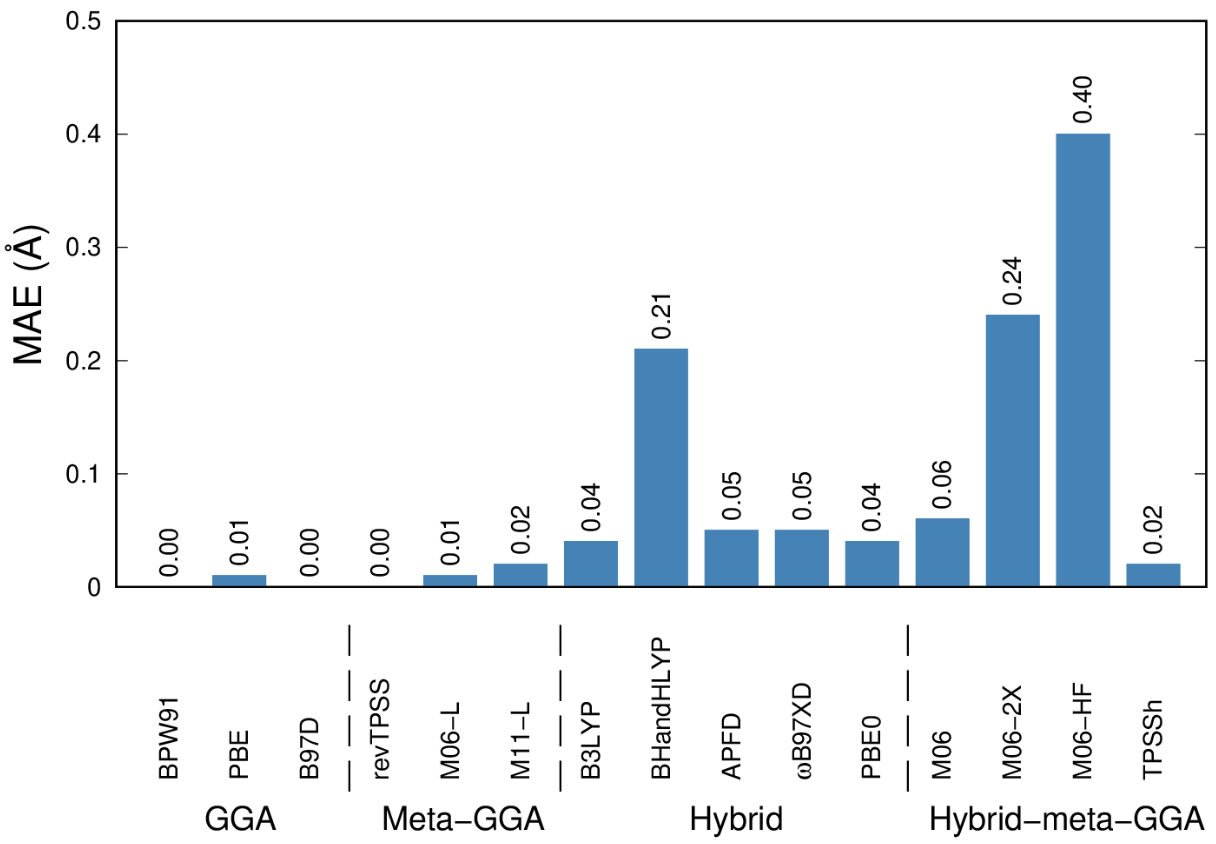


c) 6-31+G(d,p)


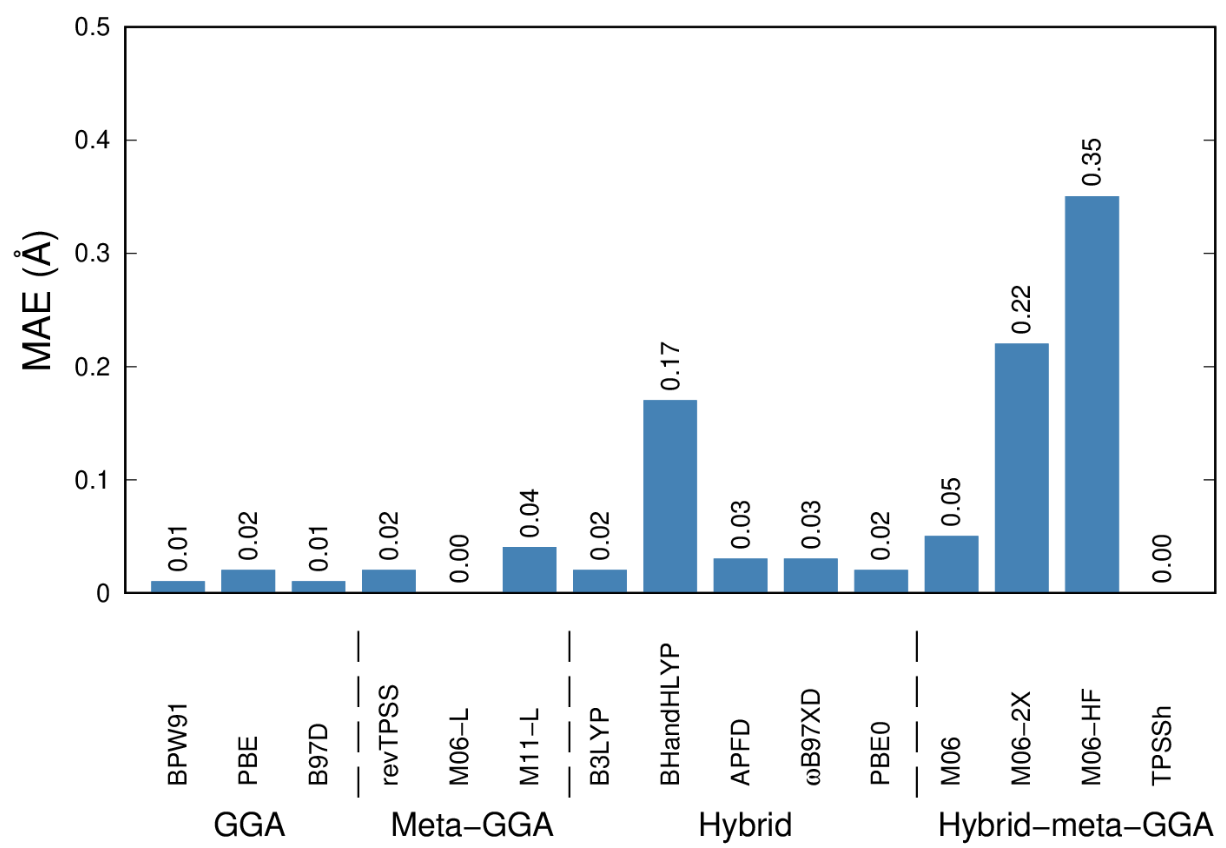


d) 6-311+G(d,p)


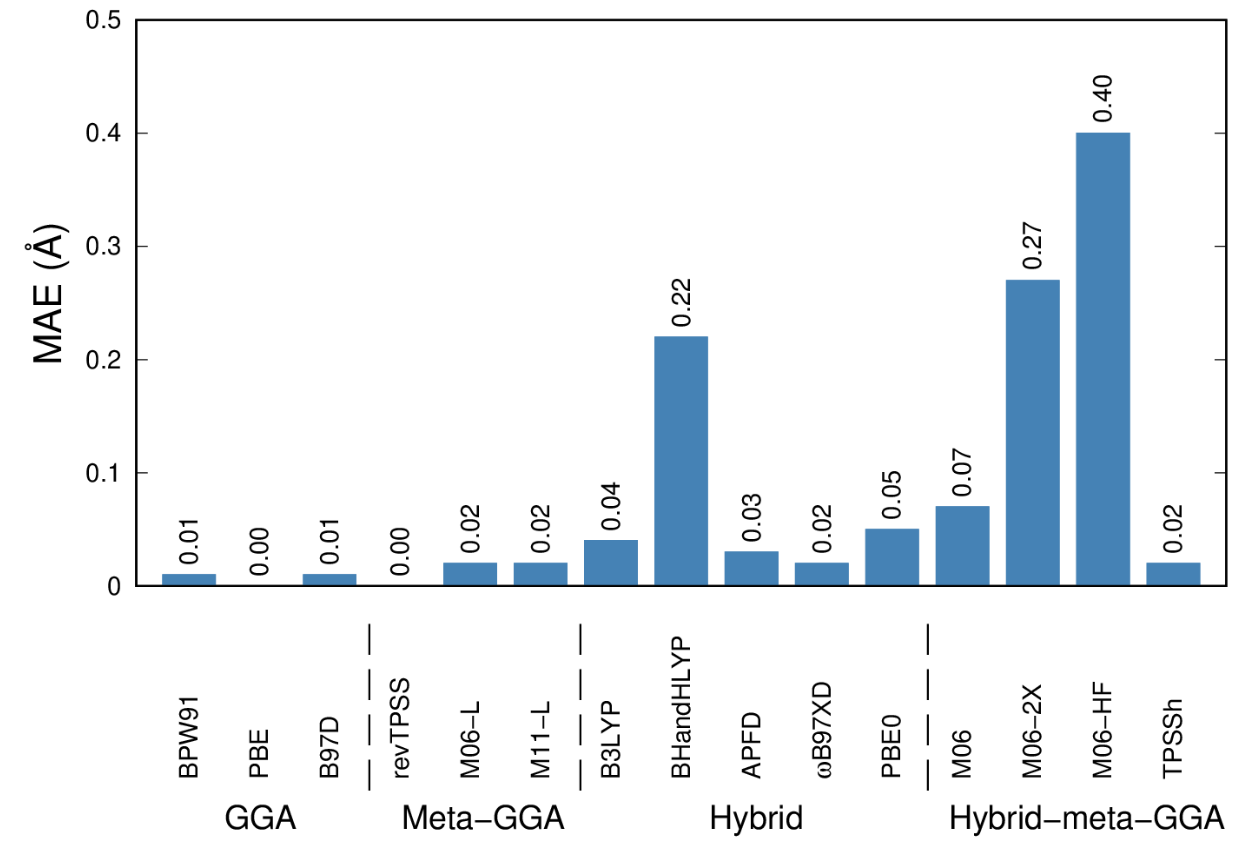


**Figure SI5**. MAEs for the equilibrium distances of ${CuO}^{+}$ calculated for each one of the DFAs. Experimental value: 1.766 Å.[4] All values in Å.

a) Def2-SVP


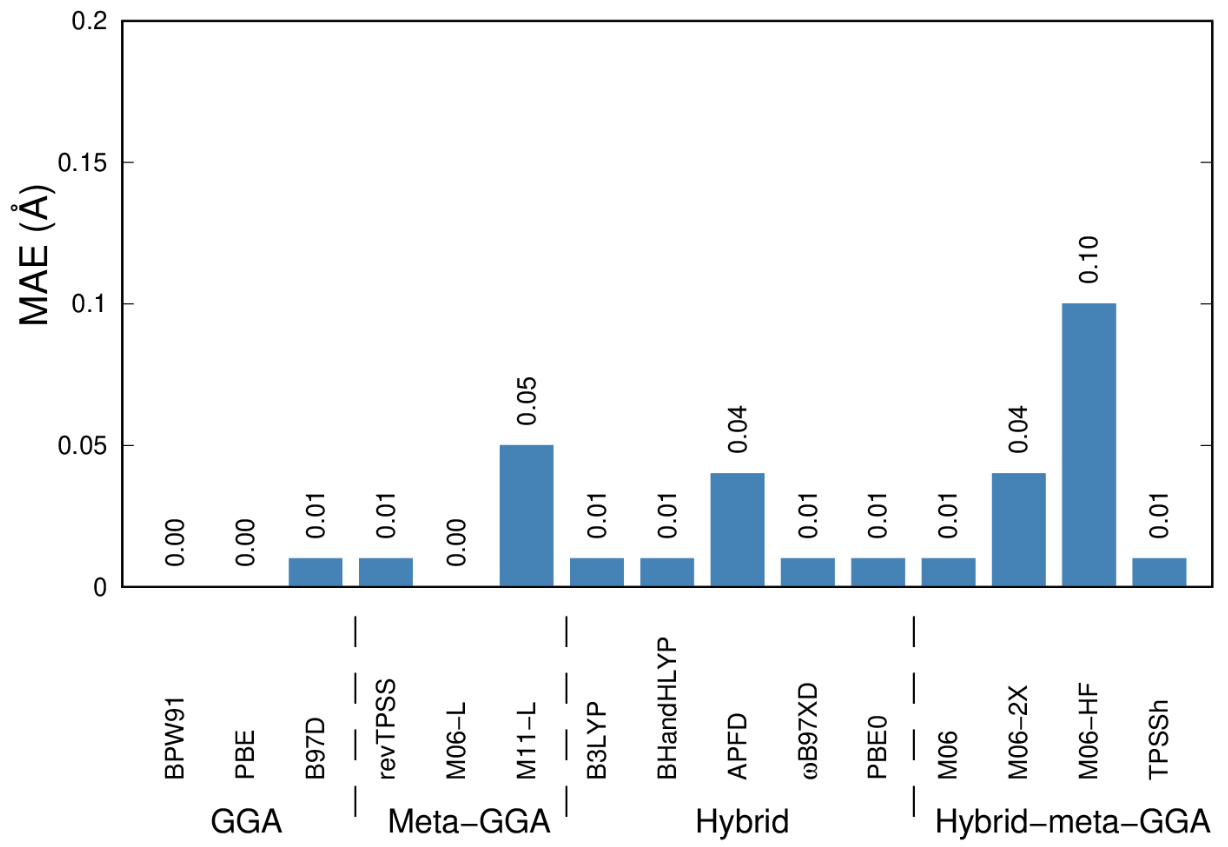


b) Def2-TZVP


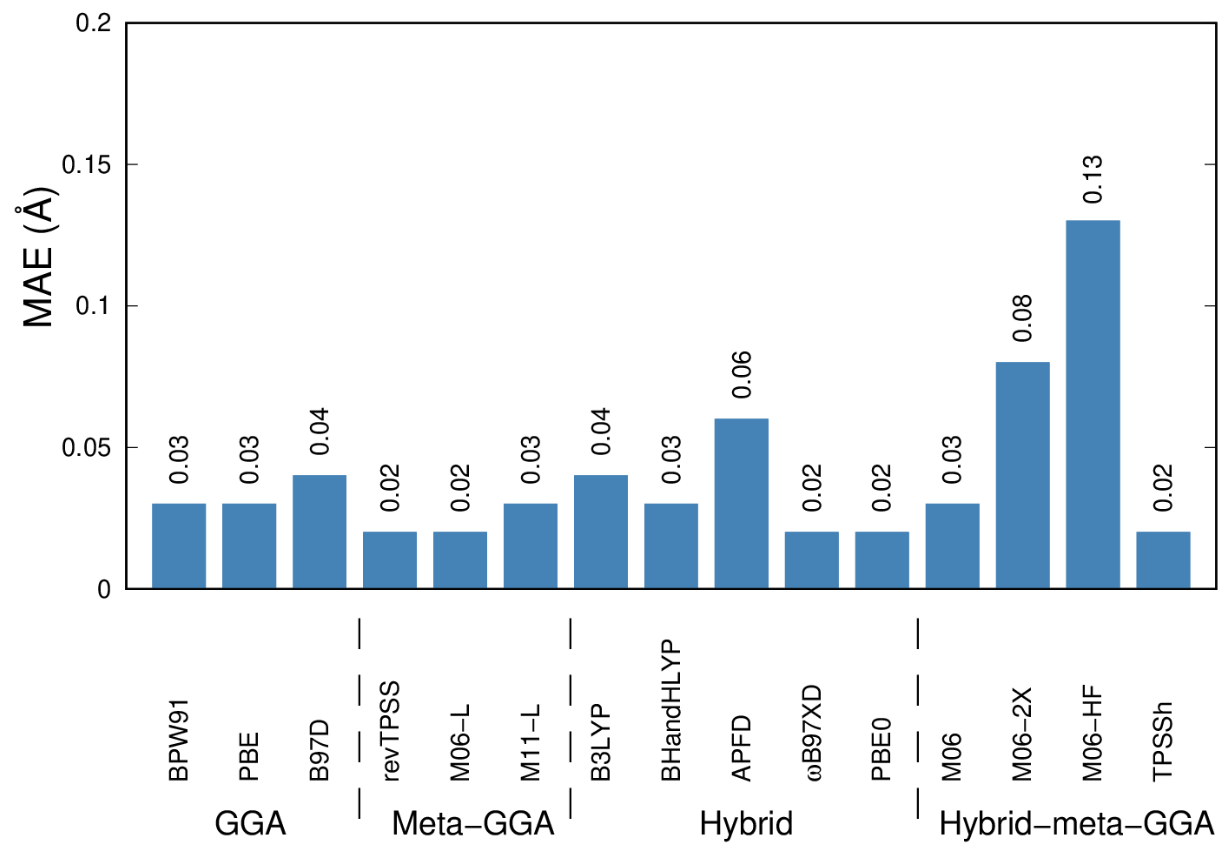


c) 6-31+G(d,p)


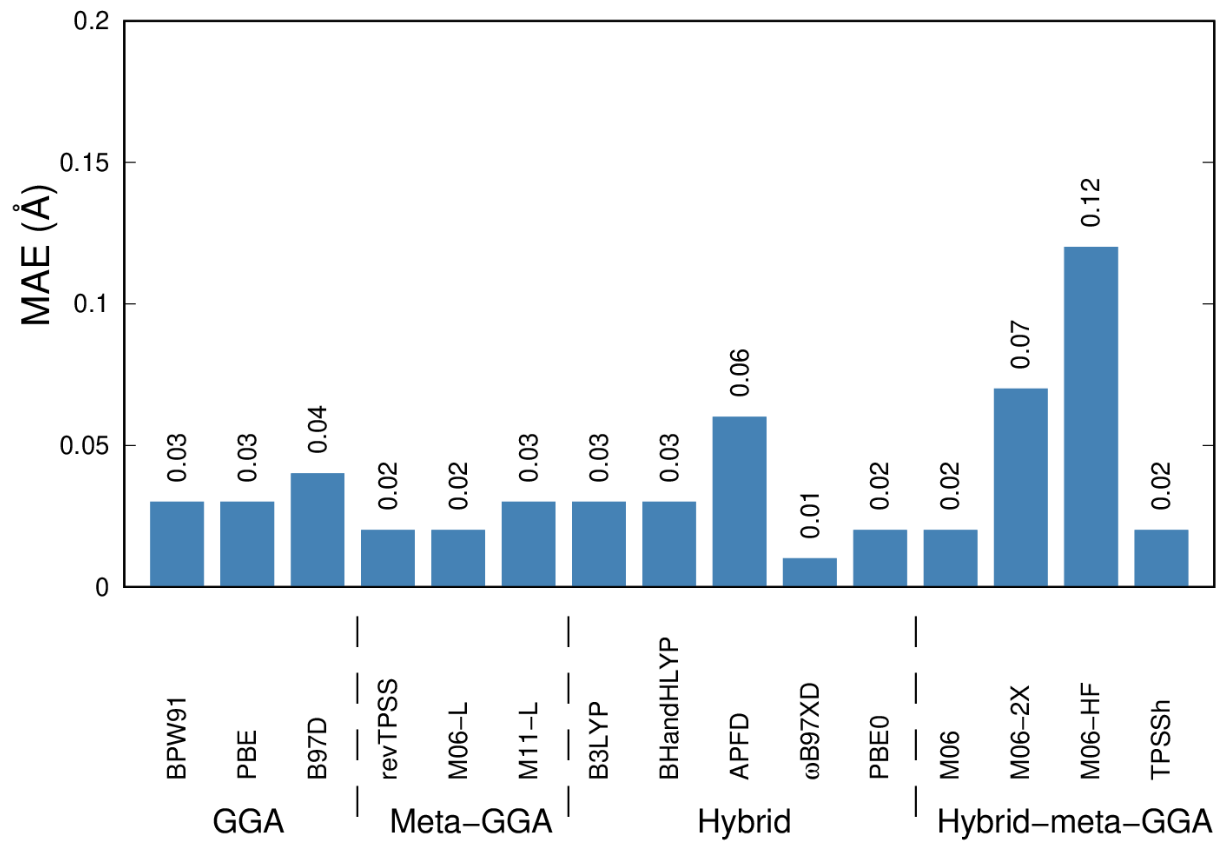


d) 6-311+G(d,p)


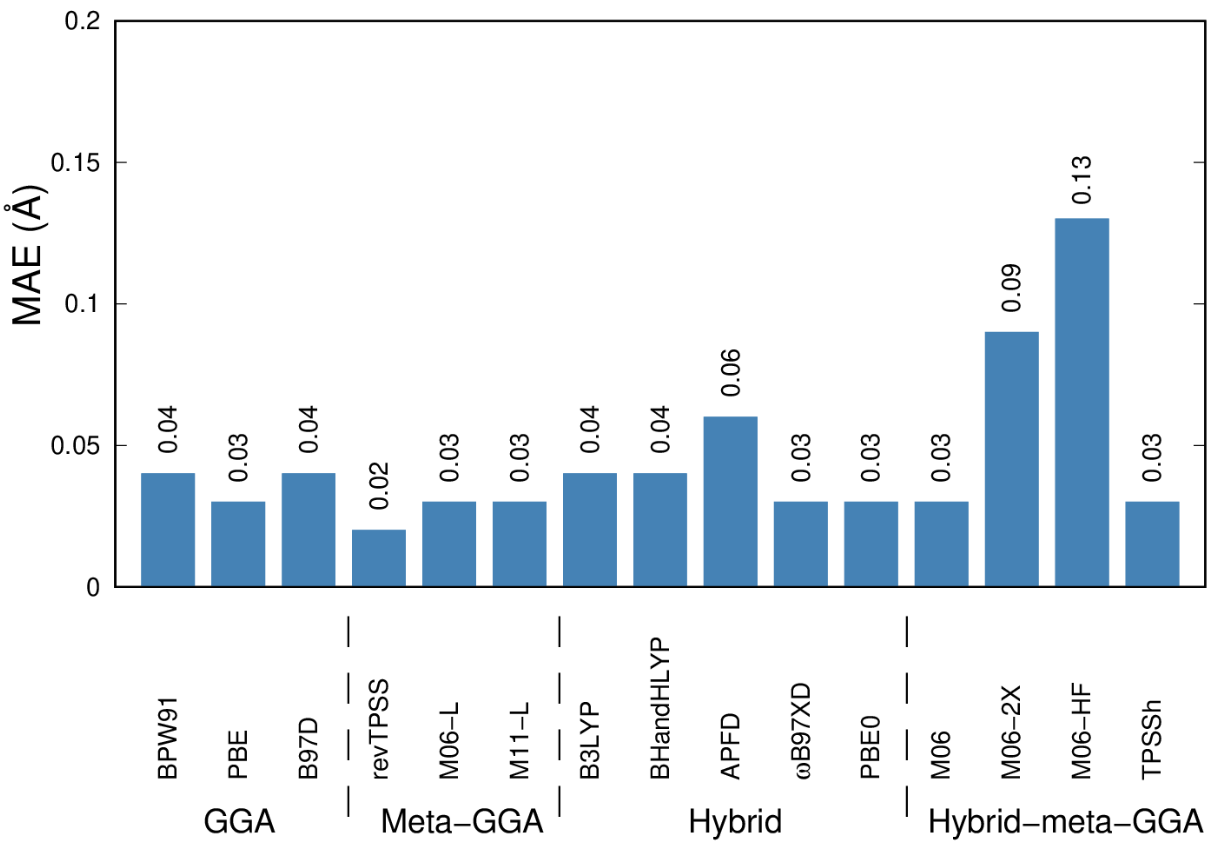


**Figure SI6**. MAEs for the equilibrium distances of ${CuO}^{-}$ calculated for each one of the DFAs. Experimental value: 1.67 Å.[3] All values in Å.

**Vibrational constants MAEs**

a) Def2-SVP

**
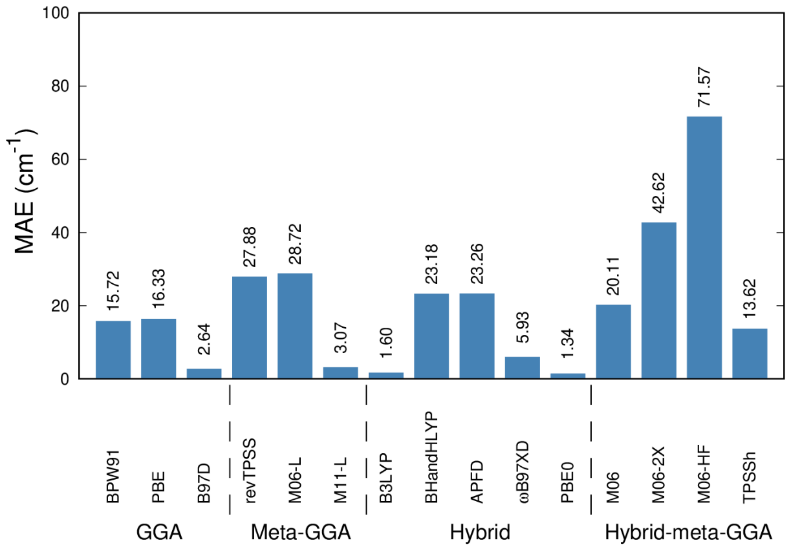
**

b) Def2-TZVP

**
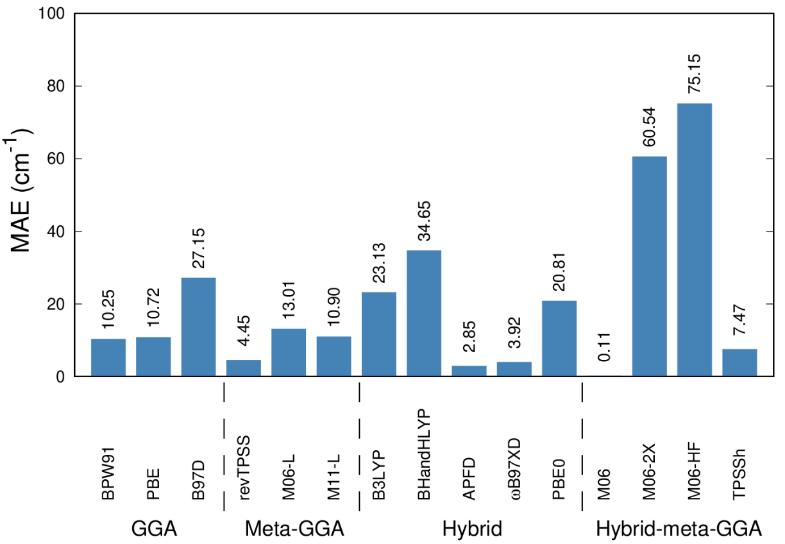
**

c) 6-31+G(d,p)

**
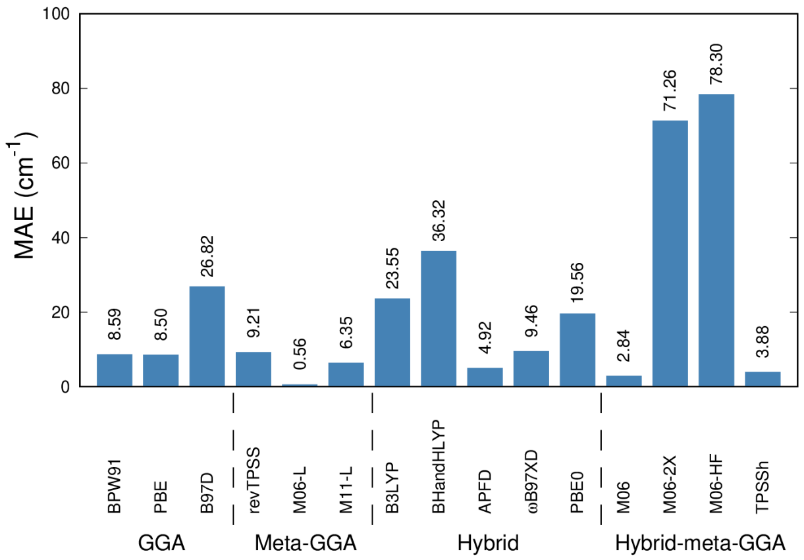
**

d) 6-311+G(d,p)

**
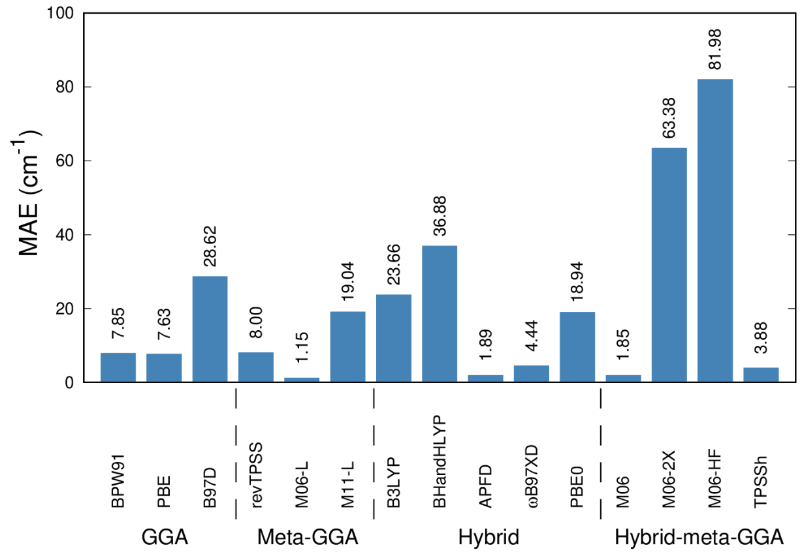
**

**Figure SI7**. MAEs for the vibrational constants of ${Cu}_{2}$ calculated for each one of the DFAs. Experimental value: 266.43 cm^-1^.[1] All values in cm^-1^.

a) Def2-SVP


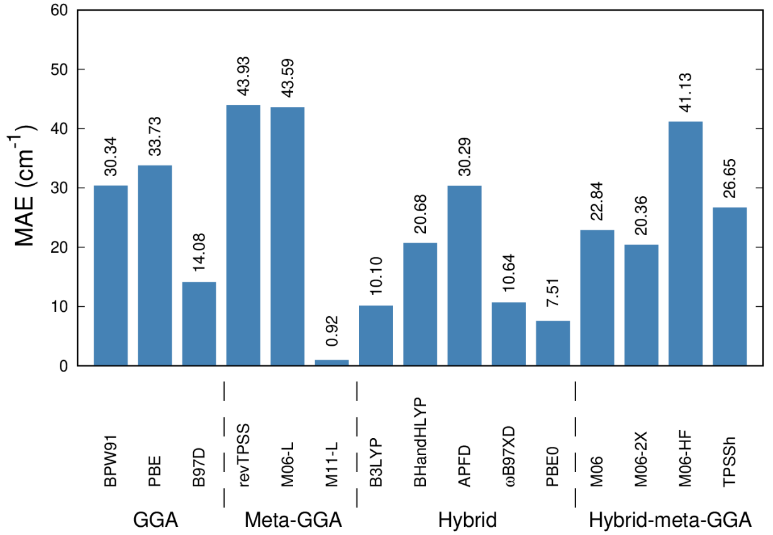


b) Def2-TZVP


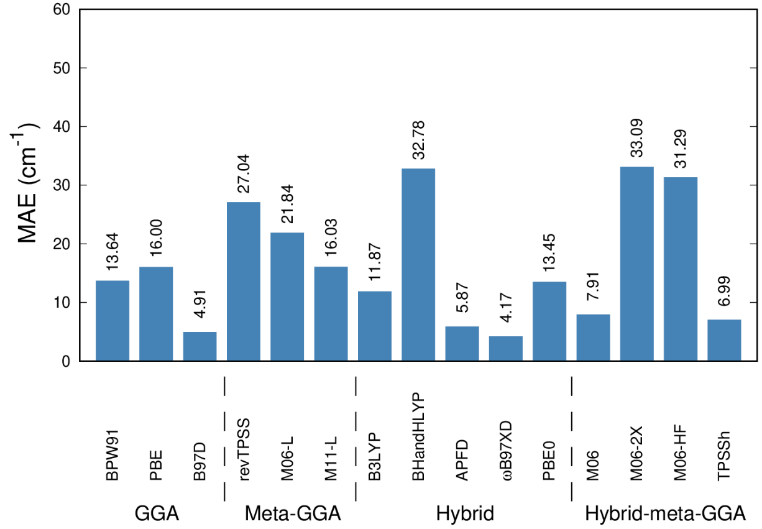


c) 6-31+G(d,p)

**
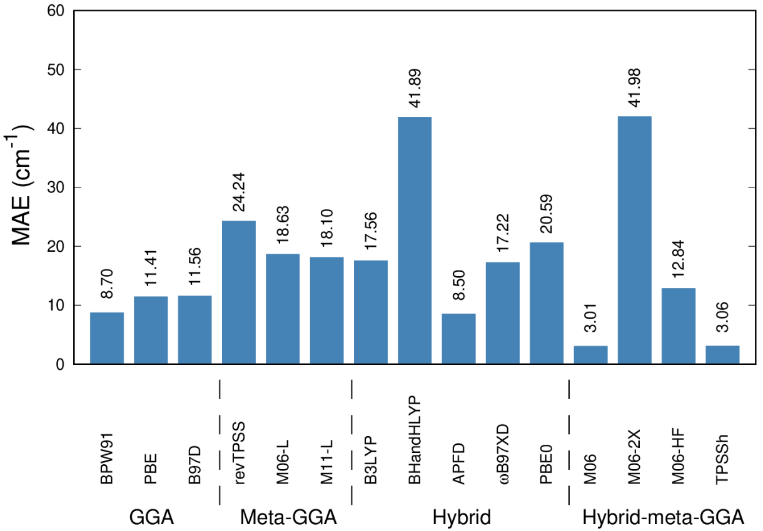
**

d) 6-311+G(d,p)

**
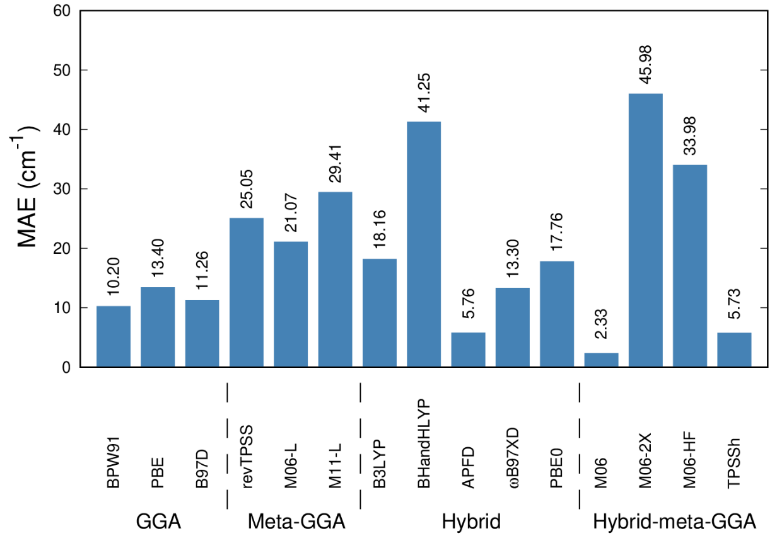
**

**Figure SI8**. MAEs for the vibrational constants of ${Cu}_{2}^{+}$ calculated for each one of the DFAs. Experimental value: 188 cm^-1^.[2] All values in cm^-1^.

a) Def2-SVP

**
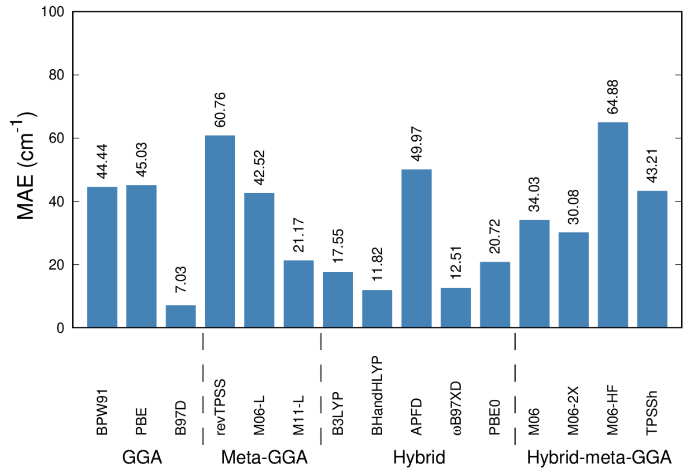
**

b) Def2-TZVP

**
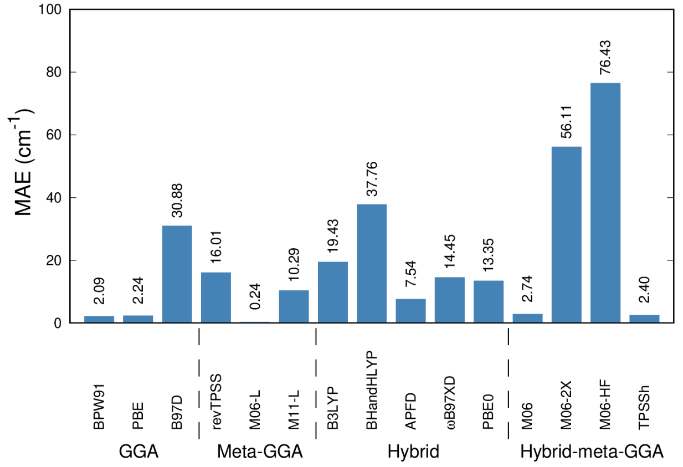
**

c) 6-31+G(d,p)

**
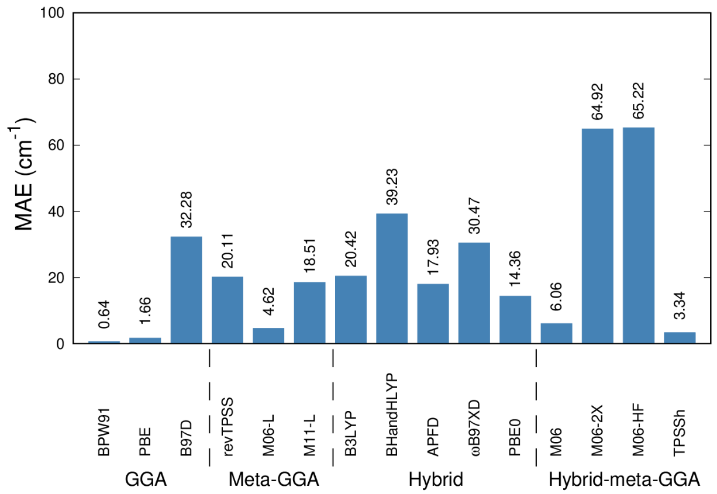
**

d) 6-311+G(d,p)

**
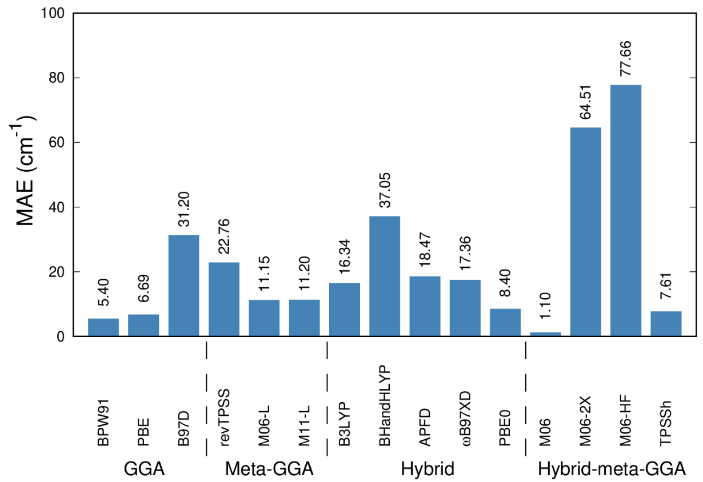
**

**Figure SI9.** MAEs for the vibrational constants of ${Cu}_{2}^{-}$ calculated for each one of the DFAs. Experimental value: 196 cm^-1^.[1] All values in cm^-1^.

a) Def2-SVP

**
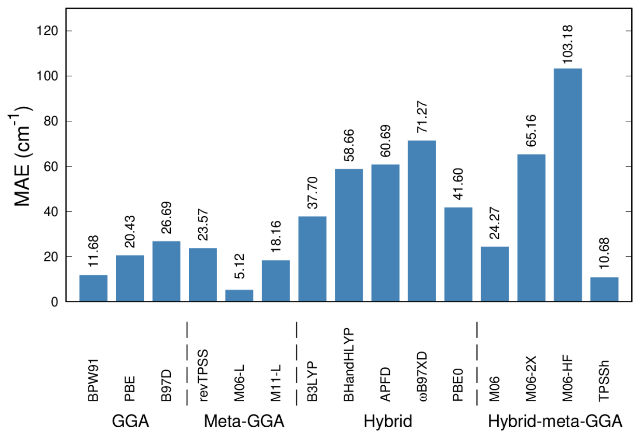
**

b) Def2-TZVP

**
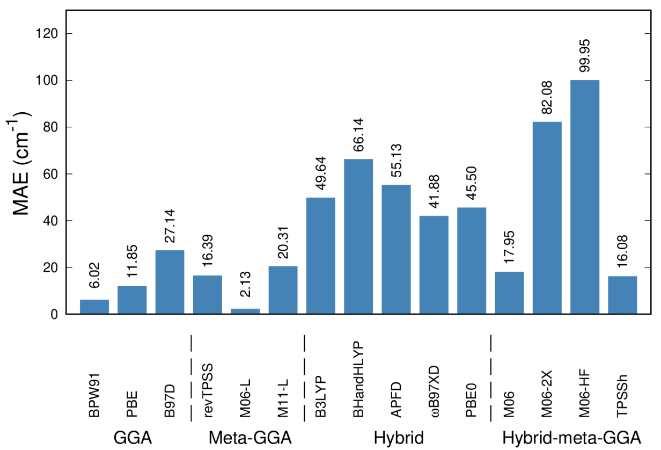
**

c) 6-31+G(d,p)

**
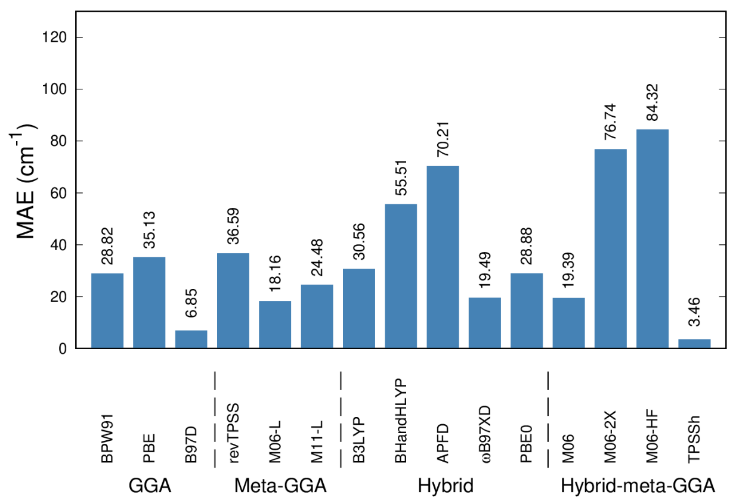
**

d) 6-311+G(d,p)

**
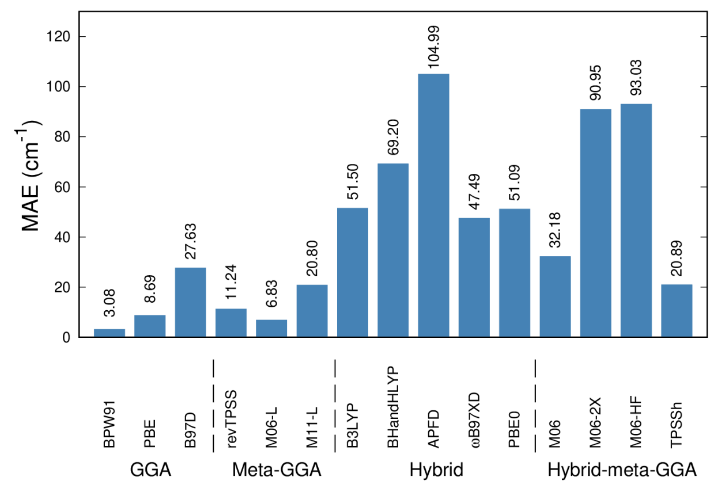
**

**Figure SI10.** MAEs for the vibrational constants of $CuO$ calculated for each one of the DFAs. Experimental value: 640.2 cm^-1^.[3] All values in cm^-1^.

a) Def2-SVP

**
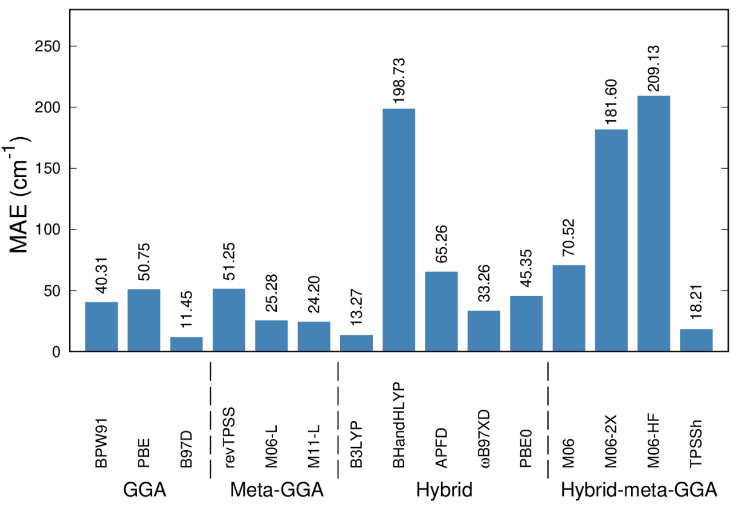
**

b) Def2-TZVP

**
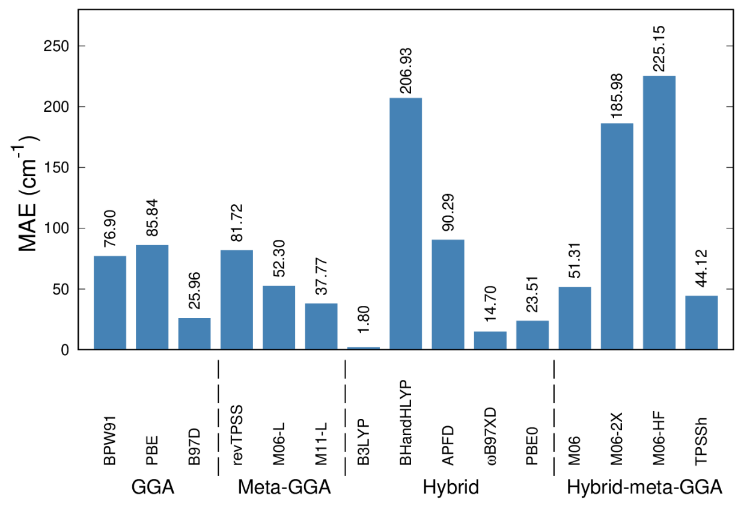
**

c) 6-31+G(d,p)

**
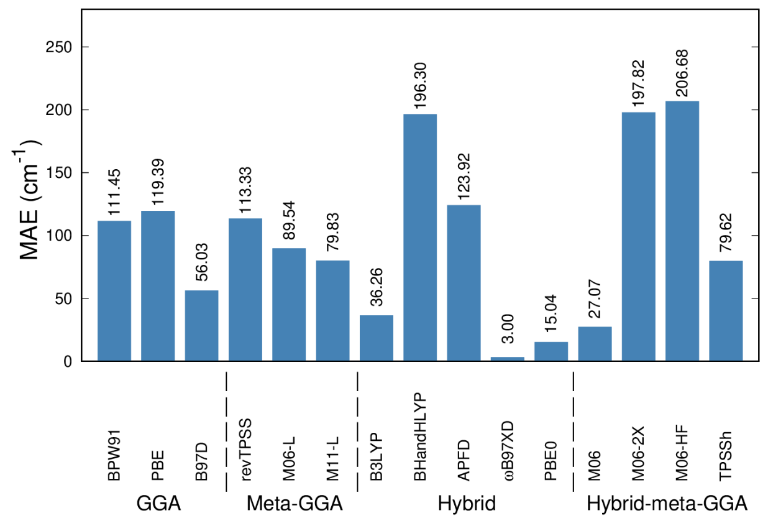
**

d) 6-311+G(d,p)

**
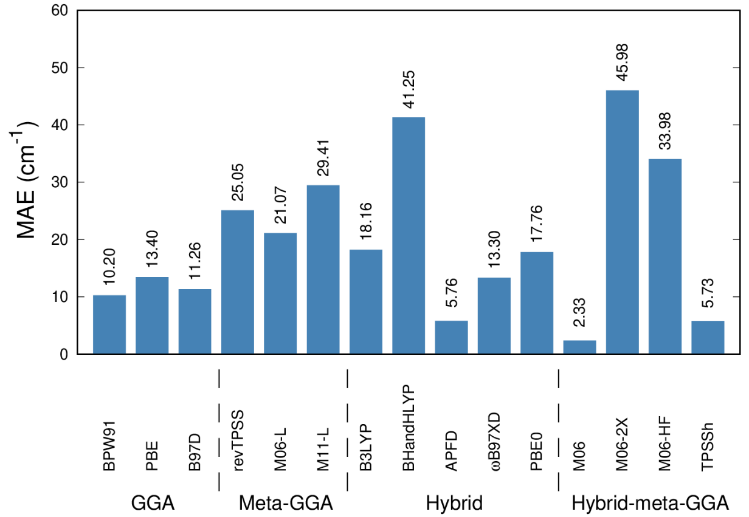
**

**Figure SI11.** MAEs for the vibrational constants of ${CuO}^{+}$ calculated for each one of the DFAs. Theoretical value (494 cm^-1^) obtained with CCSD(T)/CBS.[4] All values in cm^-1^.

a) Def2-SVP

**
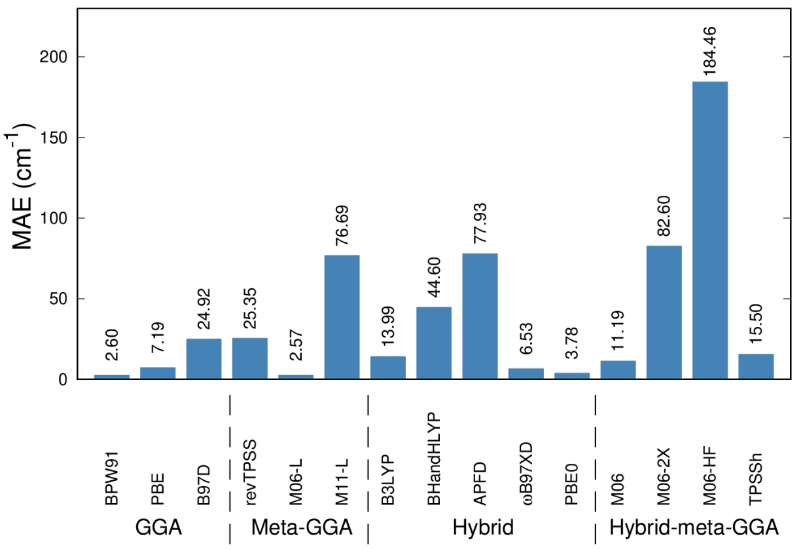
**

b) Def2-TZVP

**
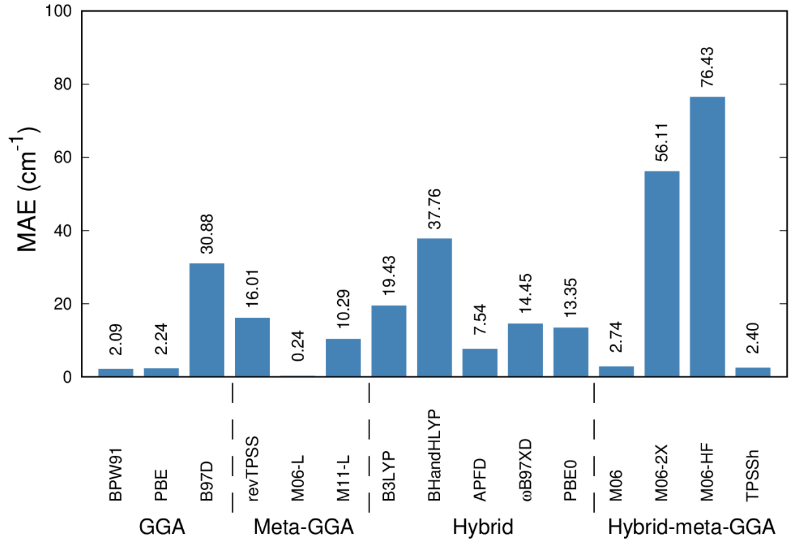
**

c) 6-31+G(d,p)

**
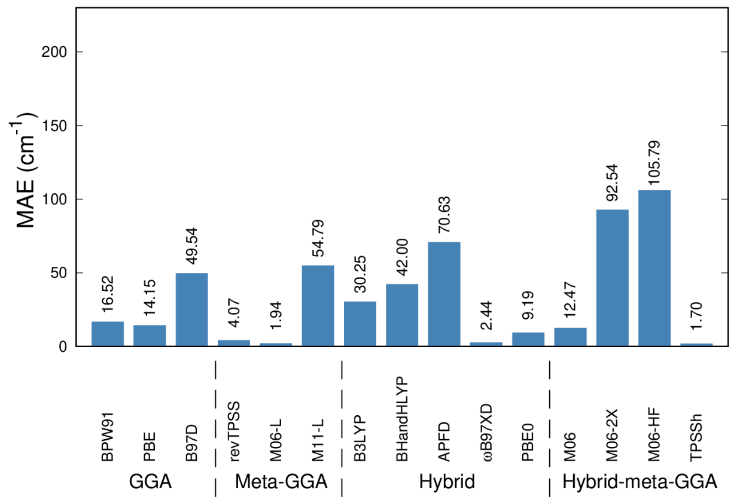
**

d) 6-311+G(d,p)

**
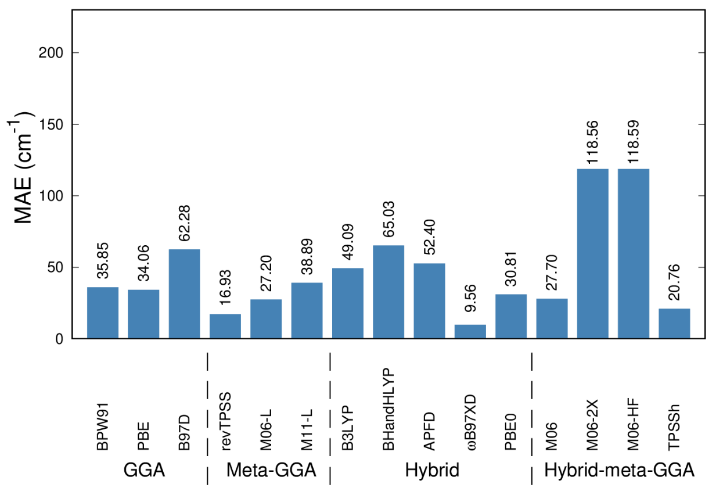
**

**Figure SI12.** MAEs for the vibrational constants of ${CuO}^{-}$ calculated for each one of the DFAs. Experimental value: 739 cm^-1^.[3] All values in cm^-1^.

**Dissociation energies MAEs**

$${Cu}_{2} \to2 Cu$$

| **Type DFA** | **DFA** | **Def2-SVP** | **Def2-TZVP** | **6-31+G(d,p)** | **6-311+G(d,p)** | |
| --- | --- | --- | --- | --- | --- | --- |
| **GGA** | BPW91 | 54.84 (11.35) | 44.92 (1.42) | 46.46 (2.97) | 45.11 (1.61) |  |
|  | PBE | 59.37 (15.88) | 48.19 (4.69) | 49.97 (6.47) | 48.65 (5.15) |  |
|  | B97D | 66.82 (23.32) | 51.42 (7.93) | 54.00 (10.51) | 53.08 (9.59) |  |
| **Meta-GGA** | revTPSS | 62.04 (18.55) | 49.28 (5.79) | 51.03 (7.53) | 50.10 (6.60) |  |
|  | M06-L | 58.85 (15.36) | 51.02 (7.52) | 46.82 (3.33) | 49.45 (5.95) |  |
|  | M11-L | 45.98 (2.48) | 44.94 (1.44) | 53.50 (10.01) | 46.55 (3.06) |  |
| **Hybrid** | B3LYP | 50.33 (6.84) | 41.49 (2.00) | 43.01 (0.49) | 41.59 (1.91) |  |
|  | BHandHLYP | 40.41(3.08) | 34.56 (8.93) | 36.05 (7.44) | 34.61 (8.88) |  |
|  | APFD | 55.76 (12.27) | 47.39 (3.90) | 49.41 (5.91) | 47.89 (4.40) |  |
|  | ωB97XD | 50.72 (7.22) | 45.49 (2.00) | 44.55 (1.06) | 44.95 (1.46) |  |
|  | PBE0 | 47.76 (4.27) | 40.62 (2.87) | 42.17 (1.32) | 40.87 (2.62) |  |
| **Hybrid**  **meta-GGA** | M06 | 133.33(89.84) | 46.89 (3.40) | 47.78 (4.28) | 46.83 (3.34) |  |
|  | M06-2X | 44.68 (1.19) | 37.73 (5.77) | 34.01 (9.48) | 35.95 (7.54) |  |
|  | M06-HF | 95.03 (51.53) | 16.11 (27.38) | 22.46 (21.04) | 20.70 (22.79) |  |
|  | TPSSh | 54.28 (10.79) | 43.77 (0.28) | 45.84 (2.34) | 44.68 (1.19) |  |

Table SI1. Dissociation energies and (MAEs) with respect to the experimental value of the ${Cu}_{2}$ specie. Experimental value: 43.49±0.60 kcal/mol.[5] All data in kcal/mol.

$${Cu}_{2}^{+} \to{Cu}^{+}+Cu$$

| **Type DFA** | **DFA** | **Def2-SVP** | **Def2-TZVP** | **6-31+G(d,p)** | **6-311+G(d,p)** |
| --- | --- | --- | --- | --- | --- |
| **GGA** | BPW91 | 54.99 (15.48) | 47.15 (7.64) | 47.98 (8.48) | 46.80 (7.30) |
|  | PBE | 58.46 (18.96) | 49.48 (9.98) | 50.85 (11.35) | 49.54 (10.03) |
|  | B97D | 53.95 (14.44) | 45.72 (6.21) | 46.99 (7.49) | 46.02 (6.52) |
| **Meta-GGA** | revTPSS | 59.70 (20.20) | 49.83 (10.32) | 50.90 (11.39) | 49.97 (10.47) |
|  | M06-L | 54.89 (15.39) | 49.04 (9.53) | 41.43 (1.92) | 43.91 (4.40) |
|  | M11-L | 44.86 (5.36) | 40.21 (0.71) | 42.84 (3.34) | 39.71 (0.20) |
| **Hybrid** | B3LYP | 49.02 (9.52) | 42.61 (3.11) | 43.55 (4.05) | 42.41 (2.91) |
|  | BHandHLYP | 41.86 (2.35) | 37.96 (1.55) | 38.72 (0.78) | 37.79 (1.71) |
|  | APFD | 53.11 (13.61) | 46.67 (7.17) | 47.65 (8.15) | 46.67 (7.17) |
|  | ωB97XD | 45.98 (6.48) | 42.12 (2.62) | 40.12 (0.62) | 40.79 (1.28) |
|  | PBE0 | 47.92 (8.42) | 42.40 (2.90) | 43.06 (3.56) | 42.28 (2.78) |
| **Hybrid**  **meta-GGA** | M06 | 89.60 (50.09) | 42.66 (3.16) | 39.66 (0.16) | 40.72 (1.22) |
|  | M06-2X | 43.23 (3.73) | 41.93 (2.43) | 39.34 (0.16) | 39.81 (0.31) |
|  | M06-HF | 76.40 (36.90) | 39.62 (0.12) | 44.25 (4.75) | 42.88 (3.37) |
|  | TPSSh | 52.97 (13.47) | 44.90 (5.39) | 46.12 (6.61) | 45.23 (5.73) |

Table SI2. Dissociation energies and (MAEs) with respect to the experimental value of the ${Cu}_{2}^{+}$ specie. Experimental value: 39.50±0.58 kcal/mol.[5] All data in kcal/mol.

$${Cu}_{2}^{-} \to{Cu}^{-}+Cu$$

| **Type DFA** | **DFA** | **Def2-SVP** | **Def2-TZVP** | **6-31+G(d,p)** | **6-311+G(d,p)** |
| --- | --- | --- | --- | --- | --- |
| **GGA** | BPW91 | 50.73 (12.91) | 39.37 (1.55) | 39.83 (2.01) | 39.23 (1.41) |
|  | PBE | 53.80 (15.98) | 41.60 (3.78) | 41.89 (4.07) | 41.29 (3.47) |
|  | B97D | 50.02 (12.20) | 38.01 (0.19) | 38.44 (0.62) | 37.66 (0.16) |
| **Meta-GGA** | revTPSS | 56.45 (18.63) | 43.53 (5.71) | 44.09 (6.27) | 43.73 (5.91) |
|  | M06-L | 53.32 (15.50) | 39.39 (1.57) | 39.78 (1.96) | 41.24 (3.42) |
|  | M11-L | 35.13 (2.69) | 34.52 (3.30) | 39.75 (1.93) | 36.07 (1.75) |
| **Hybrid** | B3LYP | 42.30 (4.48) | 34.14 (3.68) | 34.10 (3.72) | 33.17 (4.65) |
|  | BHandHLYP | 33.15 (4.67) | 29.12 (8.70) | 28.79 (9.03) | 27.75 (10.07) |
|  | APFD | 48.77 (10.95) | 40.51 (2.69) | 41.08 (3.26) | 40.26 (2.44) |
|  | ωB97XD | 39.36 (1.54) | 33.82 (4.00) | 32.84 (4.98) | 32.71 (5.11) |
|  | PBE0 | 42.10 (4.28) | 35.84 (1.98) | 35.68 (2.14) | 35.00 (2.82) |
| **Hybrid**  **meta-GGA** | M06 | 84.70 (46.88) | 36.45 (1.37) | 38.73 (0.91) | 37.69 (0.13) |
|  | M06-2X | 37.85 (0.03) | 33.65 (4.17) | 30.10 (7.72) | 29.17 (8.65) |
|  | M06-HF | 63.18 (25.36) | 31.17 (6.64) | 33.40 (4.42) | 30.27 (7.55) |
|  | TPSSh | 48.57 (10.75) | 38.99 (1.17) | 39.21 (1.39) | 38.75 (0.93) |

Table SI3. Dissociation energies and (MAEs) with respect to the experimental value of the ${Cu}_{2}^{-}$ specie. Experimental value: 37.82±3.46 kcal/mol.[6] All data in kcal/mol.

$$CuO \to Cu+O$$

| **Type DFA** | **DFA** | **Def2-SVP** | **Def2-TZVP** | **6-31+G(d,p)** | **6-311+G(d,p)** |
| --- | --- | --- | --- | --- | --- |
| **GGA** | BPW91 | 77.28 (7.15) | 70.16 (0.04) | 73.90 (3.77) | 71.25 (1.13) |
|  | PBE | 81.69 (11.56) | 73.99 (3.87) | 77.96 (7.84) | 75.21 (5.09) |
|  | B97D | 77.29 (7.16) | 68.03 (2.10) | 72.19 (2.06) | 70.05 (0.08) |
| **Meta-GGA** | revTPSS | 77.96 (7.84) | 69.38 (0.74) | 73.37 (3.24) | 70.66 (0.53) |
|  | M06-L | 77.45 (7.32) | 69.73 (0.40) | 70.45 (0.32) | 68.83 (1.30) |
|  | M11-L | 69.53 (0.60) | 66.03 (4.10) | 70.34 (0.21) | 67.78 (2.35) |
| **Hybrid** | B3LYP | 65.59 (4.53) | 60.75 (9.38) | 64.02 (6.11) | 61.72 (8.40) |
|  | BHandHLYP | 49.68 (20.44) | 48.06 (22.07) | 51.03 (19.09) | 48.90 (21.23) |
|  | APFD | 69.33 (0.80) | 65.38 (4.75) | 68.71 (1.42) | 41.14 (28.99) |
|  | ωB97XD | 65.18 (4.95) | 61.93 (8.20) | 64.82 (5.31) | 62.90 (7.23) |
|  | PBE0 | 62.64 (7.49) | 58.87 (11.26) | 62.31 (7.82) | 59.83 (10.30) |
| **Hybrid**  **meta-GGA** | M06 | 105.55(35.42) | 58.88 (11.25) | 63.24 (6.88) | 60.43 (9.70) |
|  | M06-2X | 55.67 (14.46) | 54.72 (15.41) | 54.47 (15.66) | 53.90 (16.23) |
|  | M06-HF | 73.99 (3.86) | 42.34 (27.78) | 47.30 (22.83) | 45.40 (24.73) |
|  | TPSSh | 69.72 (0.41) | 63.23 (6.89) | 67.13 (3.00) | 64.59 (5.53) |

Table SI4. Dissociation energies and (MAEs) with respect to the experimental value of the $CuO$ specie. Experimental value: 70.13±0.69 kcal/mol.[5] All data in kcal/mol.

$${CuO}^{+} \to{Cu}^{+}+O$$

| **Type DFA** | **DFA** | **Def2-SVP** | **Def2-TZVP** | **6-31+G(d,p)** | **6-311+G(d,p)** |
| --- | --- | --- | --- | --- | --- |
| **GGA** | BPW91 | 44.97 (13.87) | 43.16 (12.06) | 46.18 (15.08) | 41.69 (10.59) |
|  | PBE | 49.59 (18.49) | 46.75 (15.65) | 50.00 (18.90) | 45.34 (14.24) |
|  | B97D | 38.62 (7.52) | 21.77 (9.33) | 39.22 (8.12) | 19.95 (11.15) |
| **Meta-GGA** | revTPSS | 44.76 (13.66) | 42.55 (11.45) | 45.69 (14.59) | 41.24 (10.14) |
|  | M06-L | 37.96 (6.86) | 39.78 (8.68) | 37.30 (6.20) | 34.78 (3.68) |
|  | M11-L | 29.48 (1.62) | 28.24 (2.86) | 31.39 (0.29) | 26.83 (4.27) |
| **Hybrid** | B3LYP | 33.60 (2.50) | 30.66 (0.44) | 33.27 (2.17) | 29.44 (1.66) |
|  | BHandHLYP | 20.24 (10.86) | 18.07 (13.03) | 19.46 (11.64) | 17.20 (13.90) |
|  | APFD | 36.34 (5.24) | 34.79 (3.69) | 37.09 (5.99) | 21.06 (10.04) |
|  | ωB97XD | 27.88 (3.22) | 26.76 (4.34) | 27.26 (3.84) | 12.34 (18.76) |
|  | PBE0 | 29.51 (1.59) | 27.86 (3.24) | 30.23 (0.87) | 26.63 (4.47) |
| **Hybrid**  **meta-GGA** | M06 | 28.50 (2.60) | 26.55 (4.55) | 24.99 (6.11) | 23.92 (7.18) |
|  | M06-2X | 22.92 (8.18) | 20.75 (10.35) | 19.97 (11.13) | 18.51 (12.59) |
|  | M06-HF | 19.00 (12.10) | 14.95 (16.15) | 18.06 (13.04) | 15.71 (15.39) |
|  | TPSSh | 36.29 (5.19) | 34.42 (3.32) | 37.37 (6.27) | 33.25 (2.15) |

Table SI5. Dissociation energies and (MAEs) with respect to the experimental value of the ${CuO}^{+}$ specie. Experimental value: 31.10±2.80 kcal/mol.[4] All data in kcal/mol.

$${CuO}^{-} \to{Cu}^{-}+O$$

| **Type DFA** | **DFA** | **Def2-SVP** | **Def2-TZVP** | **6-31+G(d,p)** | **6-311+G(d,p)** |
| --- | --- | --- | --- | --- | --- |
| **GGA** | BPW91 | 76.54 (4.83) | 73.51 (1.79) | 86.52 (14.80) | 84.57 (12.85) |
|  | PBE | 79.49 (7.77) | 76.50 (4.78) | 90.14 (18.42) | 87.76 (16.04) |
|  | B97D | 64.87 (6.85) | 64.42 (7.30) | 77.71 (6.00) | 75.50 (3.78) |
| **Meta-GGA** | revTPSS | 75.19 (3.48) | 71.72 (0.00) | 84.91 (13.19) | 82.37 (10.65) |
|  | M06-L | 70.26 (1.46) | 64.91 (6.81) | 79.29 (7.57) | 77.04 (5.32) |
|  | M11-L | 60.61 (11.11) | 61.64 (10.08) | 72.05 (0.33) | 71.41 (0.31) |
| **Hybrid** | B3LYP | 57.50 (14.22) | 59.86 (11.86) | 71.34 (0.38) | 68.89 (2.83) |
|  | BHandHLYP | 27.98 (43.73) | 35.82 (35.89) | 45.50 (26.22) | 42.54 (29.18) |
|  | APFD | 60.56 (11.16) | 63.70 (8.02) | 75.06 (3.34) | 72.72 (1.00) |
|  | ωB97XD | 53.12 (18.59) | 56.46 (15.26) | 68.76(2.96) | 65.71 (6.01) |
|  | PBE0 | 54.25 (17.47) | 57.20 (14.52) | 68.65 (3.07) | 65.99 (5.73) |
| **Hybrid**  **meta-GGA** | M06 | 56.24 (15.48) | 54.08 (17.64) | 67.62 (4.10) | 64.34 (7.38) |
|  | M06-2X | 32.82 (38.90) | 46.01 (25.70) | 53.57 (18.15) | 49.70 (22.02) |
|  | M06-HF | 12.30 (59.42) | 41.80 (29.92) | 46.64 (25.08) | 44.62 (27.10) |
|  | TPSSh | 65.18 (6.54) | 64.69 (7.03) | 76.64 (4.92) | 74.21 (2.49) |

Table SI6. Dissociation energies and (MAEs) with respect to the experimental value of the ${CuO}^{+}$ specie. Experimental value: 71.72±3.46 kcal/mol.[3] All data in kcal/mol.

**MAEs for dimerization energies of Cu(II)/Indo**

| **Type DFA** | **DFA** | **Def2-SVP** | | **Def2-TZVP** | | **6-31+G(d,p)** | | **6-311+G(d,p)** | |
| --- | --- | --- | --- | --- | --- | --- | --- | --- | --- |
|  |  | **gas** | **sol** | **gas** | **sol** | **gas** | **sol** | **gas** | **sol** |
| **GGA** | BPW91 | 10.11 | 9.11 | 7.09 | 7.49 | 3.09 | 3.29 | 2.39 | 3.19 |
|  | PBE | 16.71 | 16.11 | 0.51 | 0.91 | 3.11 | 3.11 | 2.61 | 2.01 |
|  | B97D | 24.01 | 20.71 | 8.71 | 9.41 | 11.41 | 10.61 | 12.11 | 9.21 |
| **Meta-GGA** | revTPSS | 18.81 | 15.01 | 2.81 | 0.41 | 7.31 | 5.31 | 6.81 | 5.31 |
|  | M06-L | 26.11 | 24.11 | 10.91 | 12.41 | 12.61 | 12.11 | 13.21 | 12.71 |
|  | M11-L | 25.21 | 15.51 | 2.31 | 3.79 | 8.71 | 1.19 | 10.01 | 2.31 |
| **Hybrid** | B3LYP | 13.51 | 13.11 | 0.41 | 0.39 | 1.91 | 1.01 | 2.01 | 0.69 |
|  | BHandHLYP | 20.51 | 15.81 | 5.61 | 3.11 | 8.31 | 6.21 | 9.31 | 5.21 |
|  | APFD | 31.01 | 22.71 | 16.71 | 10.11 | 20.31 | 11.71 | 20.91 | 12.81 |
|  | ωB97XD | 25.21 | 21.61 | 9.01 | 9.61 | 12.81 | 12.51 | 15.21 | 11.11 |
|  | PBE0 | 18.41 | 16.21 | 4.11 | 3.91 | 6.11 | 5.21 | 7.71 | 6.21 |
| **Hybrid**  **meta-GGA** | M06 | 28.21 | 26.21 | 11.81 | 13.11 | 14.61 | 13.61 | 17.61 | 16.61 |
|  | TPSSh | 16.41 | 14.51 | 1.21 | 1.01 | 4.21 | 1.21 | 4.21 | 1.81 |

Table SI7. MAEs of $\Delta_{r}G_{dim}^{*}$ in gas and solution phase (ethanol). Experimental value: -5.59 kcal/mol.[7] All data in kcal/mol.

**References**

1. Ho J, Ervin KM, Lineberger WC (1990) Photoelectron spectroscopy of metal cluster anions: Cu−n, Ag−n, and Au−n. J Chem Phys 93:6987–7002. https://doi.org/10.1063/1.459475

2. Sappey AD, Harrington JE, Weisshaar JC (1989) Resonant two‐photon ionization‐photoelectron spectroscopy of Cu2: Autoionization dynamics and Cu+2 vibronic states. Journal of Chemical Physics 91:3854–3868

3. Ferrão LFA, Roberto-Neto O, Machado FBC (2008) Electronic structure of CuXy (X = B, C, N, O, F; y = 0, +1, −1). Int J Quantum Chem 108:2512–2522

4. Rezabal E, Gauss J, Matxain JM, et al (2011) Quantum chemical assessment of the binding energy of CuO+. J Chem Phys 134 6:064304

5. Parry IS, Hermes AC, Kartouzian A, Mackenzie SR (2014) Imaging the photodissociation dynamics of neutral metal clusters: copper dimer, Cu2, and copper oxide, CuO. Phys Chem Chem Phys 16:458–466. https://doi.org/10.1039/C3CP53214C

6. Spasov VA, Lee TH, Ervin KM (2000) Threshold collision-induced dissociation of anionic copper clusters and copper cluster monocarbonyls. Journal of Chemical Physics 112:1713–1720

7. Reyes-García LI, Moya-Hernández R, Rojas-Hernández A, et al (2021) Stability constants and molecular modeling of Cu(II)/AcO and Cu(II)/diclofenac complexes in ethanol. Polyhedron 209:115486. https://doi.org/https://doi.org/10.1016/j.poly.2021.115486
